# Cost-effectiveness of fenofibrate versus standard care for reducing the progression of diabetic retinopathy: an economic evaluation based on data from the LENS trial

**DOI:** 10.1111/dme.70098

**Published:** 2025-07-03

**Authors:** Graham Scotland, Mekazin Tsehaye, Caroline Styles, Jennifer Logue, Emily Sammons, Mohammed Zayed, Jonathan Emberson, Rachel Wade, Karl Wallendszus, Will Stevens, Rosanna Cretney, Simon Harding, Graham Leese, Gemma Currie, Jane Armitage, David Preiss, for the LENS collaborative group (see appendix)

**Affiliations:** 1 Health Economics Research Unit, University of Aberdeen, Polwarth Building, Foresterhill, Aberdeen, UK; 2 Queen Margaret Hospital, Dunfermline, Fife, UK; 3 Faculty of Health and Medicine, University of Lancaster, Lancaster, UK; 4 Clinical Trial Service Unit and Epidemiological Studies Unit, Nuffield Department of Population Health, University of Oxford, Oxford, UK; 5 Department of Eye and Vision Science, University of Liverpool and St. Paul’s Eye Unit, Liverpool University Hospitals NHS Foundation Trust, Liverpool, UK; 6 Molecular and Clinical Medicine, University of Dundee, Dundee, UK; 7 School of Cardiovascular & Metabolic Health, University of Glasgow, Glasgow, UK

**Keywords:** Diabetic Retinopathy, Fenofibrate, Cost-Effectiveness Analysis

## Abstract

**Aims:**

The LENS trial demonstrated that fenofibrate slowed progression of diabetic retinopathy compared to placebo in participants with early diabetic eye disease. We assessed its cost-effectiveness for reducing progression of diabetic retinopathy versus standard care from a UK National Health Service perspective.

**Methods:**

Resource use and outcome data were collected over follow-up for participants enrolled in LENS. Mean costs were compared at two years and per 6-months follow-up (median 4.0 years). Within trial cost-effectiveness was assessed in terms of incremental cost per case of referable disease averted. A microsimulation model, with inputs derived primarily from LENS trial data, was used to assess the incremental cost per quality adjusted life year (QALY).

**Results:**

Fenofibrate resulted in a mean (95% confidence interval) reduction in health service costs of -£254 (-1,062 to 624) at two years and -£101 (-243 to 42) per 6-months follow-up. This was accompanied by a 4.4% (1.3% to 8.0%) absolute reduction in any referable diabetic retinopathy or treatment thereof at two years, and a 27% (9%-42%) relative reduction over follow-up. Modelled over ten years, fenofibrate use cost an additional £6 per patient for an expected QALY gain of 0.02, costing £406 per QALY versus standard care under base case assumptions. The probability of cost-effectiveness varied from 70%-79% at a threshold of £20,000 per QALY, depending on the price discount applied to anti-VEGF drugs.

**Conclusions:**

Fenofibrate is likely to offer a cost-effective treatment for slowing progression of any diabetic retinopathy in people with early to moderate diabetic retinopathy or maculopathy.

## Introduction

Diabetic retinopathy and maculopathy are common microvascular complications of diabetes mellitus (DM)^1^ and remain prominent causes of visual impairment and blindness,^2,3^ resulting in significant costs to society and health-related quality of life (HRQoL) losses for patients.

The National Health Service (NHS) in Scotland has operated its Diabetic Eye Screening (DES) programme for over 15 years. In Scotland, screening outcomes are assigned based on the Scottish grading scheme (Supplementary Table 1). When retinal screening identifies that a patient has progressed referable background (R3) or proliferative disease (R4), patients are referred for specialist assessment and monitoring, and potentially treatment with scatter-peripheral laser photocoagulation (PRP). Patients who develop referable maculopathy (M2), accompanied by reduced visual acuity (≤75 Early Treatment of Diabetic Retinopathy Study (ETDRS) letters), are assessed using optical coherence tomography (OCT) and offered treatment with intravitreal injections if centre involving diabetic macular oedema (DMO) is detected.

Whilst treatments such as PRP and intravitreal injections reduce the risk of visual impairment, they impart a substantial economic burden,^4^ and residual risks of visual impairment remain following treatment.^5,6^ This highlights the potential clinical and economic value of oral treatments that can slow progression towards the later stages of disease.

Two cardiovascular disease (CVD) prevention trials in people with type two diabetes have shown promising effects of the lipid-lowering drug fenofibrate on diabetic retinopathy.^7,8^ The FIELD study reported that fenofibrate reduced the hazard of laser treatment for any retinopathy by 31% (95% CI: 26%-44%) compared to placebo. The ACCORD-Eye study reported a 40% (95% CI: 13%-58%) reduction in the composite outcome of laser treatment, vitrectomy or 3-step progression on the ETDRS scale. However, most patients in these trials had no retinopathy at baseline and had lipids and HbA1c within pre-specified limits. Consequently, the results may lack generalisability to those with clinically detectable early disease. Furthermore, these results came from subsidiary analyses, so may represent chance findings. The randomised double-masked placebo-controlled Lowering Events in Non-proliferative retinopathy in Scotland (LENS) trial was designed to address this evidence gap. It reported a 27% (95% CI: 9%-42%) reduction in the hazard of progression to referable diabetic retinopathy or maculopathy, or treatment thereof, over a median period of 4.0 years in participants with early diabetic eye disease.^9^ This paper reports on the cost-effectiveness analyses conducted as part of the trial.

## Methods

The economic evaluation was conducted from an NHS perspective, in accordance with a prespecified Health Economics Analysis Plan (HEAP) which was finalised before unblinding of the data (available from the authors). It included a within trial cost-effectiveness analysis based on individual participant data, and a model-based cost-utility analysis with inputs and assumptions informed by analysis of the trial dataset. The maximum follow-up duration available for all LENS trial participants was only two years. This was considered too early to adequately capture quality adjusted life year (QALY) gains associated with a preventive treatment to slow progression of diabetic retinopathy. It was therefore specified in the HEAP that the within trial analysis would focus on costs and clinical outcomes, and that cost-utility would be undertaken using a decision analytic modelling framework with inputs informed by analysis of the trial data, supplemented by external evidence on longer-term implications of referable disease. These complimentary analyses are described below, with further methodological details provided in online appendices 1 and 2, respectively.

The LENS trial design and clinical findings are published elsewhere.^10,9^ Participants were adults with diabetes and observable retinopathy, defined as mild background retinopathy in both eyes or observable background retinopathy in one/both eyes, or observable maculopathy in one/both eyes (supplementary Table 1), and were randomised (1:1) to study intervention or matched placebo. The study intervention was 145mg (nanoparticle) fenofibrate, once daily for those with estimated glomerular filtration rate (eGFR) ≥60mL/min/1.73m^2^, and every other day for those with eGFR 30-59mL/min/1.73m^2^. The primary outcome was progression to referable diabetic retinopathy or maculopathy, or any of retinal laser therapy, vitrectomy or intra-vitreal injection of medication indicated for diabetic retinopathy/maculopathy. Informed consent was obtained from participants in accordance with good clinical practice. People with diabetes played an important role in the design and conduct of the LENS trial, although not specifically the health economic analyses based on it.

### Resource use and costs

Individual patient level health care resource use data were collected using linked health care records and trial-specific patient questionnaires (administered at baseline and six-monthly thereafter) and valued in 2022/23 prices (Supplementary Table 2).^11^

The required quantity of study drug was dispensed six-monthly. Since the dose and formulation of fenofibrate used in the LENS trial is not available in the UK NHS, the drug tariff price (reflecting the price paid by the NHS for community prescribed medicines) for a bioequivalent 200mg (micronised) formulation was applied.^12,13^ Those on a reduced dose due to impaired renal function were assumed to require one 200mg capsule every other day. Tests for renal function, creatinine, HbA1c, lipids and urine albumin creatinine ratio were costed using unit costs of laboratory services.^14^ Nurse and general practitioner time required to obtain samples and review results were included.^11^

Hospital outpatient and inpatient activity in a priori specified specialties of interest were obtained from routinely collected Scottish Morbidity Records (SMR00 and SMR01) and serious adverse event reports and combined with nationally representative unit costs by clinical specialty.^14^ Specialties of interest included: General Medicine; Acute Medicine; Cardiology; Endocrinology and Diabetes; Diabetes; Renal Medicine; Vascular Surgery; Cardiac Surgery; and Ophthalmology.

Community prescribed medicines for the management of diabetes, lipids, and blood pressure were captured through record linkage to the Prescribing Information System (PIS) for Scotland. These data were combined with NHS drug tariff prices to estimate costs of relevant medication use.^12^

Published unit costs were applied to retinal screening episodes,^15,16^ obtained by linkage to DES records. Hospital referral and treatment for diabetic retinopathy and DMO were primarily captured in the cost of outpatient and inpatient activity. Where patients initiated anti-VEGF injections for DMO, as indicated in the study database, it was necessary to assume anti-VEGF drug costs would be incurred at the frequency observed over a two-year period in a published NHS cohort study: 6.3 and 2.9 injections in years one and two respectively.^17^

### Health outcomes

Given a modest absolute difference in progression event rate between treatment arms, infrequent HRQoL measurement, and limited duration of follow-up to fully capture the impact of delayed progression on HRQoL, the within trial cost-effectiveness analysis focussed on the primary outcome as the measure of effect. Quality adjusted life years (QALYs) were modelled using health state utility values informed by analysis of EQ-5D-5L data collected at baseline, two years post-randomisation, and study exit, supplemented by published literature. EQ-5D-5L response data were mapped to the preferred EQ-5D-3L value set using the algorithm developed by Hernández Alava et al.^18^

### Economic model

**Figure 1:**
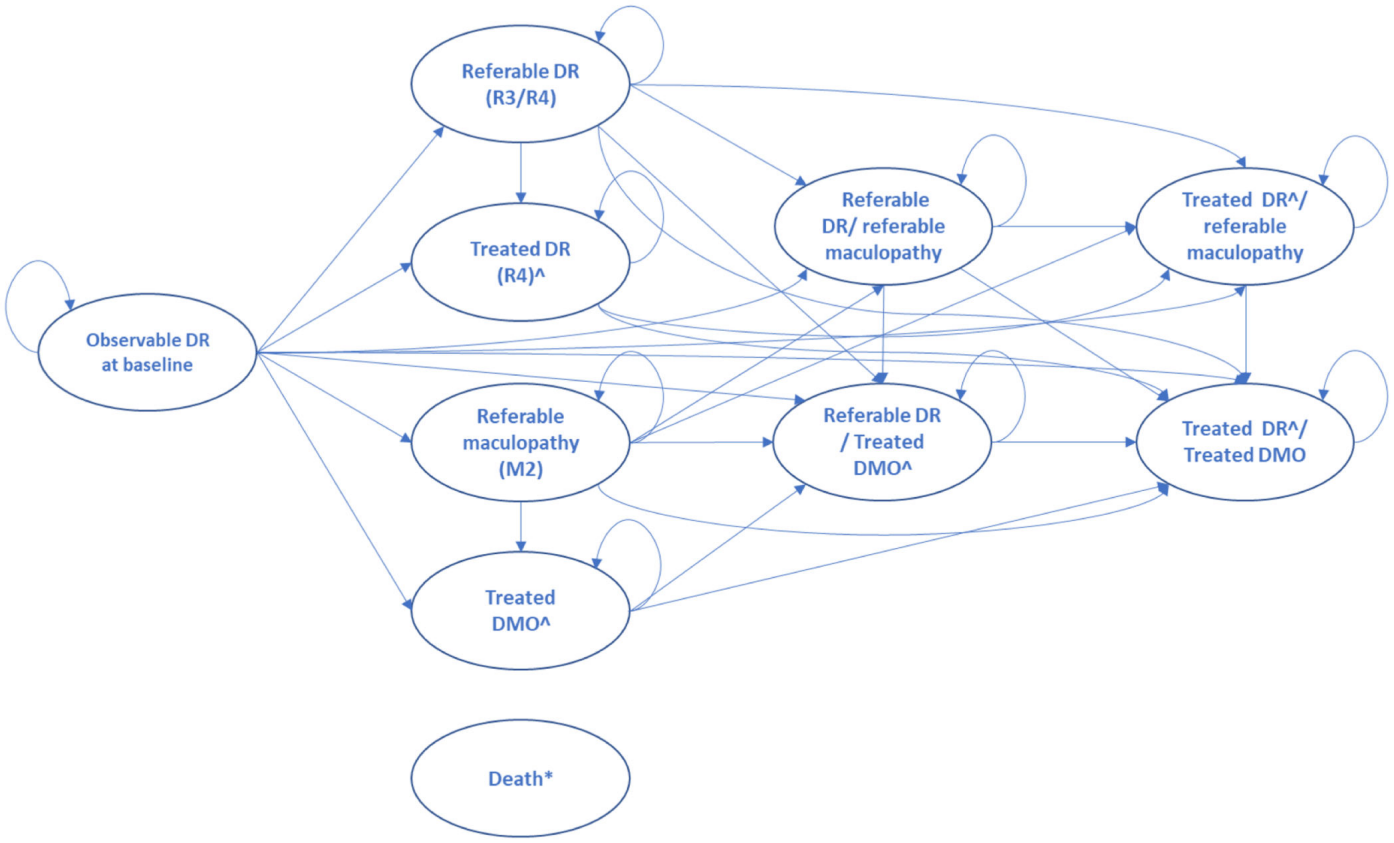
Schematic of the Markov microsimulation model *Death can be entered from any state in each model cycle; DMO, diabetic macular oedema; DR (diabetic retinopathy; ^Treatment costs and post treatment changes in visual acuity and associated quality of life captured within these states

A microsimulation model (simulating individual patients) was developed to determine the incremental cost per QALY gained with fenofibrate versus standard care. The model used a Markov structure with ten health states (Figure 1) and a six-month cycle length with half-cycle correction. The approach was chosen to allow for efficient tracking of time to and time since the development of both diabetic retinopathy (R3/R4) and maculopathy (M2), allowing time dependency to be built into the model for both these pathways using a relatively simple structure. It also facilitated exploration of heterogeneity in cost-effectiveness based on the baseline covariates collected for LENS trial participants. Simulated individuals, with baseline characteristics drawn from those of LENS trial participants, start the model in the non-referable state. Their passage through the health states was governed by six-monthly transition probabilities estimated from parametric survival analysis of the LENS trial time to event data (see analysis methods). Mortality was based on UK life tables, combined with standardised mortality ratios for type 1 and type 2 diabetes.^19,20^ The base case used a ten-year time horizon. Costs and QALYs accruing beyond year one were discounted at 3.5% per year, in line with the NICE reference case.^21^

Health state utility values for the model states were estimated from the LENS trial EQ-5D data (see analysis methods). With relatively few patients in the LENS trial requiring retinal treatment, it was assumed the estimated utility decrement of progression captured the impact of early referable disease on HRQoL. Further utility decrements following initiation of retinal treatment were therefore modelled through expected changes in visual acuity (VA)^17,22,6^ linked to changes in health state utility.^23^

Costs in the model include fenofibrate acquisition, background health care use (hospital activity in specialties of interest other than ophthalmology, biochemistry monitoring, and other prescribed medications), retinal screening, and monitoring and treatment of referable disease. Six-monthly background health care and screening costs were informed by regression analysis of the LENS trial dataset (see analysis methods).

Monitoring and treatment for those developing referable disease were aligned with routine NHS practice, informed by published studies and clinical expert opinion.^24,17,22,25,26^ It was conservatively assumed that treatment was for unilateral disease in the base case. Multipliers of 1.9 and 1.4, based on clinical expert opinion that 90% and 40% would ultimately require bilateral treatment for proliferative DR and DMO respectively, were applied to treatment-specific costs in sensitivity analyses. Scottish unit costs were applied to all diabetic retinopathy treatment and monitoring activity in the base case. English NHS costs were tested in sensitivity analysis (Supplementary Table 2).^27^

### Analysis methods

#### Within trial comparison of costs and outcomes

Statistical analyses were performed using STATA statistical software (Version 16.1).^28^ Costs were summarised by treatment allocation using the mean and SD. Cost-effectiveness was initially assessed at two-years post-randomisation in terms of the incremental cost per case of referable disease averted. A generalised linear model (GLM) with inverse gaussian distribution and power (-0.1) link function, adjusted for minimisation covariates, was used to estimate the mean difference in cost at this time point. The chosen specification was informed by Modified Parks, Pregibond link, and Modified Hosmer and Lemeshow tests.^29^ The corresponding probabilities of being free from progression were estimated by Weibull survival analysis following the assessment of proportional hazards and the visual and statistical fit of alternative parametric survival models (online appendix 2). Non-parametric bootstrapping was used to characterise the uncertainty surrounding the joint mean difference in costs and effects at two years.^30^ The LENS trial achieved greater than 99% follow up, with only two participants not completing their final follow-up, but with censoring dates beyond two years. With the reliance on population-based registries for resource use data, supplemented with 6-monthly phone-based questionnaires, it was assumed all relevant resource use data were captured up to the time of censoring. Thus, strategies for dealing with missing cost and clinical outcome data were deemed unnecessary. Costs incurred beyond year one of follow-up were discounted at a rate of 3.5%.^21^ The clinical progression outcome was not discounted, due to a lack guidance on how to discount dichotomous clinical outcomes informed by time to event analysis.

A further cost comparison, using all available follow-up data, was conducted using interval-based methods to account for censoring.^30^ Generalised estimating equations (GEE) were used to estimate the mean difference in six-monthly cost between treatment groups by interval, assuming a gamma family distribution with log link function.

#### Economic model inputs

Derived parameter inputs to the economic model are presented in the results section, with methodological details provided in online appendix 2. Cause-specific hazards of transitioning from the observable state to R3/R4 with or without referable maculopathy/DMO, and referable maculopathy/DMO alone, were estimated using proportional hazards parametric models (online appendix 2).^31^ The final models included minimisation covariates, allowing calculated risks to match individual characteristics of simulated patients. These were used to derive transition probabilities in the economic model, accounting for competing risk. An exponential function was selected to model progression to referable retinopathy (R3/R4), and a Weibull function was used for progression to referable maculopathy/DMO.^32^ Whilst cause-specific hazards for different types of progression were modelled, the overall effect of fenofibrate on the primary composite outcome (estimated by Weibull survival analysis) was applied to both types of event in the base case, in line with prior evidence supporting a common treatment effect across retinopathy and maculopathy outcomes.^7^ Proportional hazards were maintained over the model time horizon in the base case.

Transitions between post-referral states were also informed by LENS trial time to event data, using time of progression to referable retinopathy or referable maculopathy as time zero in the calculation of time at risk (online appendix 2). With small numbers of events informing these transitions, rates were assumed to follow exponential distributions, with effects of fenofibrate applied as hazard ratios. An exception was made for the transition from referable DR (R3/R4) to treatment for proliferative DR, where independent lognormal distributions were fitted to capture apparent plateauing in the Kaplan Meier data.

A mixed model for repeated measures (MMRM) was used to estimate EQ-5D health state utility values for the model health states, which makes use of all available data at each time point and provides valid estimates under the assumption that missing response data are missing at random conditional on the observed outcomes and covariates included in the model (online appendix 2).^33^ The preferred specification, with lowest Akaike and Bayesian information Criteria (AIC and BIC), used pooled data and included a random effect for individual and fixed effects for baseline EQ-5D score, any referrable disease, time from baseline, and baseline age. The estimated model was applied to generate individual health state utilities in the decision model, allowing utility to vary by time, health state and included covariates.

GEEs were used to estimate the effect of modelled health states on six-monthly background health care costs and screening costs, with indicators included for referable maculopathy, treated maculopathy, referable diabetic retinopathy, and treated diabetic retinopathy.

#### Economic Model analysis

Microsimulation was used to propagate the passage of 500,000 individuals - with baseline characteristics resampled from those of LENS trial participants - through the model one at a time. The model was also analysed probabilistically. Input parameters used to populate the model were assigned theoretical probability distributions reflecting uncertainty due to sampling variation (detailed in the results).^34^ Given the computational burden of running the probabilistic analysis with microsimulation, this relied on 1000 draws from assigned second order distributions (outer loop), with 50,000 simulated individuals per draw (inner loop).^35^ Deterministic scenario analysis explored the impact of varying structural and methodological assumptions. Further analysis explored heterogeneity in cost-effectiveness by selected baseline characteristics.

## Results

1151 participants were randomised, 576 to fenofibrate and 575 to placebo. Baseline demographics and clinical characteristics were well-balanced between treatment arms (Table 1).

### Within trial comparison of costs and outcomes

Health care resource use and associated health service costs are summarised by treatment allocation in Supplementary Tables 3 and 4, respectively. No substantial differences were observed across the categories of resource use. Intervention costs were higher in the fenofibrate arm, offset by lower outpatient and inpatient costs in specialities of interest.

At two years fenofibrate was weakly dominant, linked to a non-statistically significant reduction in mean health service costs and a statistically significant increase in the probability of remaining free of referable disease (Table 2).

**Figure 2:**
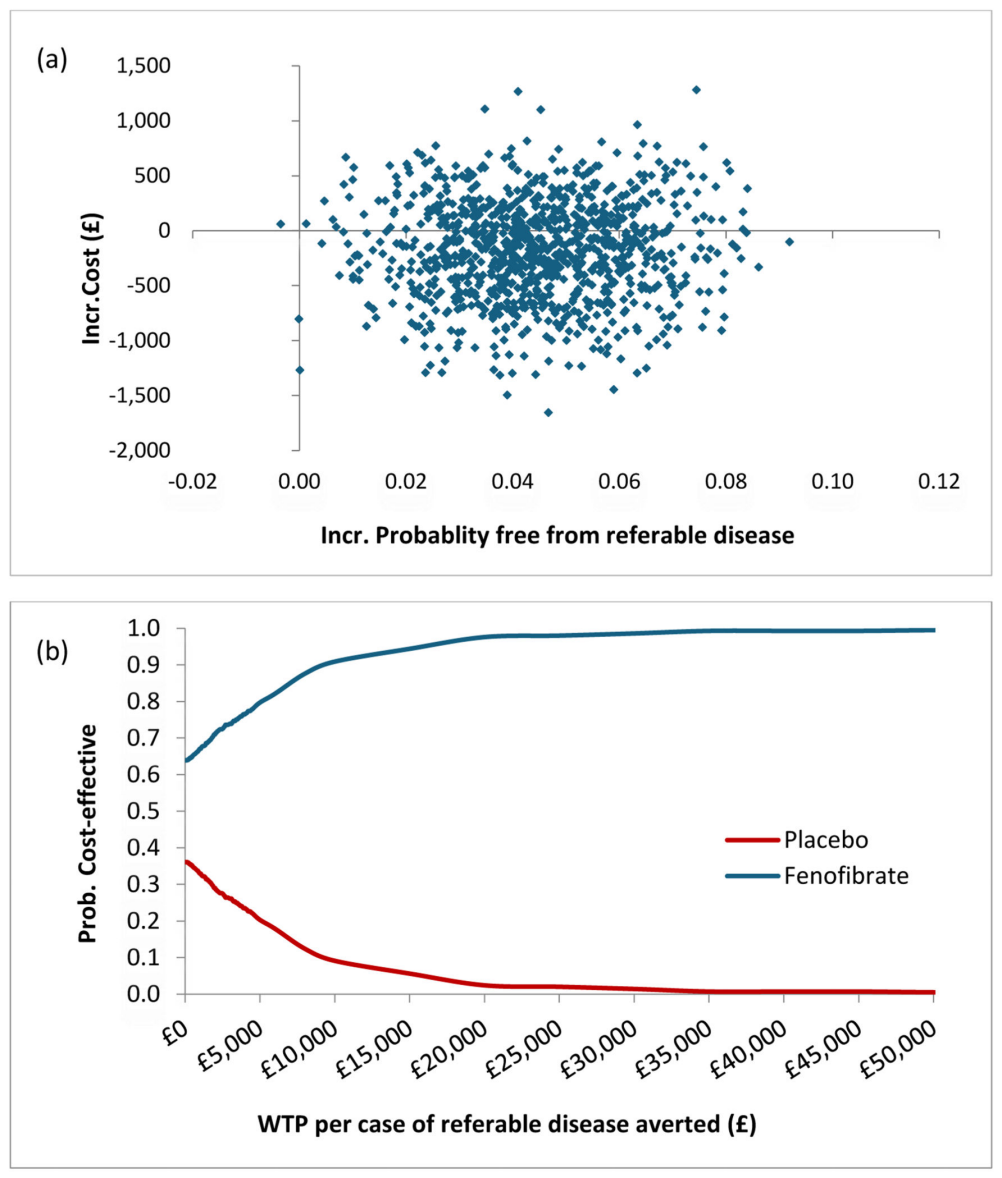
Trial based cost-effectiveness scatter plot for fenofibrate versus standard care (placebo) (a) and the corresponding cost-effectiveness acceptability curves (b) Incr., incremental; Prob., Probability; WTP, Willingness to pay

Based on 1000 bootstrapped replicates of the analysis models, most of the incremental cost and effect pairs (64%) lie in the southeast quadrant of the cost-effectiveness plane (Figure 2a), where fenofibrate is considered dominant (less costly and more effective). This translates into fenofibrate having the higher probability of being cost-effective compared to standard care across all thresholds of willingness to pay per case of referable disease averted (Figure 2b). This finding remained robust to changes to the price of anti-VEGF drugs (supplementary Table 5) in the inclusion of all hospital costs.

The interval-based cost-analysis, using all follow-up data, showed a non-significant reduction in six-monthly costs in the fenofibrate arm (supplementary Table 6). Combined with evidence of a significant reduction in the risk of progression, this corroborates the findings of the two-year incremental analysis.

### Economic model inputs derived from analysis of the trial data

Derived model input parameters and their assigned distributions are provided in Supplementary Table 7. Based on analysis of individual participant data, no statistically significant differences in background health care costs were identified by treatment allocation or progression status. Therefore, the model analysis assumed that only fenofibrate and its impact on progression of diabetic retinopathy influence differences in cost between the model treatment arms. Modelled differences in QALYs are influenced by disease progression, which was estimated to result in an EQ-5D utility decrement of -0.020 (95% CI, -0.042, 0.003) based on the MMRM analysis, and further decrements associated with modelled post-treatment visual acuity losses.

### Model-based cost-effectiveness results

**Figure 3:**
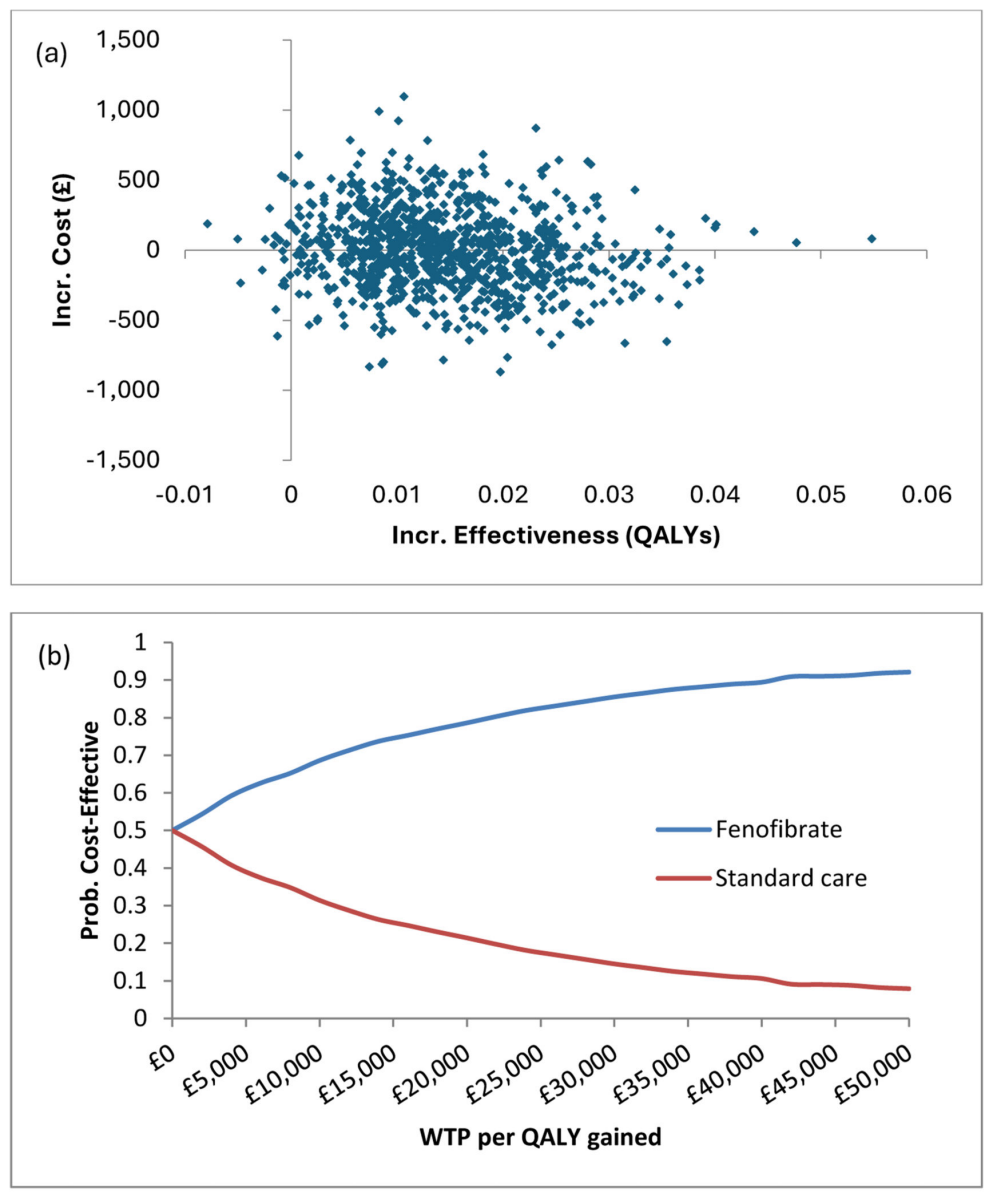
Model based cost-effectiveness scatter plot for fenofibrate versus standard care (a) and the corresponding cost-effectiveness acceptability curves (b) Incr., incremental; Prob., Probability; WTP, Willingness to pay; QALY, quality adjusted life year

Comparison of the model output against the Kaplan Meier data for the primary outcome and any treatment for diabetic retinopathy, suggested a satisfactory fit to the observed trial data (Supplementary Figures 1 and 2). Based on these extrapolations, fenofibrate was associated with a small increase in cost and a small QALY gain over the ten-year time horizon (Table 4). The probabilistic analysis indicated an 79-86% chance of fenofibrate being cost-effective at thresholds of £20-£30,000 per QALY gained (Figure 3).

Supplementary Table 8 provides details and justification for a range of scenario analysis explored, with results provided in Table 4. Over longer time horizons, the incremental cost of fenofibrate reduced and the QALY gain increased, resulting in it becoming dominant. Reducing the price of anti-VEGF drugs (aflibercept) increased the ICER but it remained below cost-effectiveness thresholds applied in the UK NHS.^21^ Inflating PRP and anti-VEGF treatment costs, to account for plausible percentages of people requiring bilateral treatment based on clinical expert opinion, resulted in fenofibrate becoming dominant. The probability of fenofibrate being cost-effectiveness at a threshold of £20,000 per QALY, varied from 70% to 79% at aflibercept price discounts of 70% and zero respectively (supplementary Figure 3).

Exploration of heterogeneity in the economic model showed the ICERs to be generally favourable across subgroups, but particularly in those with type 1 diabetes, HbA1c ≥64 mmol/mol (DCCT 8%) and observable maculopathy at baseline (Supplementary Table 9).

## Discussion

Based on the analysis of individual patient cost data, fenofibrate treatment (at its generic NHS price) resulted in non-significant reductions in health service costs: -£254 (-1062 to 624) at two years and -£101 (-243 to 42) per six months of follow-up. The observed direction of effect is consistent with the significant effect of fenofibrate on progression of diabetic retinopathy, and possible reductions in hospital resource use in other relevant specialties (Supplementary Table 4). Based on conservative modelling over a ten year time horizon, assuming fenofibrate only influences future health care costs by delaying diabetic retinopathy progression, fenofibrate was associated with a small increase in cost (+£6) for a small QALY gain (0.02), with an ICER below thresholds used to guide UK NHS decision making.^21^ Subgroup analysis suggests that it may be particularly cost-effective in people with type 1 diabetes and those at higher risk of progression (HbA1c ≥64 mmol/mol (DCCT 8%), or those with observable maculopathy at baseline).

Modelling the effect of fenofibrate over longer time horizons resulted in it becoming dominant, as more resource savings and health benefits accrue. There is uncertainty relating to the confidential price the NHS pays for anti-VEGF treatment for DMO, but our findings remained robust to a range of plausible discounted prices. Further, the base case ICER assumes that all treatment is for unilateral disease, which is a conservative assumption favouring standard care.

A strength of our study relates to the efficient design of the LENS trial, in which follow-up of resource use and outcomes benefited from record linkage to population-based registries and administrative datasets with high coverage. This allowed us to capture individual resource use comprehensively, avoiding issues of missing data and recall bias that can affect trials that rely more heavily on patient self-report. The LENS trial randomised approximately 8-10% of eligible patients across mainland Scotland based on key eligibility criteria, facilitating generalisability of the results. Further, since the DES programme in Scotland is similar to others across the UK, the cost-effectiveness findings should be broadly applicable. Indeed, the application in our economic model of English NHS costs to referable disease monitoring and treatment indicates this is likely to be the case. The chosen microsimulation approach allowed the monitoring and treatment pathways to be modelled precisely with respect to time since treatment initiation.

The study did not capture potentially relevant costs falling on social services or patients and their families. However, assuming retinal treatment and/or visual losses due to diabetic retinopathy impact these broader costs, the adopted health service perspective is likely conservative. With the reliance on electronic linked routine data for follow-up, we were unable to capture primary care resource use, although primary care costs were factored in for requested biochemistry tests. The lack of difference in serious adverse events and hospital activity in medical specialties of interest, suggests differences in primary care attendance would also be unlikely. Another limitation arises from the duration of follow-up (median 4.0 years) not being long enough to detect material differences in HRQoL.^9^ This was overcome by using an economic model to link progression and subsequent modelled reductions in visual acuity with HRQoL decrements estimated from the LENS trial data and external literature. However, the model may fail to fully capture HRQoL benefits of delayed progression over the lifetime of patients. A further limitation is that we did not routinely capture the number of anti-VEGF injections or laser sessions received by those initiating treatment in the trial. However, assumptions about treatment courses were informed by observational NHS cohort studies. Results should therefore be applicable to routine NHS practice.

Two CVD prevention trials have studied the effects of fenofibrate in people with type two diabetes in subsidiary analyses,^7,8^ and identified retinopathy progression benefits in keeping with findings of the LENS trial. We are aware of only one prior study that has assessed the cost-effectives of fenofibrate for slowing the progression of diabetic retinopathy, based on data from these trials (available as an abstract).^36^ Despite estimating greater QALY gains associated with fenofibrate use, this study reported higher incremental costs and higher ICERs than our study, but still found it to be cost-effective from an Australian health service perspective. The differences may be due to changes in the price of fenofibrate (or differences between countries), higher risks of progression to referable disease in the LENS trial cohort compared to these other trials, and/or changes to the treatment pathways for DMO. Other studies have reported fenofibrate to be cost-effective or leading to lower net health care costs in the long-term based on modest lipid lowering effects,^37–39^ but such effects are conservatively not included in our economic model.

In summary, treatment of patients with early diabetic retinopathy with fenofibrate can be expected to generate future resource savings in ophthalmology outpatient services, offsetting additional fenofibrate acquisition costs and maintaining the visual function and health status of patients with less need for invasive treatment. Thus, fenofibrate is likely to offer a cost-effective use of NHS resources.

## Supplementary Material

Appendix

## Figures and Tables

**Table 1:** Participant Baseline characteristics

Characteristics	Fenofibrate (N=576)		Placebo (N=575)	
	n/mean	% /SD	n/mean	%/SD
Age, years (mean, SD)	60.8	12.4	60.6	12.3
Age group (n, %)				
<30	16	3	14	2
≥ 30, <50	81	14	82	14
≥50, <70	347	60	346	60
≥ 70	132	23	133	23
Sex, female (n, %)	156	27	156	27
Race (n, %)				
White	567	98	558	97
Other	9	2	17	3
Type of diabetes (n, %)				
Type 1	154	27	151	26
Type 2	421	73	423	74
Other	1	0	1	0
Estimated glomerular filtration rate (n, %)				
<60 ml/min/1.73 m2	130	23	131	23
≥60 ml/min/1.73 m2	446	77	444	77
HbA1c group (n, %)				
<64 mmol/mol (DCCT <8%)	251	44	252	44
≥64 mmol/mol (DCCT ≥8%)	251	44	249	43
Unknown	74	13	74	13
Baseline medication (n, %)				
Statin	425	74	429	75
Insulin	256	44	249	43
Non-insulin glucose-lowering therapy	396	69	389	68
Renin-angiotensin system inhibitor	345	60	341	59
Retinopathy grading (worse eye) (n, %)				
No retinopathy (R0)	5	1	4	1
Mild background retinopathy (R1)	562	98	564	98
Observable background retinopathy (R2)	9	2	7	1
Maculopathy grading (worse eye) (n, %)				
No maculopathy (M0)	517	90	515	90
Observable diabetic maculopathy (M1)	59	10	60	10
Retinal laser treatment or intravitreal injections or vitrectomy (n, %)	53	9	59	10
Cardiovascular disease	103	18	96	17

SD, standard deviation; HbA1c, glycated haemoglobin; DCCT, Diabetes Control and Complications Trial

**Table 2:** Two-year incremental comparison of costs and proportion free from referable retinopathy and referable maculopathy

	Cost (£), mean (95% CI)*		Free from referable DR. proportion (95% CI)*		
Treatment group	Mean	Incremental	Total	Incremental	ICER (£)
Fenofibrate	3,566 (3,088 to 4,364)	-254 (-1,062 to 624)	0.867 (0.844 to 0.889)	0.044 (0.013 to 0.08)	Dominant
Placebo	3,820 (3,052 to 4,749)		0.823 (0.796 to 0.847)		

* Costs and QALYs adjusted from minimisation covariates which included categories of age at randomisation (<30; ≥30 <50; ≥50 <70; ≥70 years), type of diabetes (type 1; type 2; other), sex (male; female), HbA1c (<64; ≥64mmol/mol; unknown), renal function (<60; ≥60 mL/min/1.73m2), statin use (Yes; No), baseline retinopathy grade (mild; observable; other), and baseline maculopathy grade (no maculopathy; observable maculopathy). CI, confidence interval; ICER, Incremental cost-effectiveness ratio, expressed for fenofibrate versus placebo; Dominant, an intervention that is both more effective and less costly than a comparator.

**Table 3:** Model-based incremental cost per QALY gained over a ten-year time horizon

Comparator	Cost (£), mean		QALYs, mean		ICER (£)
	Total	Incremental	Total	Incremental	
**Base Case (Deterministic**)					
Standard care	14,229		5.423		
Fenofibrate	14,234	6	5.437	0.015	406
**Base case (Probabilistic)**					
Standard care	14,275		5.419		
Fenofibrate	14,283	9	5.434	0.014	613

ICER, incremental cost-effectiveness ratio; QALY, quality-adjusted life year

**Table 4:** Model based cost-effectiveness scenario analysis results (fenofibrate versus standard care)

Parameter / assumption	Base case	Scenario	Incremental cost (£)	Incremental QALYs	ICER (£)
**Base case**			6	0.015	406
1. Time horizon	10 years	a) 5 years	82	0.007	12,216
		b) 20 years	-73	0.024	Dominant
		c) 30 years	-79	0.028	Dominant
2. Time to referable DR and referable maculopathy	Single overall treatment effect	Cause specific treatment effects	40	0.014	2,895
3. Time to referable maculopathy	Weibull curve	Exponential curve	-2	0.015	Dominant
		Log logistic	-30	0.018	Dominant
4. Time to referable retinopathy (R3/R4)	Exponential curve	Gompertz	-2	0.015	Dominant
		Log logistic	4	0.015	290
5. Treatment effect of fenofibrate	Continuous proportional hazards	Waned from 5 years	111	0.011	10,014
6. Effect of fenofibrate on post-progression transitions	Estimated from the data	Assume no effect	105	0.014	7,724
7. Time from DR to DR treatment curve	Lognormal	Exponential	3	0.016	165
		Weibull	10	0.014	685
		Lognormal (SC), exponential (feno)	8	0.014	593
		Exponential (SC), Gompertz (feno)	11	0.015	774
8. Modelled visual losses	Applied to WSE	Applied to BSE	6	0.015	399
		Applied to both eyes	6	0.016	366
9. Disutility of VA loss	Based on EQ-5D	Based on VFQ-25	6	0.017	351
10. Treatment costs for DMO and proliferative DR	Applied for unilateral disease	Inflated by 40% and 90% respectively, to account for bilateral treatment	-95	0.015	Dominant
11. Anti-VEGF drug costs	NHS indicative price of aflibercept (£816 per vial)	a. Discounted by 30%	67	0.015	4,588
		b. Discounted by 40%	87	0.015	5,982
		c. Discounted by 50%	107	0.015	7,376
		d. Discounted by 60%	127	0.015	8,770
		e. Discounted by 70%	148	0.015	10,163
12. Fenofibrate dosing for reduced eGFR	200 mg every second day	67 mg every day	115	0.015	7,888
13. Ophthalmology referral/treatment costs	Scottish speciality costs	English HRG based NHS reference costs	44	0.015	2,997
14. Fenofibrate price	Scottish drug tariff (£4.77)	English drug tariff (£2.69)	-108	0.015	Dominant
15. Ophthalmology referral/treatment costs and fenofibrate price	Scottish specialty cost and Scottish drug tariff	English HRG based NHS reference costs and English drug tariff	-70	0.015	Dominant
16. Fenofibrate additional monitoring costs	Assumed absorbed as part of routine diabetes management	Additional check of renal function 1-2 months after starting fenofibrate (accounting for GP and nurse time)	25	0.015	1,731
17. Fenofibrate prescribing costs	Assumed absorbed as part of routine management of diabetes	Allocate an additional GP appointment to initiate treatment	55	0.015	3,780
18. Background health state costs	Equalised across treatment arms	Estimated non-significant difference in 6-montly costs by treatment arm applied	-1,694	0.015	Dominant
19. Combined scenario (16 + 17)	Fenofibrate initiation and monitoring	Allocate a full additional GP appointment and a check of renal function 1-2 months after starting	75	0.015	5,176
20. Combined scenario (9 + 10e)	Anti-VEGF prices and bilateral treatment	70% discount in anti-VEGF price, accounting for bilateral treatment	103	0.015	7,116
21. Combined scenario (9 + 10c)	Anti-VEGF prices and bilateral treatment	50% discount in anti-VEGF price, accounting for bilateral treatment	47	0.015	3,213

ICER, incremental cost-effectiveness ratio; QALY, quality-adjusted life year; BSE, best seeing eye; DMO, diabetic macular oedema; DR, diabetic retinopathy; EQ-5D, Euroqual 5-dimension; R3, severe background retinopathy; R4, proliferative retinopathy; SC, standard care; feno, fenofibrate; VA, visual acuity; WSE, worst seeing eye; eGFR, estimated glomerular filtration rate; VHQ-25, Visual Function Questionnaire – 25 items

## What is already known?

- Diabetic retinopathy is a leading cause of visual impairment
- In the LENS trial, fenofibrate reduced progression to referable diabetic retinopathy or maculopathy, or treatment thereof, in people with early diabetic retinopathy
- There is a lack of cost-effectiveness evidence to support adoption in the UK NHS.

## What this study has found?

- The cost of fenofibrate was offset by downstream savings.
- Visual function and health-related quality-of-life can be maintained with a reduced need for invasive treatment.

## What are the implications of the study?

- Clinicians should consider prescribing fenofibrate as a cost-effective treatment for people with early diabetic retinopathy.
