# Supplementary material for: Cost‐effectiveness of fenofibrate versus standard care for reducing the progression of diabetic retinopathy: An economic evaluation based on data from the LENS trial
Source: Diabet Med. 2025 Jul 3;42(9):e70098. doi: 10.1111/dme.70098 (PMC7617897; doi:10.1111/dme.70098)
Supplement: Supplementary file 1 — Data S1. [file DME-42-e70098-s003.docx]

Supplementary online appendix

This appendix has been provided by the authors to give readers additional information about their work:

Cost-effectiveness of fenofibrate versus standard care for reducing the progression of diabetic retinopathy: an economic evaluation based on data from the LENS trial.

Graham Scotland, Mekazin Tsehaye, Caroline Styles, Jennifer Logue, Emily Sammons, Mohammed Zayed, Jonathan Emberson, Rachel Wade, Karl Wallendszus, Will Stevens, Rosanna Cretney, Simon Harding, Graham Leese, Gemma Currie, Jane Armitage, David Preiss, for the LENS collaborative group

#

Contents

[List of Figures 3](#_Toc194333801)

[List of Tables 5](#_Toc194333802)

[Supplementary Appendix 1 – within trial analysis methods 6](#_Toc194333803)

[Further details of costing methods 6](#_Toc194333804)

[Analysis methods 8](#_Toc194333805)

[Assessment of proportional hazards assumption for the primary clinical outcome 8](#_Toc194333806)

[Parametric curve selection for the primary clinical outcome 10](#_Toc194333807)

[Supplementary Appendix 2 – Decision modelling methods 13](#_Toc194333808)

[Economic model structure 13](#_Toc194333809)

[Deriving transition probabilities 13](#_Toc194333810)

[Observable diabetic retinopathy to referable retinopathy or referable maculopathy 13](#_Toc194333811)

[Time to referable maculopathy or treatment for diabetic macular oedema 14](#_Toc194333812)

[Time to referable retinopathy (R3/R4) 18](#_Toc194333813)

[Models of time to referable retinopathy (R3/R4) and referable maculopathy, inclusive of covariates 18](#_Toc194333814)

[Combining cause specific hazards for first progression event 18](#_Toc194333815)

[Post progression transition probabilities 23](#_Toc194333816)

[Referable maculopathy to treatment for diabetic macular oedema 24](#_Toc194333817)

[Referable DR (R3/R4) to treatment for referable DR 27](#_Toc194333818)

[Referable maculopathy to referable diabetic retinopathy (R3/R4) 30](#_Toc194333819)

[Referable diabetic retinopathy (R3/R4) to referable maculopathy 33](#_Toc194333820)

[Health state utility impact of progression 36](#_Toc194333821)

[Modelled long-term changes in visual acuity and impact on quality of life 36](#_Toc194333822)

[Modelled resource use assumptions 38](#_Toc194333823)

[Microsimulation model analysis 38](#_Toc194333824)

[Model validation 39](#_Toc194333825)

## List of Figures

[Figure 1 Kaplan Meier plot for the LENS trial primary outcome*.* 9](#_Toc194334353)

[Figure 2 Log cumulative hazard plot for the LENS trial primary outcome 9](#_Toc194334354)

[Figure 3 Schoenfeld residuals plot for the LENS trial primary outcome, with respect to treatment allocation 10](#_Toc194334355)

[Figure 4 Parametric survival curves for the LENS trial primary outcome in the placebo (Standard care) arm 11](#_Toc194334356)

[Figure 5 Parametric survival curves for the LENS trial primary outcome in the fenofibrate arm 11](#_Toc194334357)

[Figure 6 Log cumulative hazard plot for time to referable maculopathy 15](#_Toc194334358)

[Figure 7 Parametric survival curve fits for time to referable maculopathy in the placebo (standard care) arm 16](#_Toc194334359)

[Figure 8 Parametric survival curve fits for time to referable maculopathy in the fenofibrate arm 17](#_Toc194334360)

[Figure 9 Preferred curve fits for time to referable maculopathy 17](#_Toc194334361)

[Figure 10 Log cumulative hazard plot for time to referable retinopathy or laser treatment 20](#_Toc194334362)

[Figure 11 Parametric survival curve fits for time to referable diabetic retinopathy in the placebo (standard care) arm 21](#_Toc194334363)

[Figure 12 Parametric survival curve fits for time to referable diabetic retinopathy in the fenofibrate arm 22](#_Toc194334364)

[Figure 13 Selected curve fits for time to referable diabetic retinopathy (R3/R4) 22](#_Toc194334365)

[Figure 14 Log cumulative hazard plot for referable maculopathy to treatment for diabetic macular oedema 24](#_Toc194334366)

[Figure 15 Parametric survival curve fits for referable maculopathy to treatment for diabetic macular oedema in the placebo (standard care) arm 25](#_Toc194334367)

[Figure 16 Parametric survival curve fits for referable maculopathy to treatment for diabetic macular oedema in the fenofibrate arm 26](#_Toc194334368)

[Figure 17 Selected curve fits for referable maculopathy to treatment for diabetic macular oedema 26](#_Toc194334369)

[Figure 18 Log cumulative hazard plot for referable retinopathy to treatment for referable retinopathy 27](#_Toc194334370)

[Figure 19 Parametric survival curve fits for time from referable retinopathy to treatment for diabetic retinopathy in the placebo (standard care) arm 28](#_Toc194334371)

[Figure 20 Parametric survival curve fits for time from referable retinopathy to treatment for diabetic retinopathy in the fenofibrate arm 29](#_Toc194334372)

[Figure 21 Selected curve fits for time from referable retinopathy (R3/R4) to treatment for diabetic retinopathy 29](#_Toc194334373)

[Figure 22 Log cumulative hazard plot for time from referable maculopathy to referable diabetic retinopathy 30](#_Toc194334374)

[Figure 23 Parametric survival curve fits for time from referable maculopathy to referable diabetic retinopathy in the placebo (standard care) arm 31](#_Toc194334375)

[Figure 24 Parametric survival curve fits for time from referable maculopathy to referable diabetic retinopathy in the fenofibrate arm 32](#_Toc194334376)

[Figure 25 Parametric survival curve fits for time from referable maculopathy to referable diabetic retinopathy 32](#_Toc194334377)

[Figure 26 Log cumulative hazard plot for time from referable retinopathy (R3/R4) to referable diabetic maculopathy 33](#_Toc194334378)

[Figure 27 Parametric survival curve fits for time from referable retinopathy to referable maculopathy in the placebo (standard care) arm 34](#_Toc194334379)

[Figure 28 Parametric survival curve fits for time from referable retinopathy to referable maculopathy in the fenofibrate arm 35](#_Toc194334380)

[Figure 29 Selected Parametric survival curve fits for time from referable retinopathy to referable maculopathy 35](#_Toc194334381)

## List of Tables

[Table 1 Proportional hazards test with respect to treatment allocation 10](#_Toc194334436)

[Table 2 Akaike and Bayesian information criteria for different parametric models fitted to the LENS primary outcome data 12](#_Toc194334437)

[Table 3 Proportional hazards test for time to referable maculopathy with respect to treatment allocation 15](#_Toc194334438)

[Table 4 Akaike and Bayesian information criteria of different parametric models fitted to time to referable maculopathy 16](#_Toc194334439)

[Table 5 Proportional hazards test for time to referable retinopathy (R3/R4) or laser with respect to treatment allocation 20](#_Toc194334440)

[Table 6 Akaike and Bayesian information criteria of different parametric models for time to referable retinopathy (R3/R4) 21](#_Toc194334441)

[Table 7 Proportional hazards test for referable maculopathy to treatment for diabetic macular oedema with respect to treatment allocation group 24](#_Toc194334442)

[Table 8 Akaike and Bayesian information criteria of different parametric models of time from referable maculopathy to treatment for diabetic macular oedema 25](#_Toc194334443)

[Table 9 Proportional hazards test for referable retinopathy to treatment for referable retinopathy with respect to treatment allocation group 27](#_Toc194334444)

[Table 10 Akaike and Bayesian information criteria of parametric models of time from referable retinopathy to treatment for referable retinopathy, fitted independently by treatment arm 28](#_Toc194334445)

[Table 11 Proportional hazards test for referable maculopathy to referable diabetic retinopathy by treatment allocation group 30](#_Toc194334446)

[Table 12 Akaike and Bayesian information criteria of different parametric models of time from referable maculopathy to referable diabetic retinopathy (R3/R4) 31](#_Toc194334447)

[Table 13 Proportional hazards test for time from referable retinopathy (R3/R4) to referable diabetic maculopathy, with respect to treatment allocation group 33](#_Toc194334448)

[Table 14 Akaike and Bayesian information criteria of different parametric models of time from referable diabetic retinopathy R3/R4) to referable maculopathy 34](#_Toc194334449)

[Table 15 Available EQ-5D data by time point 36](#_Toc194334450)

# Supplementary Appendix 1 – within trial analysis methods

## Further details of costing methods

In the LENS trial, drug safety was monitored by review of routinely collected blood tests (linked from SCI-Diabetes registry), which were requested at the discretion of participants’ GPs or hospital doctors. Requested monitoring tests for creatinine, HbA1c, lipids and UACR were costed based on published unit costs of laboratory services in Scotland.^1^ Five minutes of nurse time and five minutes of physician time, required to obtain samples and review results, were factored in for groups of tests by date of request.^2^

Retinal screening episodes (including digital retinal photography, slit-lamp examination, and OCT surveillance) were linked from NHS Scotland’s Diabetic Eye Screening (DES) Programme, and costed using previously published unit cost estimates, inflated to 2022/23 prices using the NHS Cost Inflation Index.^2^

Diabetic retinopathy related referrals and interventions were captured using linked data on ophthalmology outpatient visits (from SMR00) and admissions (SMR01), combined with data on diabetic retinopathy related interventions recorded in the study database. Ophthalmology hospital activity was costed using relevant Scottish Health Service costs by specialty and setting of care (outpatient, inpatient, day case).^1^ English specific NHS reference costs were tested in sensitivity analysis.^3^ The number of anti-vascular endothelial growth factor (VEGF) injections administered to those commencing treatment for diabetic macular oedema (DMO) were not routinely recorded. As these are normally delivered as a course of treatment, it was necessary to assume that they would be administered at the frequency observed over two-years in an observational NHS cohort study (6.3 treatments in year one, and 2.9 treatments in year two).^4^ This was further validated and considered conservative (less than expected based on recognised trial treatment protocols) based on clinical expert opinion. List prices for aflibercept treatment were applied in the base case.^5^ Since this treatment is available to the NHS at an undisclosed discounted price, sensitivity analysis explored the impact of reducing prices over a range of possible discounts.

Scottish health service costs were applied to all inpatient (SMR01) and outpatient hospital activity (SMR00) occurring post-randomisation up to the final follow-up date for each participant.^1^ Hospital activity was sub-divided into a priori specified clinically relevant specialties of interest (where activity could feasibly be affected by the study drug) and other specialties. Clinically relevant specialties included: General Medicine; Acute Medicine; Cardiology; Endocrinology and Diabetes; Diabetes; Renal Medicine; Vascular Surgery; Cardiac Surgery; and Ophthalmology. For the incremental cost-effectiveness analysis, we included the costs of all care episodes in clinically relevant specialties of interest.

An inpatient episode refers to a patient's stay in a specific hospital specialty, from admission to discharge (home, or to another consultant, specialty or hospital). If a patient transfers between specialties within the same hospital, they are counted as part of a continuous inpatient stay or hospital spell, with multiple episodes across different specialties. The national average unit cost per inpatient episode for each specialty of care, was sourced from Table R040 of the Scottish Health Service Cost Book.^1^ We used fixed and variable cost percentage splits, as reported by the Technical Advisory Group on Resource Allocation (TAGRA) in Scotland, when applying published unit costs to episodes of inpatient care. Practically, the percentage splits represent the proportion of speciality episode costs that are considered fixed, and applicable per episode for each speciality, and the proportion that is considered variable by length of stay under each speciality. The variable component was divided by the average length of stay for each speciality, as reported in Table R040 of the Cost Book, and used to cost observed admitted patient care on per diem basis. Day case and outpatient activity was costed using the national average costs by specialty from Tables R042 and R044 and of the cost book respectively.

Medicines prescribed in the community setting, related to the management of diabetes, lipids, and blood pressure, were captured through participant record linkage to the Prescribing Information System (PIS) for Scotland. Prescribed medicines were costed using Drug Tariff prices or NHS indicative prices for proprietary medicines.^5^ The linked dataset included community prescribing episodes for each patient that occurred during the LENS trial follow-up period (post-randomisation, up the date of final follow-up), with variables denoting the dispensing month, the approved drug name, and corresponding BNF code for each item prescribed. The unique name for each drug, along with its specifications, was obtained by mapping from the BNF code (BNF SNOMED mapping). Once the unique name and its specifications were determined, the cost of the appropriate dose for the indicated use was attached to each drug. For most drugs, it was assumed they were taken according to the recommended dose, starting from the first prescription date. Accordingly, an interval of time was calculated to derive the number of drug units required during the period between consecutive prescribing dates, and associated costs were then estimated using Drug Tariff prices or NHS indicative prices for proprietary medicines. In cases where the recommended dose was not provided in the BNF, the dose was sourced from relevant literature.

## Analysis methods

### Assessment of proportional hazards assumption for the primary clinical outcome

This within trial cost-effectiveness analysis relied on parametric analysis of the primary time to event outcome in the LENS trial, the composite of progression of observable DR to referable DR, or any of retinal laser therapy, vitrectomy or intra-vitreal injection of medication due to DR.

The proportional hazards assumption with respect to treatment allocation was assessed by visual inspection of Kaplan-Meier data (Figure 1), assessment of the log cumulative hazard plot (Figure 2) and the Schoenfeld residuals plot (Figure 3), and by testing for a non-zero slope in the relationship between the scaled Schoenfeld residuals and time (p-values less than 0.05 indicating sufficient evidence to reject the null hypothesis of proportional hazards). The Kaplan Meier curves separate early and remain apart over the duration of follow-up. Whilst the lines of the log cumulative hazard plots do cross, this relates to the early follow-up period, and thereafter they remain apart and relatively parallel. The Schoenfeld residuals show even scatter around zero, with no obvious pattern with respect to follow-up time, and insufficient evidence to reject the null hypothesis of a constant proportional effect of treatment with fenofibrate (p=0.34).

Figure 1 Kaplan Meier plot for the LENS trial primary outcome*.*

Figure 2 Log cumulative hazard plot for the LENS trial primary outcome

Figure 3 Schoenfeld residuals plot for the LENS trial primary outcome, with respect to treatment allocation

Table 1 Proportional hazards test with respect to treatment allocation

| Treatment | Rho | Chi-squared | Degrees freedom | P-value |
| --- | --- | --- | --- | --- |
| Fenofibrate | 0.055 | 0.92 | 1 | 0.3375 |

### Parametric curve selection for the primary clinical outcome

The support for proportional hazards placed an emphasis on assessing the fit of alternative proportional hazards models with a constant treatment effect expressed as a hazard ratio. However, accelerated failure time models were also explored. Model selection for the primary outcome was based on consideration of visual fit to the observed Kaplan Meier data (Figures 4 and 5), and statistical fit as assessed by Akaike and Bayesian information criteria (Table 2). All curves provided reasonable fit to the observed data and resulted in limited variability in referable disease-free projections. However, the Weibull model supported a diminishing hazard of progression over time and minimised the aggregate of AIC and BIC. It was therefore chosen as the preferred parametric model for estimating risk of progression for the trial-based cost-effectiveness analysis.

Figure 4 Parametric survival curves for the LENS trial primary outcome in the placebo (Standard care) arm

KM, Kaplan Meier; Ref, referable; DR, diabetic retinopathy

Figure 5 Parametric survival curves for the LENS trial primary outcome in the fenofibrate arm

KM, Kaplan Meier; Ref, referable; DR, diabetic retinopathy; Feno, fenofibrate

Table 2 Akaike and Bayesian information criteria for different parametric models fitted to the LENS primary outcome data

| **Parametric function** | **AIC** | **BIC** | **Average** |
| --- | --- | --- | --- |
| Exponential | 2028.23 | 2038.33 | 2033.28 |
| Weibull | 2024.89 | 2040.03 | 2032.46 |
| Generalized gamma | 2023.86 | 2044.06 | 2033.96 |
| Gompertz | 2030.02 | 2045.16 | 2037.59 |
| Lognormal | 2041.87 | 2057.01 | 2049.44 |
| Log Logistic | 2029.08 | 2044.22 | 2036.65 |

AIC, Akaike information criteria; BIC, Bayesian information criteria

# Supplementary Appendix 2 – Decision modelling methods

## Economic model structure

The model used a Markov structure with ten discrete health states reflecting the progression of diabetic retinopathy and its treatment: Observable diabetic retinopathy (bilateral R1, R2 or M1); referable retinopathy (R3 or R4); treated diabetic retinopathy; referable maculopathy (M2); treated diabetic macular oedema; referable retinopathy with referable maculopathy; treated diabetic retinopathy with referable maculopathy; referable retinopathy with treated macular oedema; treated diabetic retinopathy and treated macular oedema; and death (Figure 7). The model operates on a six-monthly cycle, consistent with the shortest screening interval that people with an R2 grade receive in the DES service in Scotland. Treatment for referable disease was modelled as observed in the trial in the “treated diabetic retinopathy” and “treated macular oedema” states. Following completion of expected treatment courses, patients remain in the treated states of the model incurring expected follow-up costs and changes in VA as informed by external literature. A microsimulation approach was used to enable tracking of treatment history and individual patient characteristics, which in turn inform expected costs and utility payoffs within health states.

## Deriving transition probabilities

Progression of patients through the model health states was based 6-monthly transition probabilities derived from parametric survival analysis of the LENS trial time to event outcomes.

### Observable diabetic retinopathy to referable retinopathy or referable maculopathy

Cause-specific hazards of progression from the baseline observable state to referable retinopathy (R3/R4) with or without referable maculopathy/DMO, and referable maculopathy/DMO alone, were estimated first. For each of these components of the primary progression outcome, proportionality of hazards with respect to treatment allocation was assessed by log-cumulative hazard plots and the Schoenfeld residuals proportional hazards test. Following this, six standard parametric curves were fitted, with treatment allocation group as a covariate. The resultant curve fits were assessed visually against the observed Kaplan-Meier data, and statistical fit was assess using the Akaike and Bayesian information criteria.

### Time to referable maculopathy or treatment for diabetic macular oedema

For progression to referable maculopathy/DMO alone, the lines of the log-cumulative hazards plot do cross early in the follow-up period (Figure 6) but thereafter remain apart and relatively parallel. Furthermore, the p-value from the proportional hazard test is non-significant (Table 3), suggesting insufficient evidence to reject the proportional hazards assumption. Visual inspection of the six parametric curves suggested a good fit for the Weibull, Gompertz and log logistic in the placebo arm (Figure 7) and reasonable fit to the fenofibrate arm (Figure 8). Based on aggregation of the AIC and BIC (Table 4), the Weibull distribution provided the best statistical fit of the six parametric distributions, supporting a diminishing hazard of progression over time, and providing a middle ground in terms of extrapolated outcomes. With the support for the proportional hazards assumption, the Weibull, as a proportional hazards model, was selected for deriving transitions by treatment arm in the base case model (Figure 9). The exponential and log-logistic were assessed as alternatives in scenario analysis.

Figure 6 Log cumulative hazard plot for time to referable maculopathy

Table 3 Proportional hazards test for time to referable maculopathy with respect to treatment allocation

|  | Chi-squared | Degrees freedom | P-value |
| --- | --- | --- | --- |
| Global test | 0.91 | 1 | 0.3402 |

Table 4 Akaike and Bayesian information criteria of different parametric models fitted to time to referable maculopathy

| **Parametric model** | **AIC** | **BIC** | **Average** |
| --- | --- | --- | --- |
| Exponential | 1794.56 | 1804.66 | 1799.61 |
| Weibull | 1789.72 | 1804.87 | 1797.29 |
| Generalized gamma | 1789.78 | 1809.98 | 1799.88 |
| Gompertz | 1795.87 | 1811.01 | 1803.44 |
| Lognormal | 1801.23 | 1816.38 | 1808.80 |
| Log Logistic | 1792.39 | 1807.53 | 1799.96 |

AIC, Akaike information criteria; BIC, Bayesian information criteria

Figure 7 Parametric survival curve fits for time to referable maculopathy in the placebo (standard care) arm

KM, Kaplan Meier; Ref, referable

Figure 8 Parametric survival curve fits for time to referable maculopathy in the fenofibrate arm

KM, Kaplan Meier; Ref, referable; Feno, fenofibrate

Figure 9 Preferred curve fits for time to referable maculopathy

KM, Kaplan Meier; Ref, referable; Feno, fenofibrate

### Time to referable retinopathy (R3/R4)

For progression to referable retinopathy (R3/R4) or laser treatment thereof, the same process was followed. The log-log plot (Figure 10) showed the curves to be somewhat intertwined, owing to relatively small number of events to inform hazards. However, the test of Schoenfeld residuals failed to reject the null hypothesis of proportional hazards (Table 5), and so proportional hazard models were prioritised in the selection of curves. Of the available models, the exponential was found to minimise both AIC and BIC (Table 6), provide a reasonable fit to the observed Kaplan-Meier data (Figure 11 and Figure 12), and provide a plausible middle ground in terms of extrapolated outcomes. It was therefore chosen to inform transitions from observable to referable diabetic retinopathy (R3/R4), or treatment thereof, in the base case model (Figure 13). The Gompertz and log-logistic were tested in scenario analyses.

Models of time to referable retinopathy (R3/R4) and referable maculopathy, inclusive of covariates.

The above section shows the alternative parametric curves by treatment allocation group. The microsimulation model, however, was developed with the functionality to include the effects of trial minimisation covariates on the hazards of these progression events. These were informed by parametric survival analysis with the minimisation variables included as covariates. The preferred Weibull distribution was used for progression to referable maculopathy, and the exponential function was used for the transition to referable retinopathy (R3/R4). The AIC and BIC also showed these to provide the best statistical fits, respectively, for the models including covariates. The covariates included categories of age at randomisation (<30; ≥30 <50; ≥50 <70; ≥70 years), type of diabetes (type 1; type 2; other), sex (male; female), HbA1c (<64; ≥64mmol/mol; unknown), renal function (<60; ≥60 mL/min/1.73m2), statin use (Yes; No), baseline retinopathy grade (mild; observable; none), and baseline maculopathy grade (no maculopathy; observable maculopathy). These more complex models were incorporated in the cost-effectiveness model, allowing the predicted progression risks to vary by these characteristics of simulated patients.

### Combining cause specific hazards for first progression event

The parameters of the selected Weibull (for time to referable maculopathy or treatment for DMO) and exponential (for time to referable retinopathy (R3/R4) or treatment thereof) functions were used to predict the cause specific hazards by treatment arm for these competing first progression events by 6-month intervals of follow-up time. These were then combined additively, to estimate the overall hazard of leaving the observable state by treatment allocation group, and then converted into overall time dependent transition probabilities of leaving the state, using the formula:

p(t) = 1-e^-rt^

Where p is the transition probability, t is the model cycle length (= 6 months), and r is the estimated average hazard rate by treatment arm. The overall transition probability for progression, was then distributed between the competing first progression events based on the ratio of their estimated cause specific hazards in each model cycle.

Whilst the analysis of cause specific hazards indicated a significant effect of fenofibrate on the hazard of first progression to referable maculopathy alone (Hazard Ratio, 95% CI: 0.66, 0.51 – 0.85), the effect on first progression to referable retinopathy (R3/R4) was non-significant and directionally favoured the placebo (standard care) arm; Hazard Ratio (95% CI), 1.31 (0.75 – 2.30). This is likely due to small numbers of referable retinopathy (R3/R4) events and is inconsistent with prior evidence suggesting a common treatment effect across the individual retinopathy and maculopathy components of the overall primary progression outcome.^6^ Therefore, the overall treatment effect, estimated from Weibull regression of the primary composite outcome (HR, 95% CI: 0.73, 0.58 - 0.92), was applied to both the maculopathy and retinopathy components in the base case analysis. The cause specific hazard ratios were applied in sensitivity analysis.

Figure 10 Log cumulative hazard plot for time to referable retinopathy or laser treatment

Table 5 Proportional hazards test for time to referable retinopathy (R3/R4) or laser with respect to treatment allocation

|  | Chi-squared | Degrees freedom | P-value |
| --- | --- | --- | --- |
| Global test | 0.04 | 1 | 0.8375 |

Table 6 Akaike and Bayesian information criteria of different parametric models for time to referable retinopathy (R3/R4)

| **Parametric model** | **AIC** | **BIC** | **Average** |
| --- | --- | --- | --- |
| Exponential | 502.77 | 512.87 | 507.82 |
| Weibull | 504.71 | 519.85 | 512.28 |
| Generalized gamma | 506.59 | 526.79 | 516.69 |
| Gompertz | 504.26 | 519.41 | 511.84 |
| Lognormal | 507.86 | 523.00 | 515.43 |
| Log Logistic | 504.83 | 519.98 | 512.40 |

AIC, Akaike information criteria; BIC, Bayesian information criteria

Figure 11 Parametric survival curve fits for time to referable diabetic retinopathy in the placebo (standard care) arm

KM, Kaplan Meier; DR, diabetic retinopathy

Figure 12 Parametric survival curve fits for time to referable diabetic retinopathy in the fenofibrate arm

KM, Kaplan Meier; DR, diabetic retinopathy; Feno, fenofibrate

Figure 13 Selected curve fits for time to referable diabetic retinopathy (R3/R4)

KM, Kaplan Meier; DR, diabetic retinopathy; Feno, fenofibrate

## Post progression transition probabilities

Transition probabilities between post-referral states were also informed by LENS trial time to event data, using time of progression to referable retinopathy or referable maculopathy as time zero in the calculation of time at risk. With relatively few events available to inform the distribution of the survivor functions, these event rates were generally assumed to follow exponential distributions, with the effect of fenofibrate applied as a hazard ratio. Nevertheless, the following sections provide the results of proportional hazards assessment and alternative parametric curve fits for each of the required transitions in turn:

- Time from first documented referable maculopathy to treatment for diabetic macular oedema (Figures 14-17 and Tables 7 and 8)
- Time from first documented referable retinopathy (R3/R4) to treatment for referable retinopathy (Figures 18-21 and Tables 9 and 10)
- Time from first document referable maculopathy to referable retinopathy (R3/R4) (Figures 22-25 and Tables 11 and 12)
- Time from first document referable retinopathy (R3/R4) to referable maculopathy. (Figures 26-29 and Tables 13 and 14)

An exception was made for the transition from referable DR (R3/R4) to treatment for referable DR, where independent lognormal distributions were fitted to capture apparent plateauing in the Kaplan Meier data (Figures 18-21 and Tables 9 and 10). Several alternative models were assessed in scenario analysis.

The transitions from referable maculopathy to referable retinopathy, and from referable retinopathy to referable maculopathy, were modelled independently of whether treatment for diabetic macular oedema or treatment for referable retinopathy had occurred, respectively. Similarly, the transitions from referable maculopathy to treatment for DMO, and from referable retinopathy to pan-retinal laser treatment, were modelled independently of each other.

Given the uncertainty surrounding post-progression transition probabilities, further scenario analysis explored the impact of equalising these between the treatment arms, i.e. assuming no effect of fenofibrate on these transition probabilities.

### Referable maculopathy to treatment for diabetic macular oedema

Figure 14 Log cumulative hazard plot for referable maculopathy to treatment for diabetic macular oedema

Table 7 Proportional hazards test for referable maculopathy to treatment for diabetic macular oedema with respect to treatment allocation group

|  | Chi-squared | Degrees of freedom | P-value |
| --- | --- | --- | --- |
| Global test | 0.00 | 1 | 0.9965 |

Table 8 Akaike and Bayesian information criteria of different parametric models of time from referable maculopathy to treatment for diabetic macular oedema

| **Parametric model** | **AIC** | **BIC** | **Average** |
| --- | --- | --- | --- |
| Exponential | 169.55 | 176.68 | 173.12 |
| Weibull | 167.58 | 178.28 | 172.93 |
| Generalized gamma | 169.41 | 183.67 | 176.54 |
| Gompertz | 171.41 | 182.10 | 176.75 |
| Lognormal | 168.42 | 179.12 | 173.77 |
| Log Logistic | 167.7559 | 178.4494 | 173.10 |

AIC, Akaike information criteria; BIC, Bayesian information criteria

Figure 15 Parametric survival curve fits for referable maculopathy to treatment for diabetic macular oedema in the placebo (standard care) arm

KM, Kaplan Meier; DMO, diabetic macular oedema

Figure 16 Parametric survival curve fits for referable maculopathy to treatment for diabetic macular oedema in the fenofibrate arm

KM, Kaplan Meier; DMO, diabetic macular oedema; Feno, fenofibrate

Figure 17 Selected curve fits for referable maculopathy to treatment for diabetic macular oedema

KM, Kaplan Meier; DMO, diabetic macular oedema; Feno, fenofibrate

### Referable DR (R3/R4) to treatment for referable DR

Figure 18 Log cumulative hazard plot for referable retinopathy to treatment for referable retinopathy

Table 9 Proportional hazards test for referable retinopathy to treatment for referable retinopathy with respect to treatment allocation group

|  | Chi-squared | Degrees of freedom | P-value |
| --- | --- | --- | --- |
| Global test | 6.61 | 1 | 0.010 |

Table 10 Akaike and Bayesian information criteria of parametric models of time from referable retinopathy to treatment for referable retinopathy, fitted independently by treatment arm

|  | **Placebo** | | | **Fenofibrate** | | |
| --- | --- | --- | --- | --- | --- | --- |
| **Parametric model** | **AIC** | **BIC** | **Average** | **AIC** | **BIC** | **Average** |
| Exponential | 122.80 | 124.33 | 123.56 | 51.41 | 53.04 | 52.22 |
| Weibull | 108.74 | 111.79 | 110.26 | 53.14 | 56.41 | 54.77 |
| Generalized gamma | NC | NC | - | 54.91 | 59.82 | 57.37 |
| Gompertz | 96.44 | 99.49 | 97.97 | 52.91 | 56.19 | 54.55 |
| Lognormal | 104.20 | 107.25 | 105.73 | 53.56 | 56.83 | 55.20 |
| Log Logistic | 105.95 | 109.01 | 107.48 | 53.40 | 56.68 | 55.04 |

AIC, Akaike information criteria; BIC, Bayesian information criteria; NC, non-convergence of model

Figure 19 Parametric survival curve fits for time from referable retinopathy to treatment for diabetic retinopathy in the placebo (standard care) arm

KM, Kaplan Meier; DR, diabetic retinopathy

Figure 20 Parametric survival curve fits for time from referable retinopathy to treatment for diabetic retinopathy in the fenofibrate arm

KM, Kaplan Meier; DR, diabetic retinopathy; Feno, fenofibrate

Figure 21 Selected curve fits for time from referable retinopathy (R3/R4) to treatment for diabetic retinopathy

KM, Kaplan Meier; DR, diabetic retinopathy; Feno, fenofibrate

### Referable maculopathy to referable diabetic retinopathy (R3/R4)

Figure 22 Log cumulative hazard plot for time from referable maculopathy to referable diabetic retinopathy

Table 11 Proportional hazards test for referable maculopathy to referable diabetic retinopathy by treatment allocation group

|  | Chi-squared | Degrees of freedom | P-value |
| --- | --- | --- | --- |
| Global test | 1.88 | 1 | 0.170 |

Table 12 Akaike and Bayesian information criteria of different parametric models of time from referable maculopathy to referable diabetic retinopathy (R3/R4)

| **Parametric model** | **AIC** | **BIC** | **Average** |
| --- | --- | --- | --- |
| Exponential | 192.83 | 199.85 | 196.34 |
| Weibull | 193.31 | 203.85 | 198.58 |
| Generalized gamma | 190.38 | 204.44 | 197.41 |
| Gompertz | 194.77 | 205.31 | 200.04 |
| Lognormal | 190.33 | 200.87 | 195.60 |
| Log Logistic | 192.60 | 203.14 | 197.87 |

AIC, Akaike information criteria; BIC, Bayesian information criteria

Figure 23 Parametric survival curve fits for time from referable maculopathy to referable diabetic retinopathy in the placebo (standard care) arm

KM, Kaplan Meier; DR, diabetic retinopathy

Figure 24 Parametric survival curve fits for time from referable maculopathy to referable diabetic retinopathy in the fenofibrate arm

KM, Kaplan Meier; DR, diabetic retinopathy, Feno, fenofibrate

Figure 25 Parametric survival curve fits for time from referable maculopathy to referable diabetic retinopathy

KM, Kaplan Meier; DR, diabetic retinopathy; Feno, fenofibrate

### Referable diabetic retinopathy (R3/R4) to referable maculopathy

Figure 26 Log cumulative hazard plot for time from referable retinopathy (R3/R4) to referable diabetic maculopathy

Table 13 Proportional hazards test for time from referable retinopathy (R3/R4) to referable diabetic maculopathy, with respect to treatment allocation group

|  | Chi-squared | Degrees of freedom | P-value |
| --- | --- | --- | --- |
| Global test | 2.44 | 1 | 0.118 |

Table 14 Akaike and Bayesian information criteria of different parametric models of time from referable diabetic retinopathy R3/R4) to referable maculopathy

| **Parametric model** | **AIC** | **BIC** | **Average** |
| --- | --- | --- | --- |
| Exponential | 35.15 | 38.37 | 36.76 |
| Weibull | 37.12 | 41.95 | 39.54 |
| Generalized gamma |  |  |  |
| Gompertz | 36.97 | 41.80 | 39.38 |
| Lognormal | 36.31 | 41.14 | 38.72 |
| Log Logistic | 192.60 | 203.14 | 197.87 |

AIC, Akaike information criteria; BIC, Bayesian information criteria

Figure 27 Parametric survival curve fits for time from referable retinopathy to referable maculopathy in the placebo (standard care) arm

KM, Kaplan Meier; Ref, referable

Figure 28 Parametric survival curve fits for time from referable retinopathy to referable maculopathy in the fenofibrate arm

KM, Kaplan Meier; Ref, referable; Feno, fenofibrate

Figure 29 Selected Parametric survival curve fits for time from referable retinopathy to referable maculopathy

KM, Kaplan Meier; Ref, referable; Feno, fenofibrate

## Health state utility impact of progression

Analysis of the EQ-5D data from LENS indicated negligible effects of treatment allocation on HRQoL over the trial follow-up period.^7^ This may be due to the relatively small absolute differences in number with progression over follow-up. Nevertheless, it is logical to assume that progression of retinopathy to sight threatening stages has a negative impact on HRQoL due to anxiety and the additional monitoring and treatment burden of developing referable disease. Therefore, the LENS trial data were pooled across the treatment arms, and mixed models for repeated measures were used to assess the effect of progression on health state utility. Alternative models were assessed with a random effect for individual, and fixed effects for the various types of progression event, time from baseline, baseline EQ-5D score, and other selected baseline covariates. Based on Akaike and Bayesian information criteria (AIC and BIC), the preferred model included fixed effects for baseline EQ-5D, baseline age, time from baseline, and any referable diabetic eye disease or any treatment thereof. EQ-5D data were available for n=1,142 (99%) at baseline, n=908 (79%) at two years, and n=854 (74% and study exit). There was n=346 (20%) post-progression observations during follow-up. The mixed model for repeated measures assumes data are missing at random, conditional on the covariates included in the analysis model.

Table 15 Available EQ-5D data by time point

| **Time point** | **Number (%)** | **Mean (SD)** |
| --- | --- | --- |
| Baseline | 1142 (99%) | 0.811 (0.226) |
| Two years | 908 (79%) | 0.758 (0.261) |
| Study exit | 854 (74%) | 0.745 (0.263) |

## Modelled long-term changes in visual acuity and impact on quality of life

With relatively few patients in the LENS trial reaching the stage where treatment was indicated for PDR or DMO, there was insufficient data to inform expected visual acuity outcomes following treatment. Therefore, external literature was consulted for this purpose, with a focus on pragmatic trials of diabetic retinopathy treatments and NHS cohort studies. Expected changes to VA following treatment initiation, were applied to observed best corrected visual acuities at the latest available assessment prior to treatment with anti-VEGF or panretinal laser photocoagulation (PRP). Based on findings from a real-world NHS cohort study, ant-VEGF injections were assumed to maintain VA at pre-treatment levels to two years post-treatment.^4^ Beyond two years, it was assumed that VA in treated eyes deteriorates slowly at the level observed during the long-term follow-up (under routine management) of patients enrolled in the Protocol T extension study.^8^ This reported a mean reduction of 4.7 ETDRS letters (1.6 ETDRS letters per year) between two years and five years of follow-up.

For eyes with proliferative diabetic retinopathy treated with PRP, VA changes were modelled to align with those reported in a retrospective NHS cohort study.^9^ Fu et al. studied 2,336 eyes with stable proliferative diabetic retinopathy following PRP and reported a 50% probability of losing ≥5 ETDRS letters or more by 3.32 years. A mean (SD) reduction of 14.2 (13) ETDRS letters was observed at this event. A six-monthly probability of losing ≥5 letters following PRP was estimated from this data and applied in the model, with the number of letters lost drawn from a gamma distribution with mean (SD) matching that reported by Fu et al.

Given uncertainty regarding the linkage of treatment to the individual eyes, it was conservatively assumed that post-treatment VA changes apply only to the WSE at treatment initiation. Sensitivity analysis assessed the impact of applying VA changes to the BSE or both eyes.

With relatively few patients in the LENS trial reaching more advanced stages of referable disease requiring treatment, the estimated utility decrement associated with referable disease (estimated from LENS trial EQ-5D data) was assumed to capture the effect on quality of life of early referable disease (prior to treatment). Longer-term utility impacts following initiation of retinal treatment were therefore modelled through expected impacts on visual acuity linked with external literature on health state utility by visual acuity status. Focused searches were used to identify studies reporting on the relationship between visual acuity and health state utility values in people with diabetes. The focus was on identifying studies of patient reported health status using health related quality of life instruments for which health state utility weights, suitable for the calculation of QALYS, are available. The selected source reported EQ-5D and VFQ-25 derived values based on application of the UK general population valuation tariff to EQ-5D-3L response data, and the VFQ-UI to VFQ-25 response data, collected from 1,320 patients enrolled in four trials of intraretinal aflibercept injections for macular oedema.^10^ Brazier et al. reported predicted EQ-5D and VFQ-UI values based on regression models accounting for BCVA in the best and worst seeing eye over time. The estimated effects from the authors preferred ordinary least squares model of EQ-5D values was chosen for incorporation in the model base case. This can be considered conservative, as there are recognised limitations of the EQ-5D for picking up changes in quality of life due to changes in VA. The VFQ-UI can be considered a more sensitive measure for this purpose, which was tested in sensitivity analysis.

## Modelled resource use assumptions

Six-monthly background health care resource use costs of LENS trial participants, inclusive of biochemistry monitoring, hospital activity in specialties of interest (excluding ophthalmology and diabetic retinopathy treatment costs), and relevant community prescribed medicines, were analysed by treatment allocation group and health state occupancy. General estimating equations (GEEs) were used to estimate differences in these costs by health state and/or treatment allocation. Whilst the estimated mean difference in these six-monthly health care costs directionally favoured fenofibrate, the difference was not statistically significant (-113; 95% CI: -275 to 49). Similarly, no statistically significant differences in background health care costs were identified by diabetic retinopathy progression status. Therefore, they were conservatively equalised across treatment arms and health states in the economic model and assumed not to influence modelled cost-effectiveness.

A similar analysis was performed to estimate screening costs by model health state, with the GEE regression including health state indicators for referable maculopathy, treated maculopathy, referable retinopathy, and treated retinopathy. Screening costs were assumed to stop for those developing referable retinopathy (R3/R4) or macular oedema requiring treatment, since care and follow-up would move to hospital eye services for these patients. Cost of prescribing fenofibrate were assumed to be negligible and absorbed in the context of ongoing diabetes management and monitoring. Sensitivity analysis assessed the impact of including an extra GP appointment to initiate treatment and an additional creatinine test at 1-2 months to assess kidney function following initiation of treatment.

Those being referred for treatment of DMO were assumed to require 6.3 injections in year 1 and 2.9 injections in year two, based on data from a contemporary NHS cohort study under routine practive.^9^ In years 3-5, they were assumed to continue undergoing six-monthly outpatient monitoring in the hospital eye service and receive a further 2.45 injections over this time-period. This was based on the ratio of the median number of aflibercept injections in years 3-5 to the median number in years 1-2 of the Protocol T study (=4/15),^118^ multiplied by the mean number of injections over two years in the DRAKO cohort study (9.2).^9^ No further treatment was assumed after five years, but patients continued to be monitored annually in the hospital outpatient eye service based on clinical expert opinion.

Modelled resource use assumptions for retinal treatment were aligned with the sources of evidence used to inform expected post-treatment changes in visual acuity and were validated by clinical expert opinion. Those referred for severe background retinopathy (R3) were assumed to require six-monthly monitoring via face-to-face ophthalmology outpatient appointments. Patients progressing to PRP for proliferative DR were assumed to require on average of 2.77 treatment sessions (applied in the cycle of treatment initiation),^12^ followed by six-monthly outpatient monitoring in hospital eye services. Beyond three years, it was assumed that patients would be discharged from the hospital eye services back to the screening programme.

## Microsimulation model analysis

Monte Carlo simulation was used to propagate the passage if simulated patients through the model one at time. Simulated patients were generated by bootstrap resampling of the baseline characteristics of LENS trial participants, retaining correlations between characteristics: Age group (<30; ≥30 <50; ≥50 <70; ≥70 years), sex, type of diabetes (type 1, type 2, other), HbA1c category (<64; ≥64mmol/mol; unknown), estimated glomerular filtration rate category (<60; ≥60 mL/min/1.73m2), statin use, baseline maculopathy grade (none, observable), baseline retinopathy grade (none, mild, observable). These characteristics were combined with the parameter estimates of the fitted survival regressions for the components of the first documented progression (referable maculopathy or treatment for diabetic macular oedema; referable retinopathy (R3/R4) or treatment thereof), to generate individualised transition probabilities for these events. Following initial progression, transitions followed average estimates by treatment allocation group, due to their being too few events to inform transitions by individual characteristics. Assessment of modelled costs and outcomes over the primary ten-year time horizon determined that incremental costs stabilised (+/-£4) and incremental QALYs stabilised within +/- 0.001 across 10 batches of 500,000 simulations. With respect to probabilistic sensitivity analysis, a pragmatic compromise was made to use 1,000 draws to represent second order uncertainty in the parameter estimates. However, the computational burden was too great to enable 1,000 x 500,000 runs, and so 1,000 x 50,000 was used. Whilst point estimates of the ICER fluctuated somewhat based on these numbers, the decision uncertainty (probabilities of cost-effectiveness at key thresholds) was found to be stable.

Heterogeneity in the cost-effectiveness was explored for key subgroups with enough participants to accurately inform subgroup specific progression risks: type of diabetes, baseline HbA1c status; and baseline maculopathy grade. Whilst these analyses reflect subgroup specific risks of progression, they assume the same overall effects of fenofibrate on progression risks. This is supported by evidence from the clinical effectiveness analysis, which supported a consistent treatment effect across all prespecified subgroups.^7^

## Model validation

Consistency and validity were assessed by careful checking of formulas, running scenarios to equate costs and QALYs between treatment arms, checking that changes in model parameter inputs produced results in line with expectation, and by assessing the fit of modelled clinical progression outcomes against the summary Kapan-Meier curves from the LENS trial. These projections (modelled outcomes), provided as supplementary figures 1 and 2, showed a good fit with the observed data and make clear the ten-year extrapolation of outcomes. Recognising the uncertainty inherent in any extrapolation beyond observed follow-up data, we also assessed the impact of more conservative assumptions, including no further relative treatment effects beyond five years. Further, the model inputs and assumptions with respect to post-progression monitoring and treatment resource use, were carefully informed through consultation with clinical experts and review of contemporary NHS based cohort studies.

**References**

1. Public Health Scotland. Scottish health service costs 22/23. <https://publichealthscotland.scot/publications/scottish-health-service-costs/scottish-health-service-costs-summary-for-financial-year-2022-to-2023/.> Updated 2023. Accessed 03/03, 2023.

2. Jones, Karen C., Burns, Amanda. Unit costs of health and social care 2022. *Personal Social Services Research Unit*. 2023.

3. NHS England. National cost collection for the NHS 21/22. <https://www.england.nhs.uk/publication/2021-22-national-cost-collection-data-publication/.> Accessed 03/03, 2023.

4. Sivaprasad S, Ghanchi F, Kelly SP, et al. Evaluation of care with intravitreal aflibercept treatment for UK patients with diabetic macular oedema: DRAKO study 24-month real-world outcomes. *Eye (Lond)*. 2023;37(13):2753–2760. doi: 10.1038/s41433-023-02409-y.

5. NICE. British national formulary (BNF). <https://bnf.nice.org.uk/> Web site. <https://bnf.nice.org.uk/.> Accessed 03/03, 2023.

6. Keech AC, Mitchell P, Summanen PA, et al. Effect of fenofibrate on the need for laser treatment for diabetic retinopathy (FIELD study): A randomised controlled trial. *Lancet*. 2007;370(9600):1687–1697. doi: 10.1016/S0140-6736(07)61607-9.

7. Preiss D, Logue J, Sammons E, et al. Effect of fenofibrate on progression of diabetic retinopathy. *NEJM Evid*. 2024;3(8):EVIDoa2400179. doi: 10.1056/EVIDoa2400179.

8. Glassman AR, Wells JA3, Josic K, et al. Five-year outcomes after initial aflibercept, bevacizumab, or ranibizumab treatment for diabetic macular edema (protocol T extension study). *Ophthalmology*. 2020;127(9):1201–1210. doi: 10.1016/j.ophtha.2020.03.021.

9. Fu DJ, Thottarath S, Faes L, et al. Visual acuity outcome of stable proliferative diabetic retinopathy following initial complete panretinal photocoagulation. *BMJ Open Ophth*. 2022;7(1):e001068. <http://bmjophth.bmj.com/content/7/1/e001068.abstract>. doi: 10.1136/bmjophth-2022-001068.

10. Brazier J, Muston D, Konwea H, et al. Evaluating the relationship between visual acuity and utilities in patients with diabetic macular edema enrolled in intravitreal aflibercept studies. *Invest Ophthalmol Vis Sci*. 2017;58(11):4818–4825. doi: 10.1167/iovs.17-21945.

11. Wells JA, Glassman AR, Ayala AR, et al. Aflibercept, bevacizumab, or ranibizumab for diabetic macular edema: Two-year results from a comparative effectiveness randomized clinical trial. *Ophthalmology*. 2016;123(6):1351–1359. doi: 10.1016/j.ophtha.2016.02.022.

12. Sivaprasad S, Hykin P, Prevost AT, et al. Intravitreal aflibercept compared with panretinal photocoagulation for proliferative diabetic retinopathy: The CLARITY non-inferiority RCT. *EFFICACY AND MECHANISM EVALUATION*. 2018;5(5):1–89. doi: 10.3310/eme05050.
