# Supplementary material for: Cost‐effectiveness of fenofibrate versus standard care for reducing the progression of diabetic retinopathy: An economic evaluation based on data from the LENS trial
Source: Diabet Med. 2025 Jul 3;42(9):e70098. doi: 10.1111/dme.70098 (PMC7617897; doi:10.1111/dme.70098)
Supplement: Supplementary file 2 — Data S2. [file DME-42-e70098-s001.pdf]

Supplementary Figure 1. Comparison of model output against observed Kaplan-Meier based estimates of time to any referable diabetic retinopathy

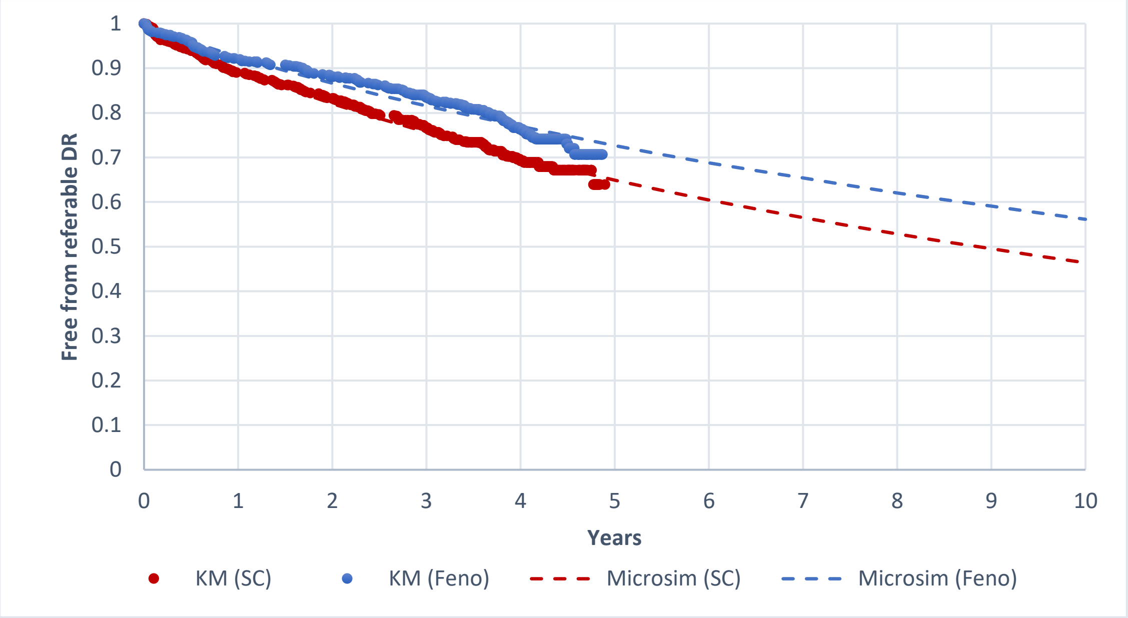

Notes: DR, diabetic retinopathy; KM, Kaplan-Meier; Microsim, Microsimulation model; SC, standard care; Feno, Fenofibrate.

Supplementary Figure 2. Comparison of model output against observed Kaplan-Meier based estimates of time to any treatment for diabetic retinopathy

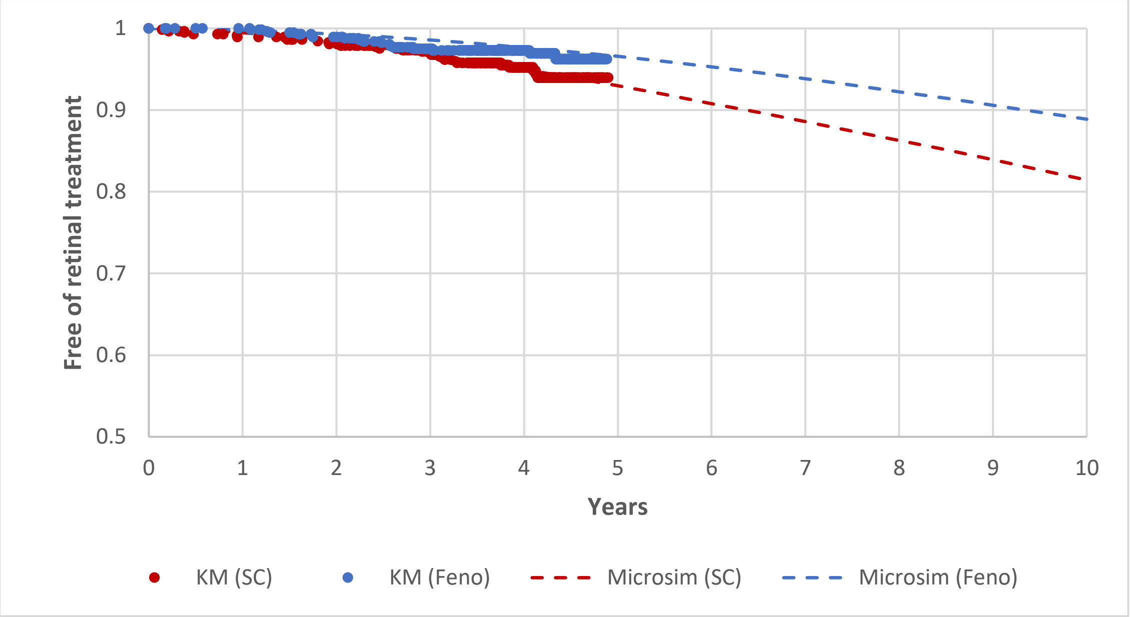

Notes: DR, diabetic retinopathy; KM, Kaplan-Meier; Microsim, Microsimulation model; SC, standard care; Feno, Fenofibrate.

Supplementary Figure 3 Cost-effectiveness acceptability curves for fenofibrate, showing the impact of price discounts on anti-VEGF drugs (aflibercept) used to treat diabetic macular oedema.

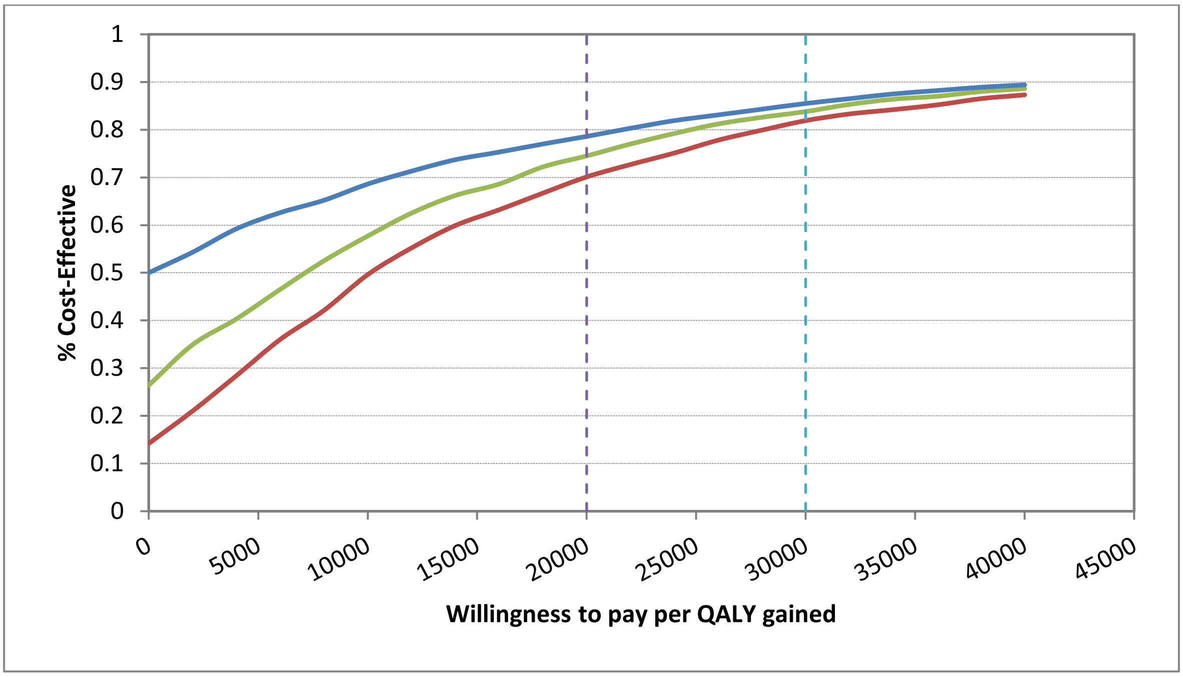

Notes: Blue, no discount on price of antiVEGF drugs; Red, 70% discount on antiVEGF drugs; Green, 70% discount on antiVEGF drugs but including bilateral treatment.
