# Supplementary material for: Cost‐effectiveness of fenofibrate versus standard care for reducing the progression of diabetic retinopathy: An economic evaluation based on data from the LENS trial
Source: Diabet Med. 2025 Jul 3;42(9):e70098. doi: 10.1111/dme.70098 (PMC7617897; doi:10.1111/dme.70098)
Supplement: Supplementary file 3 — Data S3. [file DME-42-e70098-s002.docx]

**Supplementary Tables**

**Supplementary Table 1. NHS Scotland’s Diabetic Eye Screening Programme grading scheme**

| **Grade** | **Description** | **Findings** | **Outcome** |
| --- | --- | --- | --- |
| **RETINOPATHY (excluding the macula)** | | | |
| R0 | No DR anywhere | - | Rescreen in 12-24 months |
| R1 | Mild background diabetic retinopathy | The presence of at least one of any of the following features anywhere:   - dot haemorrhages / microaneurysms - hard exudates - cotton wool spots - blot haemorrhages* - superficial/ flame shaped haemorrhages | Rescreen in 12 months |
| R2 | Observable background diabetic retinopathy | Four or more blot haemorrhages* in one hemi-field only (Inferior and superior hemi-fields delineated by a line passing through the centre of the fovea and optic disc) | Rescreen in 6 months |
| R3 | Referable background diabetic retinopathy | Any of the following features:   - four or more blot haemorrhages* in both inferior and superior hemi-fields - Venous beading - Intraretinal Microvascular Abnormalities (IRMA) | Specialist referral (routine) |
| R4 | Proliferative diabetic retinopathy | Any of the following features:   - Active new vessels - Vitreous haemorrhage | Specialist referral (urgent) |
| R4i^†^ | Treated proliferative diabetic retinopathy | Any of the following features:   - Inactive new vessels with evidence of laser treatment | Rescreen in 12 months |
| R6 | Not adequately visualised | Retina not sufficiently visible for assessment | Technical failure |
| **MACULOPATHY** | | | |
| M0 | No maculopathy | No features ≤2 disc diameters from the centre of the fovea sufficient to qualify for M1 or M2 | Rescreen in 12-24 months |
| M1 | Observable maculopathy | Lesions as specified below within a radius of >1 but ≤2 disc diameters from the centre of the fovea:   - Any hard exudates | Rescreen in 12 months |
| M2^‡^ | Referable maculopathy | Lesions as specified below within a radius of ≤1 disc diameter of the centre of the fovea:   - Any blot haemorrhages* - Any hard exudates | OCT surveillance scan or Rescreen in 12 months |
| * Blot haemorrhage has the same or greater diameter as a retinal vein crossing the optic disc  ^†^ R4i is not counted towards the primary outcome as it represents inactive disease  ^‡^ At the start of LENS in 2018 and until end-2021, all patients in Scotland with M2 retinal screening results required referral to a specialist in ophthalmology. During 2022, the Diabetic Eye Screening programme started to introduce a phased change to the management pathway of patients with M2 disease. In the new pathway, M2 in the context of poor visual acuity (e.g. 6/9.5 or worse) leads to optical coherence tomography (OCT) imaging followed by referral to an ophthalmologist if there is evidence of macular oedema. | | | |

**Supplementary Table 2 Unit costs and sources**

| **Resource item** | **How measured** | **Source of measurement** | **Unit cost*** | **Source of valuation** |
| --- | --- | --- | --- | --- |
| Study medication | Prescribed quantity and dose | Study database | £4.77 (Scottish Tariff)  2.69 (English drug tariff)  28 pack (200mg capsules) | Scottish and English Drug Tariff ^12 13^ |
| Biochemistry tests | Number of tests | Study database, linked from SCI-Diabetes | £0.90 per test | Scottish Health Service Costs ^14^ |
| GP time | Time associated with request and review of biochemistry tests | Assumption (5 minutes) | £178 per hour | Unit costs of Health and Social Care ^11^ |
| Practice nurse time | Time to obtain sample | Assumption (5 minutes) | £53 per hour | Unit costs of Health and Social Care ^11^ |
| Hospital admissions | Number of episodes, specialty, length of stay. | Linked SMR01 data | Variable by specialty | Scottish Health Service costs ^14^ |
| Hospital outpatient attendances | Number of episodes, specialty | Linked SMR00 data | Variable by specialty | Scottish Health Service costs ^14^ |
| Retinal Screening episodes | Number of episodes | DES | £44.47 | Scotland et al. 2016 ^16^ |
| OCT monitoring of maculopathy (M2) | Number of episodes | DES | £41.03 | Scotland et al. 2016 ^16^ |
| Slit lamp examination (screening programme) | Number of episodes | DES | £36.14 | Olson et al. 2013 ^15^ |
| Prescribed medicines | Medicine name, dose, quantity | Prescribing Information System | Variable | BNF ^12^ |
| **DR related interventions** | | | | |
| Ophthalmology outpatient visit | Number of visits | DES and SCI diabetes systems; trial specific questionnaires; supporting data from local NHS health records provided by LCCs); SMR01; and SMR00. | £232 (Scottish average)  £154 (English average) inflated from 144 in 2021/2022 prices | Scottish Health Service Costs, NHS Reference costs ^14^ |
| Pan-retinal laser photocoagulation session | Assumptions regarding expected number of sessions (see text) |  | £164 inflated from 153 in 2021/2022 prices | NHS Reference costs (BZ86) ^27^ |
| Administration of anti-VEGF injection | Assumptions regarding expected number of injections (see text) |  | £164 inflated from 153 in 2021/2022 prices | NHS Reference costs (BZ86) ^27^ |
| Vitrectomy | Number of events |  | £2,573 inflated from 2,404 in 2021/2022 prices | NHS Reference costs (BZ83) ^27^ |
| Anti-VEGF drugs | Number of administrations |  | £551 - Ranibizumab (Lucentis); £816 - Aflibercept (Eylea) | BNF (list prices) ^12^ |

OCT, optical coherence tomography; LCC, local coordinating centres; VEGF, vascular endothelial growth factor; BNF, British National Formulary; *Prices reflect 2022/2023 cost year

**Supplementary Table 3 Summary of resource use by treatment allocation group full follow-up period**

| **Resource use category** | **Fenofibrate (N=576)** | **Placebo**  **(N=575)** | **All participants (N=1151)** |
| --- | --- | --- | --- |
| Screening episodes, (mean (SD) | 2.66 (1.11) | 2.69 (1.13) | 2.67 (1.12) |
| Digital retinal screening | 2.48 (1.06) | 2.41 (1.09) | 2.44 (1.07) |
| OCT | 0.08 (0.42) | 0.13 (0.58) | 0.11 (0.51) |
| Slit lamp | 0.11 (0.46) | 0.14 (0.51) | 0.12 (0.49) |
| Biochemistry tests, mean (SD) | 26.14 (13.50) | 25.51 (12.74) | 25.82 (13.12) |
| Albumin /Creatinine | 2.44 (2.08) | 2.37 (1.98) | 2.40 (2.03) |
| HDL cholesterol | 3.45 (2.06) | 3.35 (2.04) | 3.40 (2.05) |
| HbA1c | 5.65 (2.85) | 5.72 (2.84) | 5.69 (2.84) |
| Triglycerides | 2.65 (2.29) | 2.54 (2.27) | 2.60 (2.28) |
| Creatinine | 8.16 (7.02) | 7.77 (6.31) | 7.96 (6.68) |
| Total cholesterol (mmol/L) | 3.79 (2.09) | 3.76 (2.01) | 3.78 (2.05) |
| Prescribing episodes (episodes), mean (SD) | 129.38 (71.17) | 128.95 (71.08) | 129.17 (71.09) |
| Blood pressure | 32.33 (29.87) | 30.82 (29.03) | 31.58 (29.45) |
| Diabetes | 67.91 (38.46) | 67.81 (38.07) | 67.86 (38.25) |
| Other | 8.23 (17.17) | 8.72 (16.39) | 8.48 (16.78) |
| Oedema | 1.45 (5.95) | 1.82 (6.49) | 1.64 (6.22) |
| Statins | 19.45 (13.07) | 19.78 (12.55) | 19.62 (12.81) |
| Outpatient visits, n (%) | 477 (82.8%) | 471 (81.9%) | 948 (82.4%) |
| Specialties of interest | 351 (60.9%) | 350 (60.9%) | 701 (60.9%) |
| Ophthalmology | 151 (26.2%) | 152 (26.4%) | 303 (26.3%) |
| Others | 369 (64.1%) | 342 (59.5%) | 711 (61.8%) |
| Outpatient visits, mean (SD) | 8.33 (11.09) | 9.04 (12.27) | 8.68 (11.69) |
| Specialties of interest | 3.27 (4.70) | 3.73 (5.31) | 3.50 (5.01) |
| Ophthalmology | 0.97 (2.78) | 1.01 (2.78) | 0.99 (2.78) |
| Others | 4.09 (8.12) | 4.30 (9.10) | 4.19 (8.62) |
| Inpatient/day case admissions, n (%) | 247 (42.9%) | 247 (43.0%) | 494 (42.9%) |
| Specialties of interest | 175 (30.4%) | 162 (28.2%) | 337 (29.3%) |
| Others | 142 (24.7%) | 143 (24.9%) | 285 (24.8%) |
| Inpatient/day case (episodes), mean (SD) | 1.85 (4.43) | 1.96 (3.80) | 1.90 (4.13) |
| Specialties of interest | 1.00 (2.08) | 1.24 (2.97) | 1.12 (2.57) |
| Others | 0.85 (3.50) | 0.72 (1.92) | 0.79 (2.82) |
| SD: standard deviation, OCT: optical coherence tomography, HDL: high-density lipoprotein | | | |

**Supplementary Table 4 Summary of health service costs by treatment group full follow-up period**

| Resource use category | Fenofibrate (N=576) (£) | Placebo  (N=575) (£) | All participants (N=1151) (£) |
| --- | --- | --- | --- |
| Intervention acquisition cost | 221(86) | 0 | N/A |
| Screening episodes, (mean (SD) | 117 (49) | 118 (49) | 117 (49) |
| Digital retinal screening | 110 (47) | 107 (48) | 109 (48) |
| OCT | 3 (17) | 5 (23) | 4 (21) |
| Slit lamp | 4 (17) | 5 (18) | 4 (18) |
| Biochemistry tests, mean (SD) | 216 (158) | 210 (143) | 213 (150) |
| Albumin /Creatinine | 2 (2) | 2 (2) | 2 (2) |
| HDL cholesterol | 3 (2) | 3 (2) | 3 (2) |
| HbA1c | 5 (3) | 5 (3) | 5 (3) |
| Triglycerides | 2 (2) | 2 (2) | 2 (2) |
| Creatinine | 7 (6) | 7 (6) | 7 (6) |
| Total cholesterol | 3 (2) | 3 (2) | 3 (2) |
| GP and nurse time | 194 (147) | 188 (133) | 191 (140) |
| Prescribing episodes (episodes), mean (SD) | 3,132 (2,199) | 3,116 (2,220) | 3,124 (2,209) |
| Blood pressure | 102 (248) | 111 (287) | 107 (268) |
| Diabetes | 2,835 (2,126) | 2,810 (2,117) | 2,823 (2,121) |
| Other | 155 (490) | 151 (454) | 153 (472) |
| Oedema | 2 (10) | 3 (12) | 3 (11) |
| Statins | 37 (23) | 40 (65) | 39 (49) |
| Outpatient visits, mean (SD) | 2,179 (4,171) | 2,226 (3,001) | 2,202 (3,633) |
| Specialties of interest | 1,051 (1,484) | 1,167 (1,553) | 1,109 (1,520) |
| Ophthalmology | 226 (646) | 235 (644) | 230 (645) |
| Others | 1,127 (3,341) | 1,059 (2,222) | 1,093 (2,837) |
| Anti-VEGF drug cost | 107(855) | 127(904) | 117(879) |
| Inpatient/day case (episodes), mean (SD) | 5,207 (13,170) | 6,135 (20,803) | 5,671 (17,405) |
| Specialties of interest | 2,475 (8,128) | 3,198 (17,819) | 2,836 (13,844) |
| Others | 2,731 (9,144) | 2,937 (8,407) | 2,834 (8,780) |
| Inpatients/day case (continuous inpatient stays), mean (SD) | 5,207 (13,170) | 6,135 (20,803) | 5,671 (17,405) |
| Specialties of interest | 3,073 (10,034) | 3,987 (18,952) | 3,529 (15,160) |
| Others | 2,134 (7,965) | 2,149 (7,033) | 2,141 (7,511) |
| SD: standard deviation, OCT: optical coherence tomography, HDL: high-density lipoprotein | | | |

OCT: Optical Coherence Tomography

**Supplementary Table 5 Trial based cost-effectiveness scenario analysis results at two years (fenofibrate versus standard care)**

| **Population/Subgroups** | **Base case** | **Scenario** | **Incremental cost (£)** | **Incremental QALYs** | **ICER (£)** |
| --- | --- | --- | --- | --- | --- |
| **Base case** | | | -254 (-1062 to 624) | 0.044 (0.013 to 0.08) | Dominant |
| Price discount on anti-VEGF treatment | List price | 30% | -249 (-1062 to 627) | 0.044 (0.013 to 0.08) | Dominant |
|  |  | 70% | -243 (-1048 to 642) | 0.044 (0.013 to 0.08) | Dominant |
| Hospital costs | Prior specified specialties of interest | All specialties | -741(-1949 to 425) | 0.044 (0.013 to 0.08) | Dominant |

**Supplementary Table 6 Within trial comparison of six-month interval-based costs by treatment group**

|  | Fenofibrate | | | Placebo | | |  |
| --- | --- | --- | --- | --- | --- | --- | --- |
| Six monthly intervals | N | Mean cost (£)* | SE | N | Mean cost (£)* | SE | Difference between groups (95% CI) |
| 1 | 576 | 690 | 101 | 575 | 808 | 118 | -118 (-421, 186) |
| 2 | 573 | 837 | 122 | 573 | 1,317 | 193 | -481 (-927, -34) |
| 3 | 570 | 672 | 99 | 571 | 734 | 108 | -62 (-348, 223) |
| 4 | 566 | 973 | 143 | 568 | 896 | 132 | 77 (-304, 457) |
| 5 | 560 | 730 | 108 | 564 | 961 | 142 | -230 (-579, 118) |
| 6 | 549 | 981 | 147 | 549 | 875 | 131 | 106 (-278, 491) |
| 7 | 509 | 930 | 144 | 513 | 1,080 | 167 | -151 (-582, 281) |
| 8 | 467 | 961 | 160 | 459 | 746 | 125 | 214 (-173, 602) |
| 9 | 298 | 761 | 166 | 296 | 1,125 | 246 | -364 (-907, 178) |
| 10 | 103 | 2,003 | 748 | 105 | 1,445 | 538 | 558 (-1104, 2221) |
|  |  |  |  |  |  |  |  |
| Average over trial follow-up | 576 | 844 | 49 | 575 | 945 | 55 | -101 (-243, 42) |
| *Adjusted for minimisation covariates which included categories of age, type of diabetes, sex, HbA1c, renal function, statin use, baseline retinopathy grade, and baseline maculopathy grade.  SE, standard error; N, number; CI, confidence interval | | | | | | | |

**Supplementary Table 7 Model input parameters**

| **Parameters** | **Value** | **SE or (95% CI)** | **Distributional form** | **Source** |
| --- | --- | --- | --- | --- |
| Settings | | | | LENS trial |
| Start age | 61 (12.4) |  | 1^st^ order variation |  |
| Proportion Male | 0.73 |  | 1^st^ order variation |  |
| Cycle length years | 0.5 |  |  |  |
| Cycle length days | 182.63 |  |  |  |
| Discount rate (costs) | 0.035 |  |  |  |
| Discount rate (QALYs) | 0.035 |  |  |  |
| Clinical efficacy inputs (hazards expressed per 6-month model cycle) | | | |  |
| Non referable to referable DR, with or without concurrent referable maculopathy (exponential function) | | | | LENS trial |
| Hazard rate | 0.006 | 0.004 – 0.009 | Multivariate normal (log scale) |  |
| Hazard ratio (Feno)* | 1.309 | 0.746 – 2.295 |  |  |
| Non referable to referable maculopathy, alone (Weibull function) | | | | LENS trial |
| Scale | 0.052 | 0.041 – 0.068 | Multivariate normal (log scale) |  |
| Shape | 0.857 | 0.761 – 0.965 |  |  |
| Hazard ratio (Feno)* | 0.661 | 0.513 – 0.851 |  |  |
| Hazard ratio for the primary composite outcome (from parametric Weibull regression) | | | | LENS trial |
| Hazard ratio (Feno)^+^ | 0.73 | (0.58 – 0.92) | Lognormal |  |
| Referable maculopathy to referable DR (exponential function) | | | | LENS trial |
| Hazard rate | 0.020 | 0.012 – 0.035 | Multivariate normal (log scale) |  |
| Hazard ratio (Feno) | 1.457 | 0.665 – 3.194 |  |  |
| Referable DR to referable maculopathy (exponential function) | | | | LENS trial |
| Hazard rate | 0.036 | 0.009 – 0.144 | Multivariate normal (log scale) |  |
| Hazard ratio (Feno) | 0.740 | 0.104 – 5.254 |  |  |
| Referable maculopathy to treatment for DMO (exponential function) | | | | LENS trial |
| Hazard rate | 0.016 | 0.009 – 0.030 | Multivariate normal (log scale) |  |
| Hazard ratio (Feno) | 0.682 | 0.237 – 1.963 |  |  |
| Referable DR to treatment for referable DR with standard care (log normal distribution) | | | | LENS trial |
| Constant | 0.679 | -0.425 – 1.784 | Multivariate normal (log scale) |  |
| lnSigma | 0.951 | 0.560 – 1.341 |  |  |
| Referable DR to treatment for referable DR with fenofibrate (log normal distribution) | | | | LENS trial |
| Constant | 2.345 | 1.291 – 3.399 | Multivariate normal (log scale) |  |
| lnSigma | 0.445 | -0.076 – 0.967 |  |  |
| Other clinical inputs | | | |  |
| Hazard for fenofibrate discontinuation | 0.034 | 0.003 | Normal | LENS trial |
| Probability of death | Age and sex specific UK life tables |  | NA | ONS, 2024 |
| Standardised mortality ratio (Type 1 diabetes, male) | 2.95 | 2.74 – 3.18 | Log normal | O'Reilly et al.^20^ |
| Standardised mortality ratio (Type 1 diabetes, female) | 4.62 | 4.20 – 5.08 | Log normal | O'Reilly et al. ^20^ |
| Standardised mortality ratio (Type 2 diabetes, male) | 1.36 | 1.28 – 1.45 | Log normal | Collier et al.^19^ |
| Standardised mortality ratio (Type 2 diabetes, female) | 1.48 | 1.38 – 1.59 | Log normal | Collier et al.^19^ |
| Proportion RDR treated patients receiving PRP | 1 |  | NA | LENS trial |
| Proportion PRP treated patients also receiving vitrectomy | 0.11 | 0.06 | Beta  (α=3; β=24) | LENS trial |
| Proportion of fenofibrate prescriptions that are once daily | 0.72 | 0.005 | Beta  (α=6392; β=2483) | LENS trial |
| Proportion of patients first developing RDR that have concurrent referable maculopathy at time of referral | 0.34 | 0.07 | Beta  (α=17; β=33) | LENS trial |
| Proportion of patients developing RDR that are treated immediately on progression | 0.04 | 0.02 | Beta (α=3; β=72) | LENS trial |
| Proportion of patients developing referable maculopathy that are treated immediately on progression | 0.015 | 0.007 | Beta (α=4; β=262) | LENS trial |
| Proportion of RDR treated patients requiring bilateral treatment* | 0.9 | 0.09  (assumed) | Beta | Expert opinion |
| Proportion of referable maculopathy treated patients requiring bilateral treatment* | 0.4 | 0.04  (assumed) | Beta | Expert opinion |
| Health state utility values | | | |  |
| Baseline utility | 0.811 (0.23) |  | 1^st^ order variable | LENS trial |
| Utility by health state and visual acuity status | | | |  |
| Constant | 0.142 | 0.037 | Multivariate normal |  |
| Time from baseline (days) | -0.00003 | 0.00001 |  |  |
| Post referable disease | -0.020 | 0.011 |  | LENS trial |
| Baseline age | -0.0013 | 0.0005 |  |  |
| Baseline EQ-5D | 0.885 | 0.024 |  |  |
| Utility coefficient for log ETDRS letters in BSE | 0.1 | 0.027 | Normal | Brazier et al.^23^ |
| Utility coefficient for log ETDRS letters in WSE | 0.042 | 0.014 | Normal | Brazier et al.^23^ |
| Resource use and costs | | | |  |
| Fenofibrate micronised 200mg capsules (price per pack) | £4.77 |  | NA | Scottish drug tariff^13^ |
| Fenofibrate micronised 200mg capsules (pack size) | 28 |  | NA |  |
| Fenofibrate 67mg micronised capsules (price per pack) | £23.23 |  | NA | Scottish drug tariff^13^ |
| Fenofibrate 67mg micronised capsules (pack size) | 90 |  |  |  |
| Cost of fenofibrate treatment per cycle (assuming 200mg every other day for those with reduced eGFR) | £26.76 |  | Calculated |  |
| Cost of fenofibrate treatment per cycle (assuming 67mg every day for those with reduced eGFR) | £47.14 |  | Calculated |  |
| Health state background HCRU costs | £864.10 | £39.02 | Normal | LENS trial |
| Average screening costs per six-month model cycle by cycle number and referable maculopathy status Log e linear predictors) | | | |  |
| Constant | 2.53 | 0.027 | Multivariate normal |  |
| Cycle number (six-monthly) | 0.02 | 0.005 |  | LENS trial |
| Referable maculopathy | 0.31 | 0.036 |  |  |
| Cost per ophthalmology outpatient appointment (Scotland) | £232 | 23.20 (assumed) | Normal | Scottish Health Service costs^14^ |
| Cost per ophthalmology outpatient appointment (England) | £133 |  | NA | NHS Reference costs^27^ |
| Cost per panretinal laser photocoagulation session (Scotland) | £232 | 23.20 (assumed) | Normal | Scottish Health Service costs^14^ |
| Cost per panretinal laser photocoagulation session (England) | £159 |  | NA | NHS Reference costs^27^ |
| Cost of vitrectomy (unilateral) | £2,573 | 257.30 (assumed) | Normal | NHS Reference costs^27^ |
| Cost of aflibercept (per injection) | £816 |  | NA | BNF^12^ |
| Cost of ranibizumab (per injection) | £551 |  | NA | BNF^12^ |
| Cost per administration of an anti-VEGF injection (Scotland) | £232 | 23.20 (assumed) | Normal | Scottish Health Service costs^14^ |
| Cost per administration of an anti-VEGF injection (England) | £159 |  | NA | NHS Reference costs^27^ |
| Number of anti-VEGF injections in year 1 of treatment for DMO | 6.3 | 0.13  (approximated) | Gamma | Sivaprasad et al. ^17^ |
| Number of anti-VEGF injections in year 2 of treatment for referable DMO | 2.9 | 0.115  (approximated) | Gamma | Sivaprasad et al.^17^ |
| Number of anti-VEGF injections years 3-5 | 2.45 | 0.25 | Gamma | Glassman et al.^22^ |
| Number of additional outpatient appointments for anti-VEGF treatment monitoring | 1.0 |  | NA | Assumption |
| Number of additional outpatient appointments for PRP treatment monitoring | 1.0 |  | NA | Assumption |
| Number of PRP sessions to achieve stable PDR (assumed over 12 weeks) | 2.77 | 0.015  (approximated) | Gamma | Sivaprasad et al.^26^ |
| ^+^Used in the base case for effect of fenofibrate on progression to RDR and RMO; *applied in scenario analysis  DR: diabetic retinopathy, MO: maculopathy, RDR: referable diabetic retinopathy, PRP: panretinal photocoagulation, VEGF: vascular endothelial growth factor, ETDRS: Early Treatment Diabetic Retinopathy Study, BSE: better seeing eye, WSE: worse seeing eye, eGFR: estimated glomerular filtration rate, HCRU: Health and Care Research Unit, PDR: proliferative diabetic retinopathy, Feno.: fenofibrate | | | |  |

**Supplementary Table 8 Model based cost-effectiveness scenario analyses and their justification**

| **Parameter / assumption** | **Base case** | **Scenario** | **Justification** |
| --- | --- | --- | --- |
| 1. Time horizon | 10 years | a) 5 years | The duration over which differences in cost and benefits are extrapolated will influence cost-effectiveness |
|  |  | b) 20 years |  |
|  |  | c) 30 years |  |
| 2. Time to referable DR and referable MO | Single overall treatment effect | Cause specific treatment effects | Fenofibrate may have different effects on progression of maculopathy and retinopathy |
| 3. Time to referable MO | Weibull curve | Exponential curve | Provided the next best fitting curves for time to referable maculopathy |
|  |  | Log logistic curve |  |
| 4. Time to referable retinopathy (R3/R4) | Exponential | Gompertz | Provided the next best fitting curves for time to referable retinopathy |
|  |  | Log logistic |  |
| 5. Treatment effects of fenofibrate | Continuous proportional hazards | Waned from 5 years | The duration of effects of fenofibrate beyond the trial follow-up are uncertain |
| 6. Effect of fenofibrate on post-progression transitions | Estimated from the data | Assume no effect | Ongoing effects of fenofibrate on progression, following first progression event, are uncertain |
| 7. Time from referable retinopathy (R3/R4) to treatment for referable retinopathy | Lognormal (both arms) | Exponential (both arms) | Alternative plausible extrapolations of time from referable DR to treatment, avoiding curves crossing before ten years |
|  |  | Weibull (both arms) |  |
|  |  | Lognormal (SC), exponential (feno) |  |
|  |  | Exponential (SC), Gompertz (feno) |  |
| 8. Modelled visual losses | Applied to WSE | Applied to BSE | Assuming visual losses affect only the worst seeing eye is conservative, and may underestimate HRQoL impacts |
|  |  | Applied to both eyes |  |
| 9. Disutility of VA loss | Based on EQ-5D | Based on VHQ | Applying EQ-5D based utilities may underestimate effects of visual losses on HRQoL |
| 10. Treatment costs for DMO and proliferative DR | Applied for unilateral disease | Inflated by 40% and 90% respectively, to account for bilateral treatment | Assuming all treatment of diabetic retinopathy is unilateral (for a single eye) is conservative. Treatment costs are inflated to assess impact of applying expected proportions of patients (based on expert clinical opinion) requiring treatment to both eyes at some point |
| 11. Anti-VEGF drug costs | NHS indicative price of aflibercept (£816 per vial) | Discounted by 30% | Anti-VEGF drugs are available to the NHS at undisclosed, confidential discounted prices. A range of plausible discounts has been applied to cover this uncertainty. |
|  |  | Discounted by 40% |  |
|  |  | Discounted by 50% |  |
|  |  | Discounted by 60% |  |
|  |  | Discounted by 70% |  |
| 12. Fenofibrate dosing for reduced eGFR | 200 mg every second day | 67mg every day | In LENS, patients with eGFR <60 ml/min/1.73 m2, received the standard dose every second day. The scenario assesses the impact of using a reduced daily dose. |
| 13. Ophthalmology referral/treatment costs | Scottish speciality costs | English HRG based NHS reference costs | To explore the impact of geographic price variability on cost-effectiveness findings |
| 14. Fenofibrate price | Scottish drug tariff (£4.77) | English drug tariff (£2.69) | To explore the impact of geographic price variability on cost-effectiveness findings |
| 15. Ophthalmology referral/treatment costs and fenofibrate price | Scottish specialty cost and Scottish drug tariff | English HRG based NHS reference costs and English drug tariff | To explore the impact of geographic price variability on cost-effectiveness findings |
| 16. Fenofibrate additional monitoring costs | Assumed negligible | Additional check of renal function 1-2 months after starting fenofibrate (accounting for GP and nurse time) | To explore possibility of an additional renal function check following initiation of fenofibrate treatment |
| 17. Fenofibrate prescribing costs | Assumed absorbed as part of routine management of diabetes | Allocate an additional GP appointment to initiate treatment | To cover possibility that initiating treatment generates an additional GP appointment on top of standard diabetes management and follow-up |
| 18. Background health state costs | Equalised across treatment arms and health states | Estimated non-significant difference in 6-montly costs by treatment arm applied | Aligns with best estimate of mean difference in 6-monthly background health state costs for fenofibrate versus placebo from the trial-based analysis:  -£113 (-275 to 49). |

**Supplementary Table 9. Model based cost-effectiveness subgroup analysis results (fenofibrate versus standard care)**

| **Population/Subgroups** | **Subgroup categories** | **Incremental cost (£)** | **Incremental QALYs** | **ICER (£)** |
| --- | --- | --- | --- | --- |
| **Full population (base case)** | | 6 | 0.015 | 406 |
| Type of diabetes | Type 1 | -6 | 0.016 | Dominant |
|  | Type 2 | 13 | 0.015 | 890 |
| HbA1c level | <64 mmol/mol (DCCT <8%) | 64 | 0.013 | 4,962 |
|  | ≥64 mmol/mol  (DCCT ≥8%) | -49 | 0.018 | Dominant |
| Baseline maculopathy grade | None | 19 | 0.014 | 1,408 |
|  | Observable (M1) | -120 | 0.019 | Dominant |
| ICER, incremental cost-effectiveness ratio; HbA1c, glycated haemoglobin | | | | |
