# Supplementary material for: Cost-effectiveness of fenofibrate versus standard care for reducing the progression of diabetic retinopathy: an economic evaluation based on data from the LENS trial
Source: Diabet Med. Author manuscript; Available in PMC 2025 Jul 17. (PMC7617897; doi:10.1111/dme.70098)
Supplement: Supplementary file 1 [file EMS206825-suppement-Appendix.pdf]

Appendix: Members of the LENS Collaborative Group

Members of the LENS Collaborative Group include colleagues listed below and the manuscript authors.

Paula Williamson, Jonathan Bodansky, Allan Cairns, Sue Dickie, Gillian Hallard, Gozie Joe Adigwe, Laura Jones, Timothy Lyons, Isla Mackenzie, Dermot Neely, Sarah Wild, Ian Young, Praveen Patel, Alan Watkins, Natalie Staplin, Sarah Howard, Kate Sawyer, Karen Taylor, Patricia Achiri, Andy Burke, Susan Hurley, Gareth McChlery, Kevin Murphy, Sandra Pickworth, Alison Timadjer, Kate Gardiner, Monique Willett, Liz Wincott, Matthew Beesley, Charles Cutting, Monika Raj, Ian Roure, Michelle Goonasekera, Graham Bagley, Anne Whitehouse, Isobel Young, Gavin Brown, Kate Bird, Heather Halls, Guanguo Cui, Charles Daniels, Angela Field, Bob Goodenough, Youcef Mostefai, Samee Syed, Angela Chamberlain, Carol Knott, Anna Liew, Juliette Meek, Meera Mistry, Andrea Wilson, Patrick Zettegren, Rach AitSadi, Ian Barton, Alex Baxter, Clive Berry, Rijo Kurien, Michael Lay, Aleksandra Murawska, James Thompson, Allen Young, Enti Spata, Rachel Raff, Crispian Howard, Diann Taggart, Paul Connelly, Mohan Varikkara, Scott Blackwell, Fiona Elliott, Jacqueline Locke, Angela Sweeney, Stephen Wood, Olive Herlihy, Jeni Darling, Jill Little, Ewan Bell, Susan Boytha, Paula Neill, Andrew Blaikie, Laura Beveridge, Tina Coventry, Susan Fowler, Karen Gray, Linda Lister, Amanda McGregor, Sandra Pirie, Evgeniya Postovalova, Anna White, John Doig, Stephanie Brogan, Margie Duncan, Angela Gilmour, Lynn Ryan, Anne Todd, Lesley Whitelaw, Elaine Wilson, Sam Philip, Ann Cadzow, Sharlene Greig, Siby Joseph, Lynne Murray, Julie Toye, Sonia Zachariah, David Carty, Lindsey Bailey, Alison Begg, Katherine Brown, Charlotte Clark, Katharine Duffy, Shirley Fawcett, Adam Gill, Emma Hughes, David Jenkins, Laila Martin, Christine McAllister, Mhairi MciIntyre, Leeanne Milne, Erin Murphy, Hilary Peddie, Katharine Sharp, Susan Speirs, Joyce Thompson, Lynne Turner,

Simon Hewick, Clare Bradley, Jim Finlayson, Laura O'Keeffe, Meena Virdi, Soo Park, Murdina Bell, Maureen Brown, Linda Macliver, Jackie Quigley, Karen Madill, Daniel Beck, Freddie Burgess, Karen Charlwood, Lauren Finlayson, Nicola Freeman, Margaret McDonald, Carla Pettifor, Karen Sedstrem, Alice Thomson, Ellen Malcolm, Jackie Lindsay, Lisa Stewart, Helen Colhoun, Robert Lindsay, John Olson, Naveed Sattar

Supplementary Tables

Supplementary Table 1. NHS Scotland's Diabetic Eye Screening Programme grading scheme

Grade	Description	Findings	Outcome
**RETINOPATHY (excluding the macula)**			
R0	No DR anywhere	-	Rescreen in 12-24 months
R1	Mild background diabetic retinopathy	The presence of at least one of any of the following features anywhere:<br>• dot haemorrhages / microaneurysms<br>• hard exudates<br>• cotton wool spots<br>• blot haemorrhages*<br>• superficial/ flame shaped haemorrhages	Rescreen in 12 months
R2	Observable background diabetic retinopathy	Four or more blot haemorrhages* in one hemi-field only (Inferior and superior hemi-fields delineated by a line passing through the centre of the fovea and optic disc)	Rescreen in 6 months
R3	Referable background diabetic retinopathy	Any of the following features:<br>• four or more blot haemorrhages* in both inferior and superior hemi-fields<br>• Venous beading<br>• Intraretinal Microvascular Abnormalities (IRMA)	Specialist referral (routine)
R4	Proliferative diabetic retinopathy	Any of the following features:<br>• Active new vessels<br>• Vitreous haemorrhage	Specialist referral (urgent)
R4i†	Treated proliferative diabetic retinopathy	Any of the following features:<br>• Inactive new vessels with evidence of laser treatment	Rescreen in 12 months
R6	Not adequately visualised	Retina not sufficiently visible for assessment	Technical failure
**MACULOPATHY**			
M0	No maculopathy	No features $\leq$2 disc diameters from the centre of the fovea sufficient to qualify for M1 or M2	Rescreen in 12-24 months
M1	Observable maculopathy	Lesions as specified below within a radius of >1 but $\leq$2 disc diameters from the centre of the fovea:<br>• Any hard exudates	Rescreen in 12 months
M2‡	Referable maculopathy	Lesions as specified below within a radius of $\leq$1 disc diameter of the centre of the fovea:<br>• Any blot haemorrhages*<br>• Any hard exudates	OCT surveillance scan or Rescreen in 12 months

* Blot haemorrhage has the same or greater diameter as a retinal vein crossing the optic disc

† R4i is not counted towards the primary outcome as it represents inactive disease

‡ At the start of LENS in 2018 and until end-2021, all patients in Scotland with M2 retinal screening results required referral to a specialist in ophthalmology. During 2022, the Diabetic Eye Screening programme started to introduce a phased change to the management pathway of patients with M2 disease. In the new pathway, M2 in the context of poor visual acuity (e.g. 6/9.5 or worse) leads to optical coherence tomography (OCT) imaging followed by referral to an ophthalmologist if there is evidence of macular oedema.

Supplementary Table 2 Unit costs and sources

**Resource item**	**How measured**	**Source of measurement**	**Unit cost***	**Source of valuation**
Study medication	Prescribed quantity and dose	Study database	£4.77 (Scottish Tariff)<br>2.69 (English drug tariff)<br><br>28 pack (200mg capsules)	Scottish and English Drug Tariff [12,13]
Biochemistry tests	Number of tests	Study database, linked from SCI-Diabetes	£0.90 per test	Scottish Health Service Costs [14]
GP time	Time associated with request and review of biochemistry tests	Assumption (5 minutes)	£178 per hour	Unit costs of Health and Social Care [11]
Practice nurse time	Time to obtain sample	Assumption (5 minutes)	£53 per hour	Unit costs of Health and Social Care [11]
Hospital admissions	Number of episodes, specialty, length of stay.	Linked SMR01 data	Variable by specialty	Scottish Health Service costs [14]
Hospital outpatient attendances	Number of episodes, specialty	Linked SMR00 data	Variable by specialty	Scottish Health Service costs [14]
Retinal Screening episodes	Number of episodes	DES	£44.47	Scotland et al. 2016 [16]
OCT monitoring of maculopathy (M2)	Number of episodes	DES	£41.03	Scotland et al. 2016 [16]
Slit lamp examination (screening programme)	Number of episodes	DES	£36.14	Olson et al. 2013 [15]
Prescribed medicines	Medicine name, dose, quantity	Prescribing Information System	Variable	BNF [12]
**DR related interventions**				
Ophthalmology outpatient visit	Number of visits	DES and SCI diabetes systems; trial specific	£232 (Scottish average)	Scottish Health Service Costs,

		questionnaires; supporting data from local NHS health records provided by LCCs); SMR01; and SMR00.	£154 (English average) inflated from 144 in 2021/2022 prices	NHS Reference costs [14]
Pan-retinal laser photocoagulation session	Assumptions regarding expected number of sessions (see text)		£164 inflated from 153 in 2021/2022 prices	NHS Reference costs (BZ86) [27]
Administration of anti-VEGF injection	Assumptions regarding expected number of injections (see text)		£164 inflated from 153 in 2021/2022 prices	NHS Reference costs (BZ86) [27]
Vitrectomy	Number of events		£2,573 inflated from 2,404 in 2021/2022 prices	NHS Reference costs (BZ83) [27]
Anti-VEGF drugs	Number of administrations		£551 - Ranibizumab (Lucentis); £816 - Aflibercept (Eylea)	BNF (list prices) [12]

OCT, optical coherence tomography; LCC, local coordinating centres; VEGF, vascular endothelial growth factor; BNF, British National Formulary; *Prices reflect 2022/2023 cost year

Supplementary Table 3 Summary of resource use by treatment allocation group full follow-up period

Resource use category	Fenofibrate (N=576)	Placebo (N=575)	All participants (N=1151)
Screening episodes, (mean (SD)	2.66 (1.11)	2.69 (1.13)	2.67 (1.12)
Digital retinal screening	2.48 (1.06)	2.41 (1.09)	2.44 (1.07)
OCT	0.08 (0.42)	0.13 (0.58)	0.11 (0.51)
Slit lamp	0.11 (0.46)	0.14 (0.51)	0.12 (0.49)
Biochemistry tests, mean (SD)	26.14 (13.50)	25.51 (12.74)	25.82 (13.12)
Albumin /Creatinine	2.44 (2.08)	2.37 (1.98)	2.40 (2.03)
HDL cholesterol	3.45 (2.06)	3.35 (2.04)	3.40 (2.05)
HbA1c	5.65 (2.85)	5.72 (2.84)	5.69 (2.84)
Triglycerides	2.65 (2.29)	2.54 (2.27)	2.60 (2.28)
Creatinine	8.16 (7.02)	7.77 (6.31)	7.96 (6.68)
Total cholesterol (mmol/L)	3.79 (2.09)	3.76 (2.01)	3.78 (2.05)
Prescribing episodes (episodes), mean (SD)	129.38 (71.17)	128.95 (71.08)	129.17 (71.09)
Blood pressure	32.33 (29.87)	30.82 (29.03)	31.58 (29.45)
Diabetes	67.91 (38.46)	67.81 (38.07)	67.86 (38.25)
Other	8.23 (17.17)	8.72 (16.39)	8.48 (16.78)
Oedema	1.45 (5.95)	1.82 (6.49)	1.64 (6.22)
Statins	19.45 (13.07)	19.78 (12.55)	19.62 (12.81)
Outpatient visits, n (%)	477 (82.8%)	471 (81.9%)	948 (82.4%)
Specialties of interest	351 (60.9%)	350 (60.9%)	701 (60.9%)
Ophthalmology	151 (26.2%)	152 (26.4%)	303 (26.3%)
Others	369 (64.1%)	342 (59.5%)	711 (61.8%)
Outpatient visits, mean (SD)	8.33 (11.09)	9.04 (12.27)	8.68 (11.69)
Specialties of interest	3.27 (4.70)	3.73 (5.31)	3.50 (5.01)
Ophthalmology	0.97 (2.78)	1.01 (2.78)	0.99 (2.78)
Others	4.09 (8.12)	4.30 (9.10)	4.19 (8.62)
Inpatient/day case admissions, n (%)	247 (42.9%)	247 (43.0%)	494 (42.9%)
Specialties of interest	175 (30.4%)	162 (28.2%)	337 (29.3%)
Others	142 (24.7%)	143 (24.9%)	285 (24.8%)
Inpatient/day case (episodes), mean (SD)	1.85 (4.43)	1.96 (3.80)	1.90 (4.13)
Specialties of interest	1.00 (2.08)	1.24 (2.97)	1.12 (2.57)
Others	0.85 (3.50)	0.72 (1.92)	0.79 (2.82)

SD: standard deviation, OCT: optical coherence tomography, HDL: high-density lipoprotein

Supplementary Table 4 Summary of health service costs by treatment group full follow-up period

Resource use category	Fenofibrate (N=576) (£)	Placebo (N=575) (£)	All participants (N=1151) (£)
Intervention acquisition cost	221(86)	0	N/A
Screening episodes, (mean (SD)	117 (49)	118 (49)	117 (49)
Digital retinal screening	110 (47)	107 (48)	109 (48)
OCT	3 (17)	5 (23)	4 (21)
Slit lamp	4 (17)	5 (18)	4 (18)
Biochemistry tests, mean (SD)	216 (158)	210 (143)	213 (150)
Albumin /Creatinine	2 (2)	2 (2)	2 (2)
HDL cholesterol	3 (2)	3 (2)	3 (2)
HbA1c	5 (3)	5 (3)	5 (3)
Triglycerides	2 (2)	2 (2)	2 (2)
Creatinine	7 (6)	7 (6)	7 (6)
Total cholesterol	3 (2)	3 (2)	3 (2)
GP and nurse time	194 (147)	188 (133)	191 (140)
Prescribing episodes (episodes), mean (SD)	3,132 (2,199)	3,116 (2,220)	3,124 (2,209)
Blood pressure	102 (248)	111 (287)	107 (268)
Diabetes	2,835 (2,126)	2,810 (2,117)	2,823 (2,121)
Other	155 (490)	151 (454)	153 (472)
Oedema	2 (10)	3 (12)	3 (11)
Statins	37 (23)	40 (65)	39 (49)
Outpatient visits, mean (SD)	2,179 (4,171)	2,226 (3,001)	2,202 (3,633)
Specialties of interest	1,051 (1,484)	1,167 (1,553)	1,109 (1,520)
Ophthalmology	226 (646)	235 (644)	230 (645)
Others	1,127 (3,341)	1,059 (2,222)	1,093 (2,837)
Anti-VEGF drug cost	107(855)	127(904)	117(879)
Inpatient/day case (episodes), mean (SD)	5,207 (13,170)	6,135 (20,803)	5,671 (17,405)
Specialties of interest	2,475 (8,128)	3,198 (17,819)	2,836 (13,844)
Others	2,731 (9,144)	2,937 (8,407)	2,834 (8,780)
Inpatients/day case (continuous inpatient stays), mean (SD)	5,207 (13,170)	6,135 (20,803)	5,671 (17,405)
Specialties of interest	3,073 (10,034)	3,987 (18,952)	3,529 (15,160)
Others	2,134 (7,965)	2,149 (7,033)	2,141 (7,511)
SD: standard deviation, OCT: optical coherence tomography, HDL: high-density lipoprotein			

OCT: Optical Coherence Tomography

Supplementary Table 5 Trial based cost-effectiveness scenario analysis results at two years (fenofibrate versus standard care)

Population/Subgroups	Base case	Scenario	Incremental cost (£)	Incremental QALYs	ICER (£)
**Base case**			-254 (-1062 to 624)	0.044 (0.013 to 0.08)	Dominant
Price discount on anti-VEGF treatment	List price	30%	-249 (-1062 to 627)	0.044 (0.013 to 0.08)	Dominant
		70%	-243 (-1048 to 642)	0.044 (0.013 to 0.08)	Dominant
Hospital costs	Prior specified specialties of interest	All specialties	-741(-1949 to 425)	0.044 (0.013 to 0.08)	Dominant

Supplementary Table 6 Within trial comparison of six-month interval-based costs by treatment group

	Fenofibrate			Placebo			
Six monthly intervals	N	Mean cost (£)*	SE	N	Mean cost (£)*	SE	Difference between groups (95% CI)
1	576	690	101	575	808	118	-118 (-421, 186)
2	573	837	122	573	1,317	193	-481 (-927, -34)
3	570	672	99	571	734	108	-62 (-348, 223)
4	566	973	143	568	896	132	77 (-304, 457)
5	560	730	108	564	961	142	-230 (-579, 118)
6	549	981	147	549	875	131	106 (-278, 491)
7	509	930	144	513	1,080	167	-151 (-582, 281)
8	467	961	160	459	746	125	214 (-173, 602)
9	298	761	166	296	1,125	246	-364 (-907, 178)
10	103	2,003	748	105	1,445	538	558 (-1104, 2221)
Average over trial follow-up	576	844	49	575	945	55	-101 (-243, 42)

*Adjusted for minimisation covariates which included categories of age, type of diabetes, sex, HbA1c, renal function, statin use, baseline retinopathy grade, and baseline maculopathy grade.
SE, standard error; N, number; CI, confidence interval

Supplementary Table 7 Model input parameters

Parameters	Value	SE or (95% CI)	Distributional form	Source
Settings				LENS trial
Start age	61 (12.4)		1st order variation	
Proportion Male	0.73		1st order variation	
Cycle length years	0.5			
Cycle length days	182.63			
Discount rate (costs)	0.035			
Discount rate (QALYs)	0.035			
Clinical efficacy inputs (hazards expressed per 6-month model cycle)				
Non referable to referable DR, with or without concurrent referable maculopathy (exponential function)				LENS trial
Hazard rate	0.006	0.004 – 0.009	Multivariate normal (log scale)	
Hazard ratio (Feno)*	1.309	0.746 – 2.295		
Non referable to referable maculopathy, alone (Weibull function)				LENS trial
Scale	0.052	0.041 – 0.068	Multivariate normal (log scale)	
Shape	0.857	0.761 – 0.965		
Hazard ratio (Feno)*	0.661	0.513 – 0.851		
Hazard ratio for the primary composite outcome (from parametric Weibull regression)				LENS trial
Hazard ratio (Feno)+	0.73	(0.58 – 0.92)	Lognormal	
Referable maculopathy to referable DR (exponential function)				LENS trial
Hazard rate	0.020	0.012 – 0.035	Multivariate normal (log scale)	
Hazard ratio (Feno)	1.457	0.665 – 3.194		
Referable DR to referable maculopathy (exponential function)				LENS trial
Hazard rate	0.036	0.009 – 0.144	Multivariate normal (log scale)	
Hazard ratio (Feno)	0.740	0.104 – 5.254		
Referable maculopathy to treatment for DMO (exponential function)				LENS trial
Hazard rate	0.016	0.009 – 0.030	Multivariate normal (log scale)	
Hazard ratio (Feno)	0.682	0.237 – 1.963		
Referable DR to treatment for referable DR with standard care (log normal distribution)				LENS trial
Constant	0.679	-0.425 – 1.784	Multivariate normal (log scale)	
lnSigma	0.951	0.560 – 1.341		
Referable DR to treatment for referable DR with fenofibrate (log normal distribution)				LENS trial
Constant	2.345	1.291 – 3.399	Multivariate normal (log scale)	
lnSigma	0.445	-0.076 – 0.967		
Other clinical inputs				
Hazard for fenofibrate discontinuation	0.034	0.003	Normal	LENS trial

Probability of death	Age and sex specific UK life tables		NA	ONS, 2024
Standardised mortality ratio (Type 1 diabetes, male)	2.95	2.74 – 3.18	Log normal	O'Reilly et al.[20]
Standardised mortality ratio (Type 1 diabetes, female)	4.62	4.20 – 5.08	Log normal	O'Reilly et al.[20]
Standardised mortality ratio (Type 2 diabetes, male)	1.36	1.28 – 1.45	Log normal	Collier et al.[19]
Standardised mortality ratio (Type 2 diabetes, female)	1.48	1.38 – 1.59	Log normal	Collier et al.[19]
Proportion RDR treated patients receiving PRP	1		NA	LENS trial
Proportion PRP treated patients also receiving vitrectomy	0.11	0.06	Beta (α=3; β=24)	LENS trial
Proportion of fenofibrate prescriptions that are once daily	0.72	0.005	Beta (α=6392; β=2483)	LENS trial
Proportion of patients first developing RDR that have concurrent referable maculopathy at time of referral	0.34	0.07	Beta (α=17; β=33)	LENS trial
Proportion of patients developing RDR that are treated immediately on progression	0.04	0.02	Beta (α=3; β=72)	LENS trial
Proportion of patients developing referable maculopathy that are treated immediately on progression	0.015	0.007	Beta (α=4; β=262)	LENS trial
Proportion of RDR treated patients requiring bilateral treatment*	0.9	0.09 (assumed)	Beta	Expert opinion
Proportion of referable maculopathy treated patients requiring bilateral treatment*	0.4	0.04 (assumed)	Beta	Expert opinion
Health state utility values				
Baseline utility	0.811 (0.23)		1st order variable	LENS trial
Utility by health state and visual acuity status				
Constant	0.142	0.037	Multivariate normal	LENS trial
Time from baseline (days)	-0.00003	0.00001		
Post referable disease	-0.020	0.011		
Baseline age	-0.0013	0.0005		
Baseline EQ-5D	0.885	0.024		
Utility coefficient for log ETDRS letters in BSE	0.1	0.027	Normal	Brazier et al.[23]

Utility coefficient for log ETDRS letters in WSE	0.042	0.014	Normal	Brazier et al.[23]
Resource use and costs				
Fenofibrate micronised 200mg capsules (price per pack)	£4.77		NA	Scottish drug tariff[13]
Fenofibrate micronised 200mg capsules (pack size)	28		NA	
Fenofibrate 67mg micronised capsules (price per pack)	£23.23		NA	Scottish drug tariff[13]
Fenofibrate 67mg micronised capsules (pack size)	90			
Cost of fenofibrate treatment per cycle (assuming 200mg every other day for those with reduced eGFR)	£26.76		Calculated	
Cost of fenofibrate treatment per cycle (assuming 67mg every day for those with reduced eGFR)	£47.14		Calculated	
Health state background HCRU costs	£864.10	£39.02	Normal	LENS trial
Average screening costs per six-month model cycle by cycle number and referable maculopathy status Log e linear predictors)				
Constant	2.53	0.027	Multivariate normal	LENS trial
Cycle number (six-monthly)	0.02	0.005		
Referable maculopathy	0.31	0.036		
Cost per ophthalmology outpatient appointment (Scotland)	£232	23.20 (assumed)	Normal	Scottish Health Service costs[14]
Cost per ophthalmology outpatient appointment (England)	£133		NA	NHS Reference costs[27]
Cost per panretinal laser photocoagulation session (Scotland)	£232	23.20 (assumed)	Normal	Scottish Health Service costs[14]
Cost per panretinal laser photocoagulation session (England)	£159		NA	NHS Reference costs[27]
Cost of vitrectomy (unilateral)	£2,573	257.30 (assumed)	Normal	NHS Reference costs[27]
Cost of aflibercept (per injection)	£816		NA	BNF[12]
Cost of ranibizumab (per injection)	£551		NA	BNF[12]
Cost per administration of an anti-VEGF injection (Scotland)	£232	23.20 (assumed)	Normal	Scottish Health Service costs[14]

Cost per administration of an anti-VEGF injection (England)	£159		NA	NHS Reference costs[27]
Number of anti-VEGF injections in year 1 of treatment for DMO	6.3	0.13 (approximated)	Gamma	Sivaprasad et al. [17]
Number of anti-VEGF injections in year 2 of treatment for referable DMO	2.9	0.115 (approximated)	Gamma	Sivaprasad et al.[17]
Number of anti-VEGF injections years 3-5	2.45	0.25	Gamma	Glassman et al.[22]
Number of additional outpatient appointments for anti-VEGF treatment monitoring	1.0		NA	Assumption
Number of additional outpatient appointments for PRP treatment monitoring	1.0		NA	Assumption
Number of PRP sessions to achieve stable PDR (assumed over 12 weeks)	2.77	0.015 (approximated)	Gamma	Yusuf et al.[26]

[+]Used in the base case for effect of fenofibrate on progression to RDR and RMO; *applied in scenario analysis

DR: diabetic retinopathy, MO: maculopathy, RDR: referable diabetic retinopathy, PRP: panretinal photocoagulation, VEGF: vascular endothelial growth factor, ETDRS: Early Treatment Diabetic Retinopathy Study, BSE: better seeing eye, WSE: worse seeing eye, eGFR: estimated glomerular filtration rate, HCRU: Health and Care Research Unit, PDR: proliferative diabetic retinopathy, Feno.: fenofibrate

Supplementary Table 8 Model based cost-effectiveness scenario analyses and their justification

Parameter / assumption	Base case	Scenario	Justification
1. Time horizon	10 years	a) 5 years	The duration over which differences in cost and benefits are extrapolated will influence cost-effectiveness
		b) 20 years	
		c) 30 years	
2. Time to referable DR and referable MO	Single overall treatment effect	Cause specific treatment effects	Fenofibrate may have different effects on progression of maculopathy and retinopathy
3. Time to referable MO	Weibull curve	Exponential curve	Provided the next best fitting curves for time to referable maculopathy
		Log logistic curve	
4. Time to referable retinopathy (R3/R4)	Exponential	Gompertz	Provided the next best fitting curves for time to referable retinopathy
		Log logistic	
5. Treatment effects of fenofibrate	Continuous proportional hazards	Waned from 5 years	The duration of effects of fenofibrate beyond the trial follow-up are uncertain
6. Effect of fenofibrate on post-progression transitions	Estimated from the data	Assume no effect	Ongoing effects of fenofibrate on progression, following first progression event, are uncertain
7. Time from referable retinopathy (R3/R4) to treatment for referable retinopathy	Lognormal (both arms)	Exponential (both arms)	Alternative plausible extrapolations of time from referable DR to treatment, avoiding curves crossing before ten years
		Weibull (both arms)	
		Lognormal (SC), exponential (feno)	
		Exponential (SC), Gompertz (feno)	

8. Modelled visual losses	Applied to WSE	Applied to BSE	Assuming visual losses affect only the worst seeing eye is conservative, and may underestimate HRQoL impacts
		Applied to both eyes	
9. Disutility of VA loss	Based on EQ-5D	Based on VHQ	Applying EQ-5D based utilities may underestimate effects of visual losses on HRQoL
10. Treatment costs for DMO and proliferative DR	Applied for unilateral disease	Inflated by 40% and 90% respectively, to account for bilateral treatment	Assuming all treatment of diabetic retinopathy is unilateral (for a single eye) is conservative. Treatment costs are inflated to assess impact of applying expected proportions of patients (based on expert clinical opinion) requiring treatment to both eyes at some point
11. Anti-VEGF drug costs	NHS indicative price of aflibercept (£816 per vial)	Discounted by 30%	Anti-VEGF drugs are available to the NHS at undisclosed, confidential discounted prices. A range of plausible discounts has been applied to cover this uncertainty.
		Discounted by 40%	
		Discounted by 50%	
		Discounted by 60%	
		Discounted by 70%	
12. Fenofibrate dosing for reduced eGFR	200 mg every second day	67mg every day	In LENS, patients with eGFR <60 ml/min/1.73 m2, received the standard dose

			every second day. The scenario assesses the impact of using a reduced daily dose.
13. Ophthalmology referral/treatment costs	Scottish speciality costs	English HRG based NHS reference costs	To explore the impact of geographic price variability on cost-effectiveness findings
14. Fenofibrate price	Scottish drug tariff (£4.77)	English drug tariff (£2.69)	To explore the impact of geographic price variability on cost-effectiveness findings
15. Ophthalmology referral/treatment costs and fenofibrate price	Scottish specialty cost and Scottish drug tariff	English HRG based NHS reference costs and English drug tariff	To explore the impact of geographic price variability on cost-effectiveness findings
16. Fenofibrate additional monitoring costs	Assumed negligible	Additional check of renal function 1-2 months after starting fenofibrate (accounting for GP and nurse time)	To explore possibility of an additional renal function check following initiation of fenofibrate treatment
17. Fenofibrate prescribing costs	Assumed absorbed as part of routine management of diabetes	Allocate an additional GP appointment to initiate treatment	To cover possibility that initiating treatment generates an additional GP appointment on top of standard diabetes management and follow-up
18. Background health state costs	Equalised across treatment arms and health states	Estimated non-significant difference in 6-montly costs by treatment arm applied	Aligns with best estimate of mean difference in 6-monthly background health state costs for fenofibrate versus placebo from the trial-based analysis: -£113 (-275 to 49).

Supplementary Table 9. Model based cost-effectiveness subgroup analysis results (fenofibrate versus standard care)

Population/Subgroups	Subgroup categories	Incremental cost (£)	Incremental QALYs	ICER (£)
**Full population (base case)**		6	0.015	406
Type of diabetes	Type 1	-6	0.016	Dominant
	Type 2	13	0.015	890
HbA1c level	<64 mmol/mol (DCCT <8%)	64	0.013	4,962
	≥64 mmol/mol (DCCT ≥8%)	-49	0.018	Dominant
Baseline maculopathy grade	None	19	0.014	1,408
	Observable (M1)	-120	0.019	Dominant

ICER, incremental cost-effectiveness ratio; HbA1c, glycated haemoglobin

Supplementary Figure 1. Comparison of model output against observed Kaplan-Meier based estimates of time to any referable diabetic retinopathy

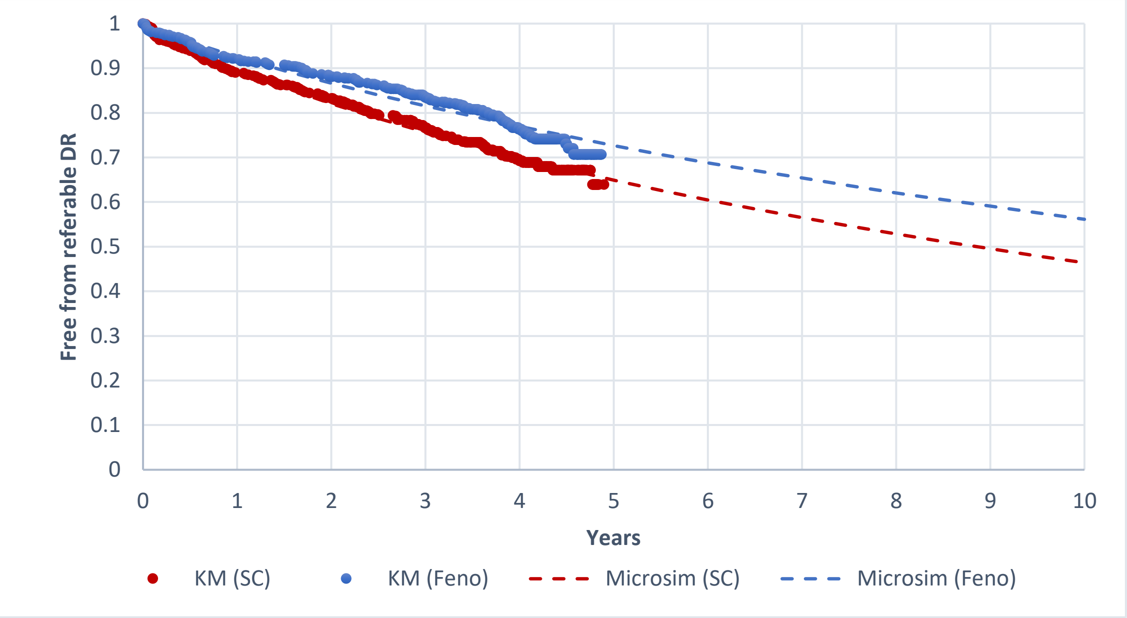

Notes: DR, diabetic retinopathy; KM, Kaplan-Meier; Microsim, Microsimulation model; SC, standard care; Feno, Fenofibrate.

Supplementary Figure 2. Comparison of model output against observed Kaplan-Meier based estimates of time to any treatment for diabetic retinopathy

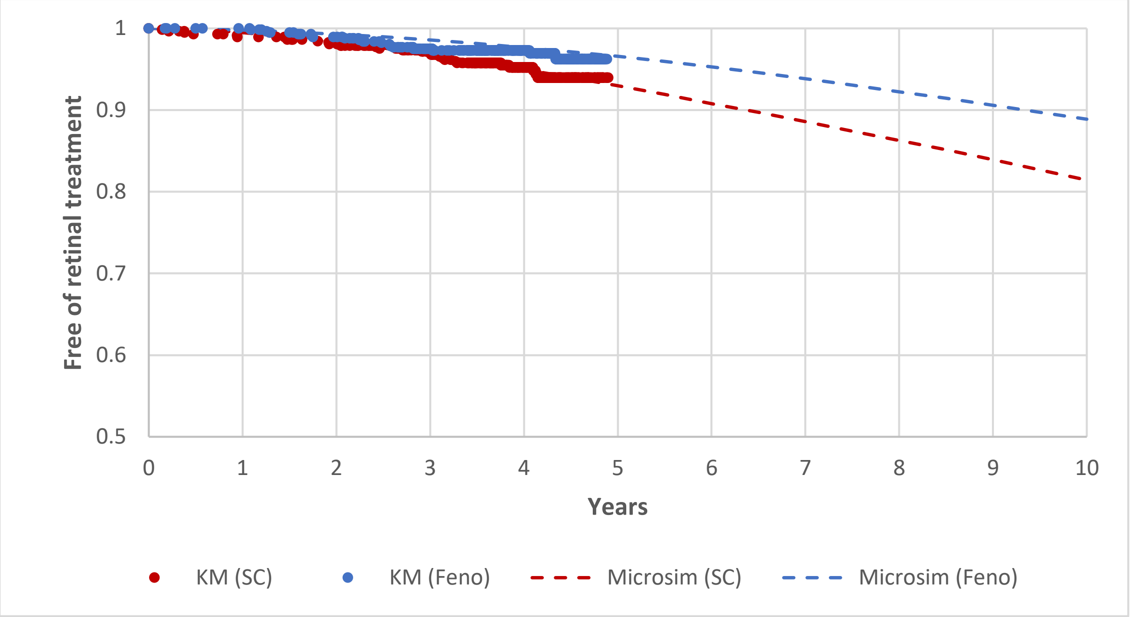

Notes: DR, diabetic retinopathy; KM, Kaplan-Meier; Microsim, Microsimulation model; SC, standard care; Feno, Fenofibrate.

Supplementary Figure 3 Cost-effectiveness acceptability curves for fenofibrate, showing the impact of price discounts on anti-VEGF drugs (aflibercept) used to treat diabetic macular oedema.

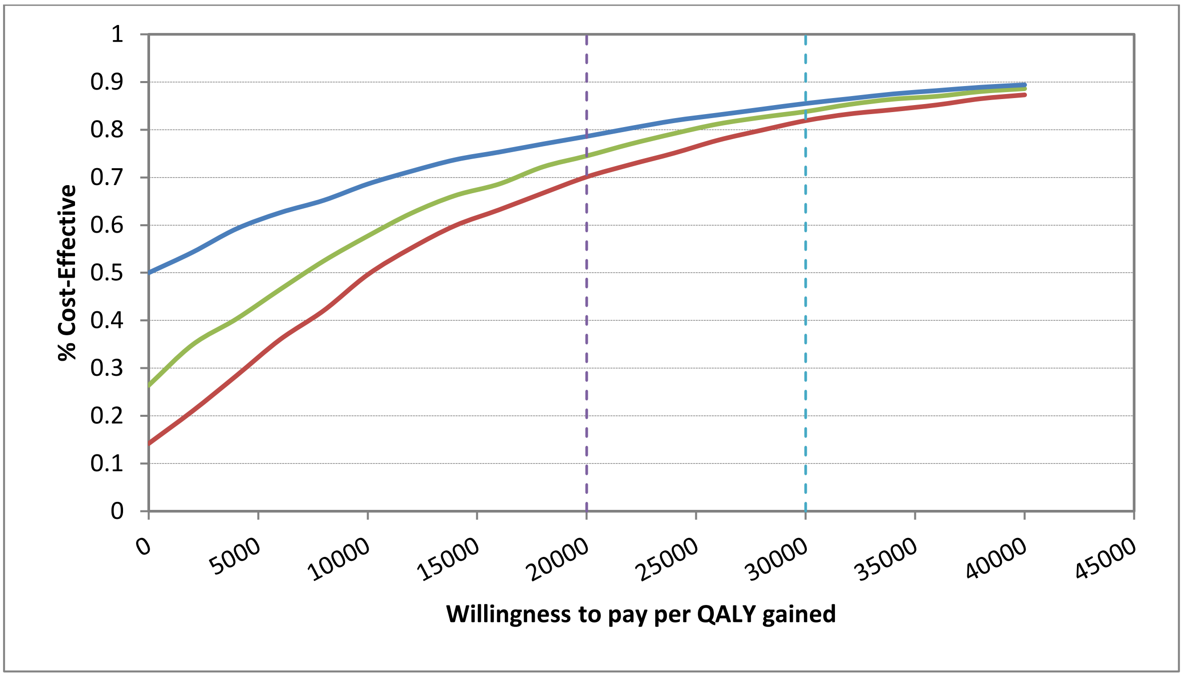

Notes: Blue, no discount on price of antiVEGF drugs; Red, 70% discount on antiVEGF drugs; Green, 70% discount on antiVEGF drugs but including bilateral treatment.

Supplementary online appendix

This appendix has been provided by the authors to give readers additional information about their work:

Cost-effectiveness of fenofibrate versus standard care for reducing the progression of diabetic retinopathy: an economic evaluation based on data from the LENS trial.

Graham Scotland, Mekazin Tsehaye, Caroline Styles, Jennifer Logue, Emily Sammons, Mohammed Zayed, Jonathan Emberson, Rachel Wade, Karl Wallendszus, Will Stevens, Rosanna Cretney, Simon Harding, Graham Leese, Gemma Currie, Jane Armitage, David Preiss, for the LENS collaborative group

Contents

List of Figures ... 3

List of Tables ... 5

Supplementary Appendix 1 – within trial analysis methods ... 6

Further details of costing methods ... 6

Analysis methods ... 8

Assessment of proportional hazards assumption for the primary clinical outcome ... 8

Parametric curve selection for the primary clinical outcome ... 10

Supplementary Appendix 2 – Decision modelling methods ... 13

Economic model structure ... 13

Deriving transition probabilities ... 13

Observable diabetic retinopathy to referable retinopathy or referable maculopathy ... 13

Time to referable maculopathy or treatment for diabetic macular oedema ... 14

Time to referable retinopathy (R3/R4) ... 18

Models of time to referable retinopathy (R3/R4) and referable maculopathy, inclusive of covariates ... 18

Combining cause specific hazards for first progression event ... 18

Post progression transition probabilities ... 23

Referable maculopathy to treatment for diabetic macular oedema ... 24

Referable DR (R3/R4) to treatment for referable DR ... 27

Referable maculopathy to referable diabetic retinopathy (R3/R4) ... 30

Referable diabetic retinopathy (R3/R4) to referable maculopathy ... 33

Health state utility impact of progression ... 36

Modelled long-term changes in visual acuity and impact on quality of life ... 36

Modelled resource use assumptions ... 38

Microsimulation model analysis ... 39

Model validation ... 40

List of Figures

Figure 1 Kaplan Meier plot for the LENS trial primary outcome. 9
Figure 2 Log cumulative hazard plot for the LENS trial primary outcome....................9
Figure 3 Schoenfeld residuals plot for the LENS trial primary outcome, with respect to treatment allocation....................10
Figure 4 Parametric survival curves for the LENS trial primary outcome in the placebo (Standard care) arm....................11
Figure 5 Parametric survival curves for the LENS trial primary outcome in the fenofibrate arm 11
Figure 6 Log cumulative hazard plot for time to referable maculopathy 15
Figure 7 Parametric survival curve fits for time to referable maculopathy in the placebo (standard care) arm 16
Figure 8 Parametric survival curve fits for time to referable maculopathy in the fenofibrate arm 17
Figure 9 Preferred curve fits for time to referable maculopathy 17
Figure 10 Log cumulative hazard plot for time to referable retinopathy or laser treatment....20
Figure 11 Parametric survival curve fits for time to referable diabetic retinopathy in the placebo (standard care) arm 21
Figure 12 Parametric survival curve fits for time to referable diabetic retinopathy in the fenofibrate arm....................22
Figure 13 Selected curve fits for time to referable diabetic retinopathy (R3/R4) 22
Figure 14 Log cumulative hazard plot for referable maculopathy to treatment for diabetic macular oedema 24
Figure 15 Parametric survival curve fits for referable maculopathy to treatment for diabetic macular oedema in the placebo (standard care) arm....................25
Figure 16 Parametric survival curve fits for referable maculopathy to treatment for diabetic macular oedema in the fenofibrate arm 26
Figure 17 Selected curve fits for referable maculopathy to treatment for diabetic macular oedema 26
Figure 18 Log cumulative hazard plot for referable retinopathy to treatment for referable retinopathy 27

Figure 19 Parametric survival curve fits for time from referable retinopathy to treatment for diabetic retinopathy in the placebo (standard care) arm 28
Figure 20 Parametric survival curve fits for time from referable retinopathy to treatment for diabetic retinopathy in the fenofibrate arm 29
Figure 21 Selected curve fits for time from referable retinopathy (R3/R4) to treatment for diabetic retinopathy 29
Figure 22 Log cumulative hazard plot for time from referable maculopathy to referable diabetic retinopathy 30
Figure 23 Parametric survival curve fits for time from referable maculopathy to referable diabetic retinopathy in the placebo (standard care) arm 31
Figure 24 Parametric survival curve fits for time from referable maculopathy to referable diabetic retinopathy in the fenofibrate arm 32
Figure 25 Parametric survival curve fits for time from referable maculopathy to referable diabetic retinopathy 32
Figure 26 Log cumulative hazard plot for time from referable retinopathy (R3/R4) to referable diabetic maculopathy 33
Figure 27 Parametric survival curve fits for time from referable retinopathy to referable maculopathy in the placebo (standard care) arm 34
Figure 28 Parametric survival curve fits for time from referable retinopathy to referable maculopathy in the fenofibrate arm 35
Figure 29 Selected Parametric survival curve fits for time from referable retinopathy to referable maculopathy 35

List of Tables

Table 1 Proportional hazards test with respect to treatment allocation 10

Table 2 Akaike and Bayesian information criteria for different parametric models fitted to the LENS primary outcome data 12

Table 3 Proportional hazards test for time to referable maculopathy with respect to treatment allocation...15

Table 4 Akaike and Bayesian information criteria of different parametric models fitted to time to referable maculopathy..16

Table 5 Proportional hazards test for time to referable retinopathy (R3/R4) or laser with respect to treatment allocation..20

Table 6 Akaike and Bayesian information criteria of different parametric models for time to referable retinopathy (R3/R4) 21

Table 7 Proportional hazards test for referable maculopathy to treatment for diabetic macular oedema with respect to treatment allocation group 24

Table 8 Akaike and Bayesian information criteria of different parametric models of time from referable maculopathy to treatment for diabetic macular oedema..25

Table 9 Proportional hazards test for referable retinopathy to treatment for referable retinopathy with respect to treatment allocation group..27

Table 10 Akaike and Bayesian information criteria of parametric models of time from referable retinopathy to treatment for referable retinopathy, fitted independently by treatment arm.....28

Table 11 Proportional hazards test for referable maculopathy to referable diabetic retinopathy by treatment allocation group 30

Table 12 Akaike and Bayesian information criteria of different parametric models of time from referable maculopathy to referable diabetic retinopathy (R3/R4) 31

Table 13 Proportional hazards test for time from referable retinopathy (R3/R4) to referable diabetic maculopathy, with respect to treatment allocation group 33

Table 14 Akaike and Bayesian information criteria of different parametric models of time from referable diabetic retinopathy R3/R4) to referable maculopathy..34

Table 15 Available EQ-5D data by time point 36

Supplementary Appendix 1 – within trial analysis methods

Further details of costing methods

In the LENS trial, drug safety was monitored by review of routinely collected blood tests (linked from SCI-Diabetes registry), which were requested at the discretion of participants' GPs or hospital doctors. Requested monitoring tests for creatinine, HbA1c, lipids and UACR were costed based on published unit costs of laboratory services in Scotland.[1] Five minutes of nurse time and five minutes of physician time, required to obtain samples and review results, were factored in for groups of tests by date of request.[2]

Retinal screening episodes (including digital retinal photography, slit-lamp examination, and OCT surveillance) were linked from NHS Scotland's Diabetic Eye Screening (DES) Programme, and costed using previously published unit cost estimates, inflated to 2022/23 prices using the NHS Cost Inflation Index.[2]

Diabetic retinopathy related referrals and interventions were captured using linked data on ophthalmology outpatient visits (from SMR00) and admissions (SMR01), combined with data on diabetic retinopathy related interventions recorded in the study database. Ophthalmology hospital activity was costed using relevant Scottish Health Service costs by specialty and setting of care (outpatient, inpatient, day case).[1] English specific NHS reference costs were tested in sensitivity analysis.[3] The number of anti-vascular endothelial growth factor (VEGF) injections administered to those commencing treatment for diabetic macular oedema (DMO) were not routinely recorded. As these are normally delivered as a course of treatment, it was necessary to assume that they would be administered at the frequency observed over two-years in an observational NHS cohort study (6.3 treatments in year one, and 2.9 treatments in year two).[4] This was further validated and considered conservative (less than expected based on recognised trial treatment protocols) based on clinical expert opinion. List prices for aflibercept treatment were applied in the base case.[5] Since this treatment is available to the NHS at an undisclosed discounted price, sensitivity analysis explored the impact of reducing prices over a range of possible discounts.

Scottish health service costs were applied to all inpatient (SMR01) and outpatient hospital activity (SMR00) occurring post-randomisation up to the final follow-up date for each participant.[1] Hospital activity was sub-divided into a priori specified clinically relevant specialties of interest (where activity could feasibly be affected by the study drug) and other specialties. Clinically relevant specialties included: General Medicine; Acute Medicine; Cardiology; Endocrinology and Diabetes; Diabetes; Renal Medicine; Vascular Surgery; Cardiac Surgery; and Ophthalmology. For the incremental cost-effectiveness analysis, we included the costs of all care episodes in clinically relevant specialties of interest.

An inpatient episode refers to a patient's stay in a specific hospital specialty, from admission to discharge (home, or to another consultant, specialty or hospital). If a patient transfers between specialties within the same hospital, they are counted as part of a continuous inpatient stay or hospital spell, with multiple episodes across different specialties. The national average unit cost per inpatient episode for each specialty of care, was sourced from Table R040 of the Scottish Health Service Cost Book.[1] We used fixed and variable cost percentage splits, as reported by the Technical Advisory Group on Resource Allocation (TAGRA) in Scotland, when applying published unit costs to episodes of inpatient care. Practically, the percentage splits represent the proportion of speciality episode costs that are considered fixed, and applicable per episode for each speciality, and the proportion that is considered variable by length of stay under each speciality. The variable component was divided by the average length of stay for each speciality, as reported in Table R040 of the Cost Book, and used to cost observed admitted patient care on per diem basis. Day case and outpatient activity was costed using the national average costs by specialty from Tables R042 and R044 and of the cost book respectively.

Medicines prescribed in the community setting, related to the management of diabetes, lipids, and blood pressure, were captured through participant record linkage to the Prescribing Information System (PIS) for Scotland. Prescribed medicines were costed using Drug Tariff prices or NHS indicative prices for proprietary medicines.[5] The linked dataset included community prescribing episodes for each patient that occurred during the LENS trial follow-up period (post-randomisation, up the date of final follow-up), with variables denoting the dispensing month, the approved drug name, and corresponding BNF code for each item prescribed. The unique name for each drug, along with its specifications, was obtained by mapping from the BNF code (BNF SNOMED mapping). Once the unique name and its

specifications were determined, the cost of the appropriate dose for the indicated use was attached to each drug. For most drugs, it was assumed they were taken according to the recommended dose, starting from the first prescription date. Accordingly, an interval of time was calculated to derive the number of drug units required during the period between consecutive prescribing dates, and associated costs were then estimated using Drug Tariff prices or NHS indicative prices for proprietary medicines. In cases where the recommended dose was not provided in the BNF, the dose was sourced from relevant literature.

Analysis methods

Assessment of proportional hazards assumption for the primary clinical outcome

This within trial cost-effectiveness analysis relied on parametric analysis of the primary time to event outcome in the LENS trial, the composite of progression of observable DR to referable DR, or any of retinal laser therapy, vitrectomy or intra-vitreal injection of medication due to DR.

The proportional hazards assumption with respect to treatment allocation was assessed by visual inspection of Kaplan-Meier data (Figure 1), assessment of the log cumulative hazard plot (Figure 2) and the Schoenfeld residuals plot (Figure 3), and by testing for a non-zero slope in the relationship between the scaled Schoenfeld residuals and time (p-values less than 0.05 indicating sufficient evidence to reject the null hypothesis of proportional hazards). The Kaplan Meier curves separate early and remain apart over the duration of follow-up. Whilst the lines of the log cumulative hazard plots do cross, this relates to the early follow-up period, and thereafter they remain apart and relatively parallel. The Schoenfeld residuals show even scatter around zero, with no obvious pattern with respect to follow-up time, and insufficient evidence to reject the null hypothesis of a constant proportional effect of treatment with fenofibrate (p=0.34).

Figure 1 Kaplan Meier plot for the LENS trial primary outcome.

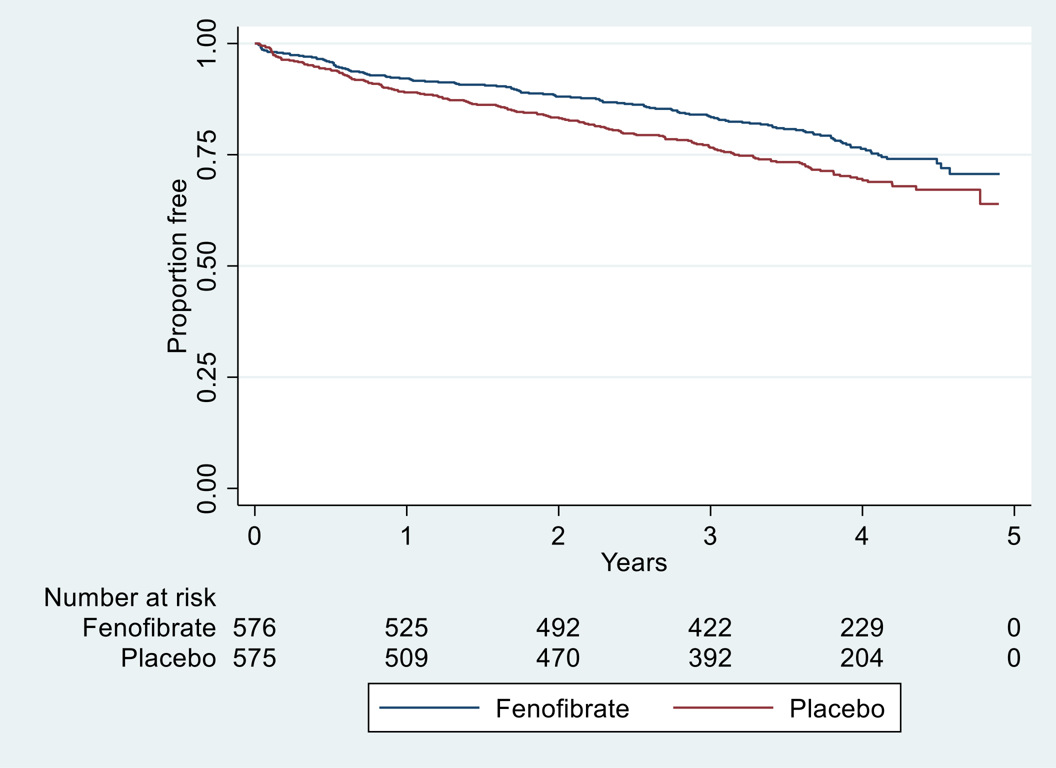

Number at risk						
Fenofibrate	576	525	492	422	229	0
Placebo	575	509	470	392	204	0

Figure 2 Log cumulative hazard plot for the LENS trial primary outcome

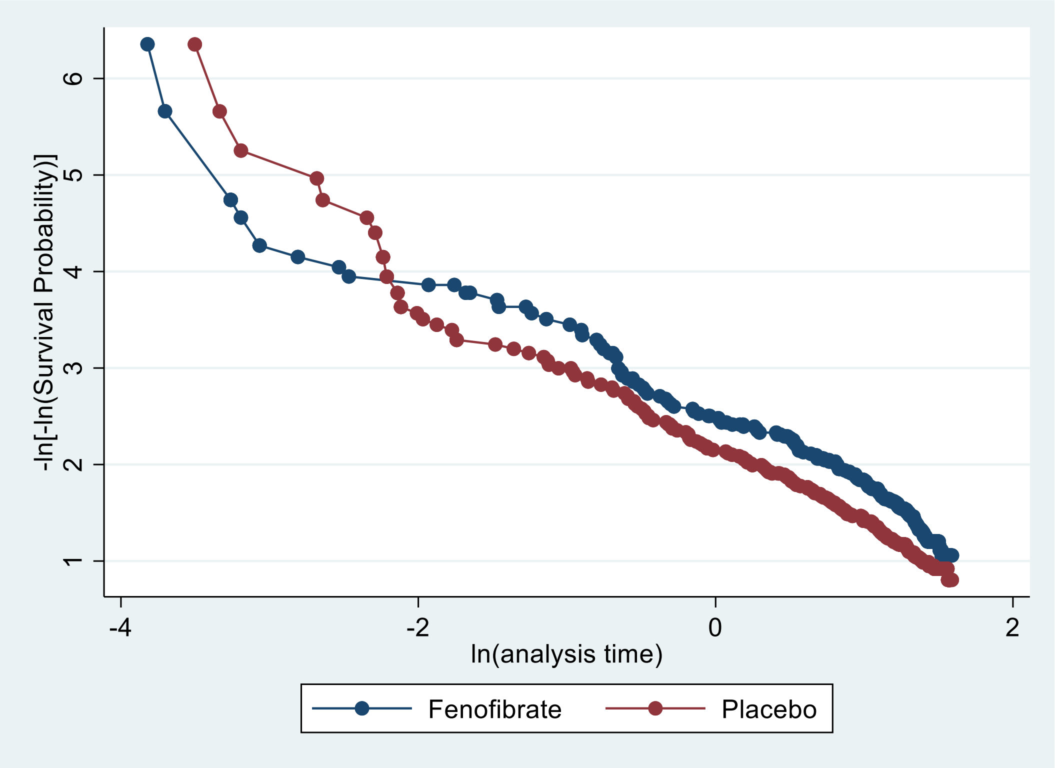

Figure 3 Schoenfeld residuals plot for the LENS trial primary outcome, with respect to treatment allocation

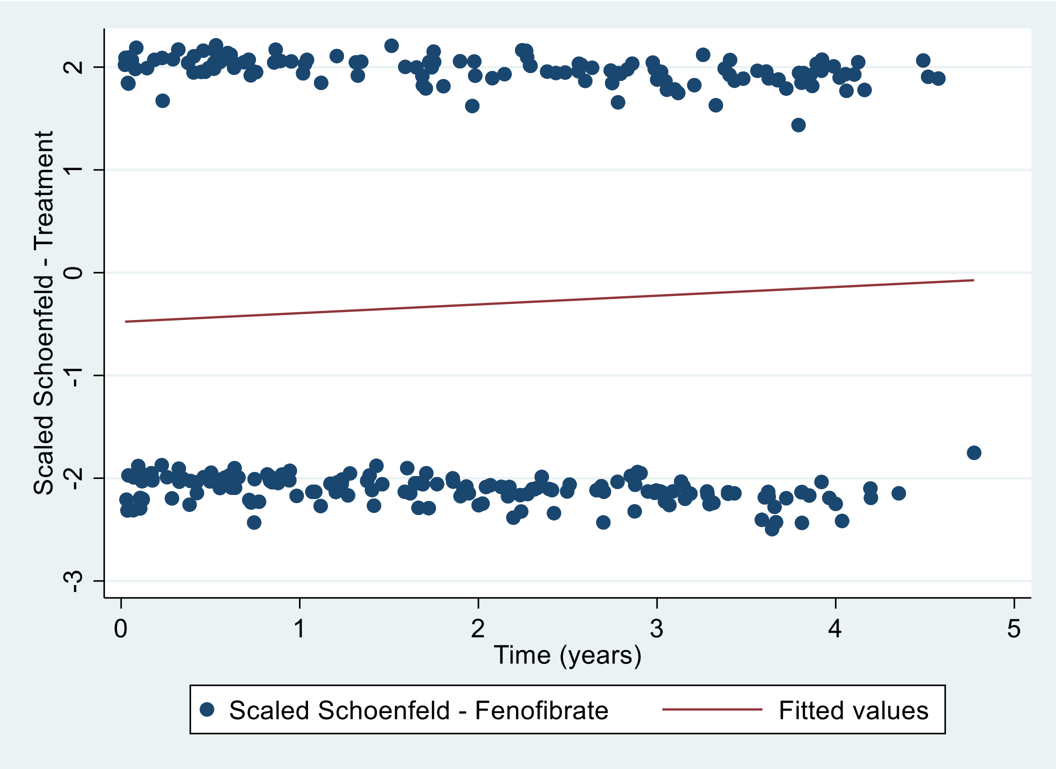

Table 1 Proportional hazards test with respect to treatment allocation

Treatment	Rho	Chi-squared	Degrees freedom	P-value
Fenofibrate	0.055	0.92	1	0.3375

Parametric curve selection for the primary clinical outcome

The support for proportional hazards placed an emphasis on assessing the fit of alternative proportional hazards models with a constant treatment effect expressed as a hazard ratio. However, accelerated failure time models were also explored. Model selection for the primary outcome was based on consideration of visual fit to the observed Kaplan Meier data (Figures 4 and 5), and statistical fit as assessed by Akaike and Bayesian information criteria (Table 2). All curves provided reasonable fit to the observed data and resulted in limited variability in referable disease-free projections. However, the Weibull model supported a diminishing hazard of progression over time and minimised the aggregate of AIC and BIC. It was therefore

chosen as the preferred parametric model for estimating risk of progression for the trial-based cost-effectiveness analysis.

Figure 4 Parametric survival curves for the LENS trial primary outcome in the placebo (Standard care) arm

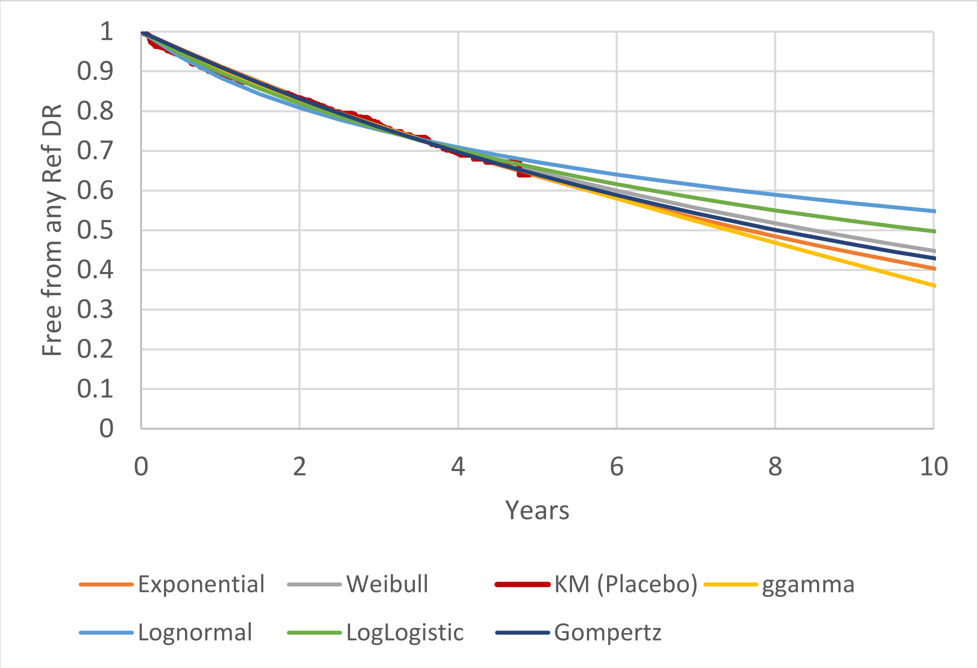

KM, Kaplan Meier; Ref, referable; DR, diabetic retinopathy

Figure 5 Parametric survival curves for the LENS trial primary outcome in the fenofibrate arm

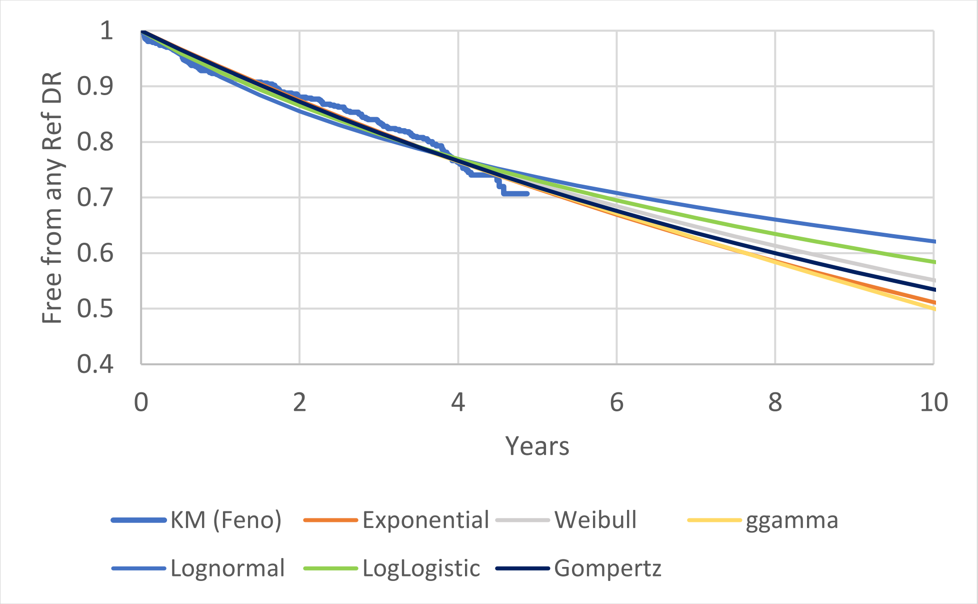

KM, Kaplan Meier; Ref, referable; DR, diabetic retinopathy; Feno, fenofibrate

Table 2 Akaike and Bayesian information criteria for different parametric models fitted to the LENS primary outcome data

Parametric function	AIC	BIC	Average
Exponential	2028.23	2038.33	2033.28
Weibull	2024.89	2040.03	2032.46
Generalized gamma	2023.86	2044.06	2033.96
Gompertz	2030.02	2045.16	2037.59
Lognormal	2041.87	2057.01	2049.44
Log Logistic	2029.08	2044.22	2036.65

AIC, Akaike information criteria; BIC, Bayesian information criteria

Supplementary Appendix 2 – Decision modelling methods

Economic model structure

The model used a Markov structure with ten discrete health states reflecting the progression of diabetic retinopathy and its treatment: Observable diabetic retinopathy (bilateral R1, R2 or M1); referable retinopathy (R3 or R4); treated diabetic retinopathy; referable maculopathy (M2); treated diabetic macular oedema; referable retinopathy with referable maculopathy; treated diabetic retinopathy with referable maculopathy; referable retinopathy with treated macular oedema; treated diabetic retinopathy and treated macular oedema; and death (Figure 7). The model operates on a six-monthly cycle, consistent with the shortest screening interval that people with an R2 grade receive in the DES service in Scotland. Treatment for referable disease was modelled as observed in the trial in the "treated diabetic retinopathy" and "treated macular oedema" states. Following completion of expected treatment courses, patients remain in the treated states of the model incurring expected follow-up costs and changes in VA as informed by external literature. A microsimulation approach was used to enable tracking of treatment history and individual patient characteristics, which in turn inform expected costs and utility payoffs within health states.

Deriving transition probabilities

Progression of patients through the model health states was based 6-monthly transition probabilities derived from parametric survival analysis of the LENS trial time to event outcomes.

Observable diabetic retinopathy to referable retinopathy or referable maculopathy

Cause-specific hazards of progression from the baseline observable state to referable retinopathy (R3/R4) with or without referable maculopathy/DMO, and referable maculopathy/DMO alone, were estimated first. For each of these components of the primary progression outcome, proportionality of hazards with respect to treatment allocation was assessed by log-cumulative hazard plots and the Schoenfeld residuals proportional hazards test.

Following this, six standard parametric curves were fitted, with treatment allocation group as a covariate. The resultant curve fits were assessed visually against the observed Kaplan-Meier data, and statistical fit was assess using the Akaike and Bayesian information criteria.

Time to referable maculopathy or treatment for diabetic macular oedema

For progression to referable maculopathy/DMO alone, the lines of the log-cumulative hazards plot do cross early in the follow-up period (Figure 6) but thereafter remain apart and relatively parallel. Furthermore, the p-value from the proportional hazard test is non-significant (Table 3), suggesting insufficient evidence to reject the proportional hazards assumption. Visual inspection of the six parametric curves suggested a good fit for the Weibull, Gompertz and log logistic in the placebo arm (Figure 7) and reasonable fit to the fenofibrate arm (Figure 8). Based on aggregation of the AIC and BIC (Table 4), the Weibull distribution provided the best statistical fit of the six parametric distributions, supporting a diminishing hazard of progression over time, and providing a middle ground in terms of extrapolated outcomes. With the support for the proportional hazards assumption, the Weibull, as a proportional hazards model, was selected for deriving transitions by treatment arm in the base case model (Figure 9). The exponential and log-logistic were assessed as alternatives in scenario analysis.

Figure 6 Log cumulative hazard plot for time to referable maculopathy

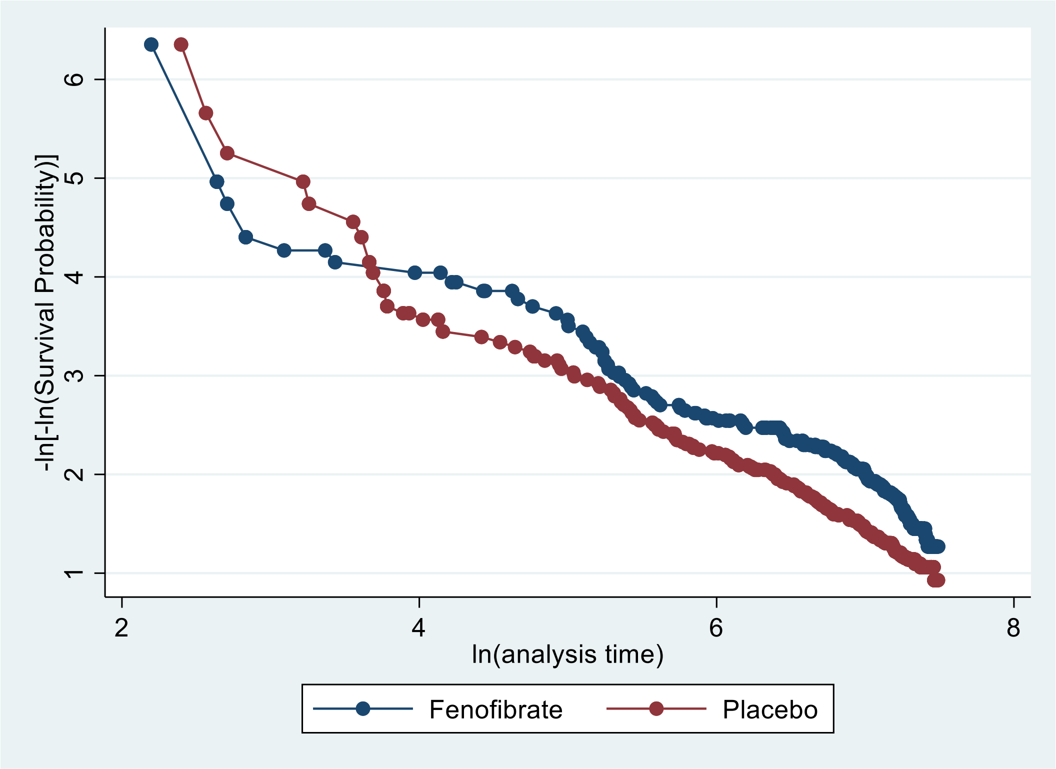

Table 3 Proportional hazards test for time to referable maculopathy with respect to treatment allocation

	Chi-squared	Degrees freedom	P-value
Global test	0.91	1	0.3402

Table 4 Akaike and Bayesian information criteria of different parametric models fitted to time to referable maculopathy

Parametric model	AIC	BIC	Average
Exponential	1794.56	1804.66	1799.61
Weibull	1789.72	1804.87	1797.29
Generalized gamma	1789.78	1809.98	1799.88
Gompertz	1795.87	1811.01	1803.44
Lognormal	1801.23	1816.38	1808.80
Log Logistic	1792.39	1807.53	1799.96

AIC, Akaike information criteria; BIC, Bayesian information criteria

Figure 7 Parametric survival curve fits for time to referable maculopathy in the placebo (standard care) arm

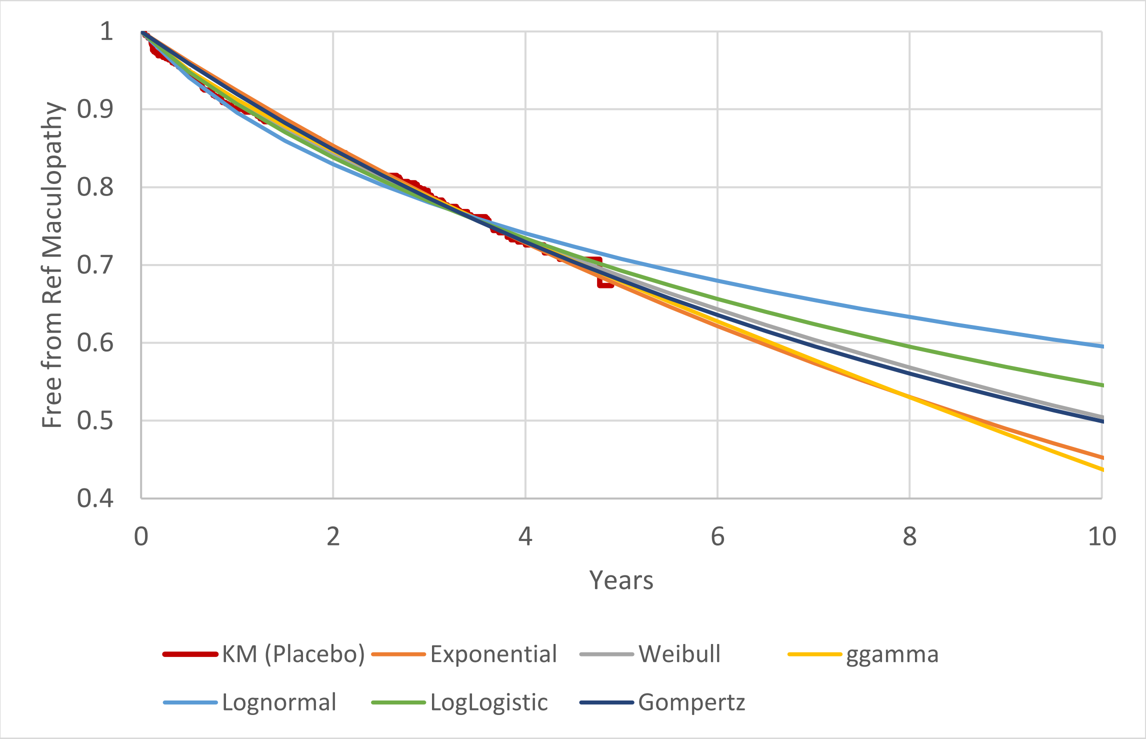

KM, Kaplan Meier; Ref, referable

Figure 8 Parametric survival curve fits for time to referable maculopathy in the fenofibrate arm

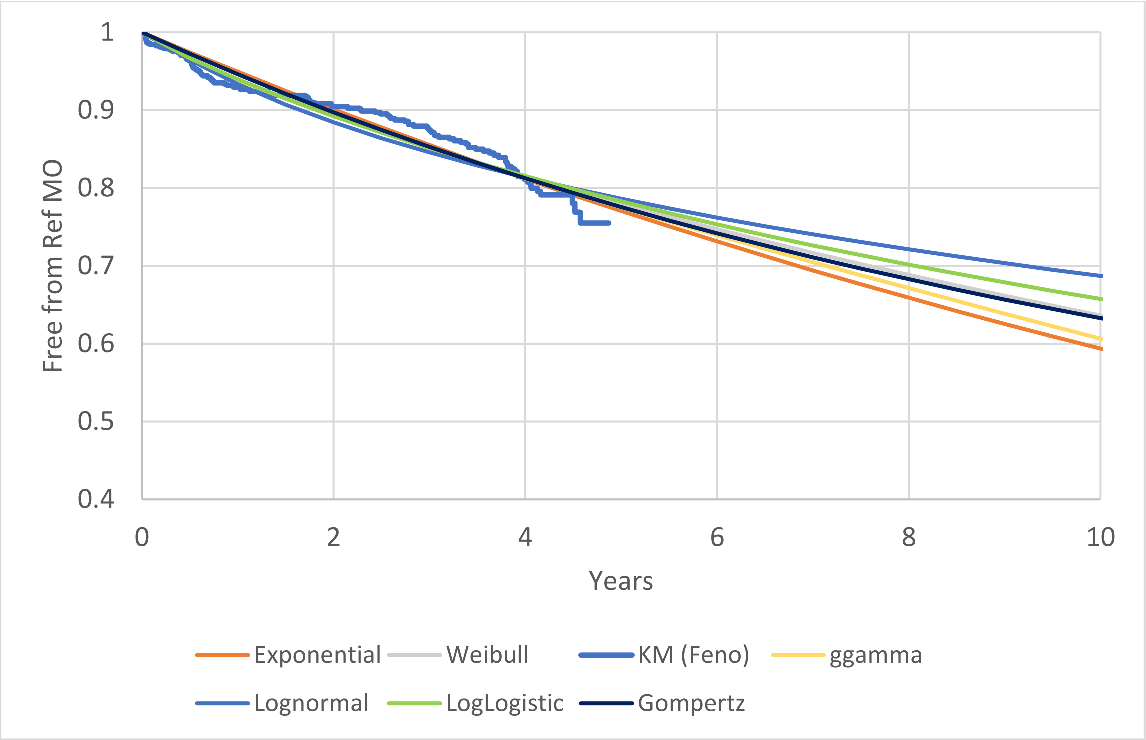

KM, Kaplan Meier; Ref, referable; Feno, fenofibrate

Figure 9 Preferred curve fits for time to referable maculopathy

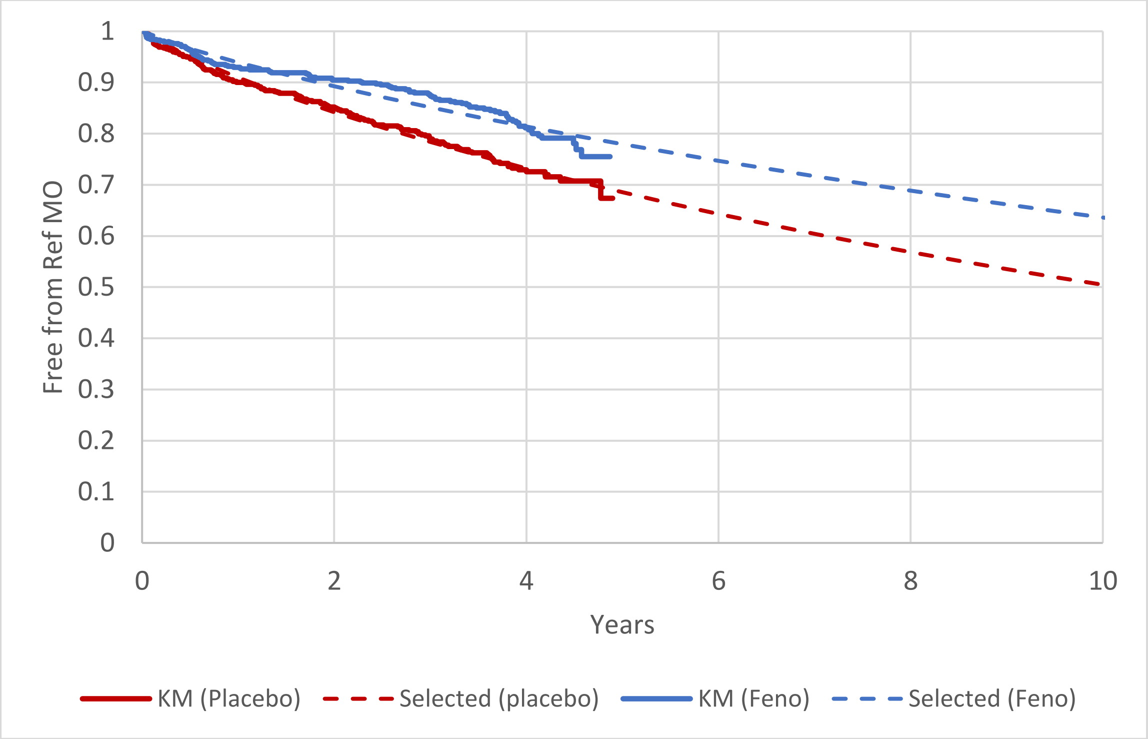

KM, Kaplan Meier; Ref, referable; Feno, fenofibrate

Time to referable retinopathy (R3/R4)

For progression to referable retinopathy (R3/R4) or laser treatment thereof, the same process was followed. The log-log plot (Figure 10) showed the curves to be somewhat intertwined, owing to relatively small number of events to inform hazards. However, the test of Schoenfeld residuals failed to reject the null hypothesis of proportional hazards (Table 5), and so proportional hazard models were prioritised in the selection of curves. Of the available models, the exponential was found to minimise both AIC and BIC (Table 6), provide a reasonable fit to the observed Kaplan-Meier data (Figure 11 and Figure 12), and provide a plausible middle ground in terms of extrapolated outcomes. It was therefore chosen to inform transitions from observable to referable diabetic retinopathy (R3/R4), or treatment thereof, in the base case model (Figure 13). The Gompertz and log-logistic were tested in scenario analyses.

Models of time to referable retinopathy (R3/R4) and referable maculopathy, inclusive of covariates.

The above section shows the alternative parametric curves by treatment allocation group. The microsimulation model, however, was developed with the functionality to include the effects of trial minimisation covariates on the hazards of these progression events. These were informed by parametric survival analysis with the minimisation variables included as covariates. The preferred Weibull distribution was used for progression to referable maculopathy, and the exponential function was used for the transition to referable retinopathy (R3/R4). The AIC and BIC also showed these to provide the best statistical fits, respectively, for the models including covariates. The covariates included categories of age at randomisation (<30; ≥30 <50; ≥50 <70; ≥70 years), type of diabetes (type 1; type 2; other), sex (male; female), HbA1c (<64; ≥64mmol/mol; unknown), renal function (<60; ≥60 mL/min/1.73m2), statin use (Yes; No), baseline retinopathy grade (mild; observable; none), and baseline maculopathy grade (no maculopathy; observable maculopathy). These more complex models were incorporated in the cost-effectiveness model, allowing the predicted progression risks to vary by these characteristics of simulated patients.

Combining cause specific hazards for first progression event

The parameters of the selected Weibull (for time to referable maculopathy or treatment for DMO) and exponential (for time to referable retinopathy (R3/R4) or treatment thereof) functions were used to predict the cause specific hazards by treatment arm for these competing

first progression events by 6-month intervals of follow-up time. These were then combined additively, to estimate the overall hazard of leaving the observable state by treatment allocation group, and then converted into overall time dependent transition probabilities of leaving the state, using the formula:

$p(t) = 1-e^{-rt}$

Where p is the transition probability, t is the model cycle length (= 6 months), and r is the estimated average hazard rate by treatment arm. The overall transition probability for progression, was then distributed between the competing first progression events based on the ratio of their estimated cause specific hazards in each model cycle.

Whilst the analysis of cause specific hazards indicated a significant effect of fenofibrate on the hazard of first progression to referable maculopathy alone (Hazard Ratio, 95% CI: 0.66, 0.51 – 0.85), the effect on first progression to referable retinopathy (R3/R4) was non-significant and directionally favoured the placebo (standard care) arm; Hazard Ratio (95% CI), 1.31 (0.75 – 2.30). This is likely due to small numbers of referable retinopathy (R3/R4) events and is inconsistent with prior evidence suggesting a common treatment effect across the individual retinopathy and maculopathy components of the overall primary progression outcome.[6] Therefore, the overall treatment effect, estimated from Weibull regression of the primary composite outcome (HR, 95% CI: 0.73, 0.58 - 0.92), was applied to both the maculopathy and retinopathy components in the base case analysis. The cause specific hazard ratios were applied in sensitivity analysis.

Figure 10 Log cumulative hazard plot for time to referable retinopathy or laser treatment

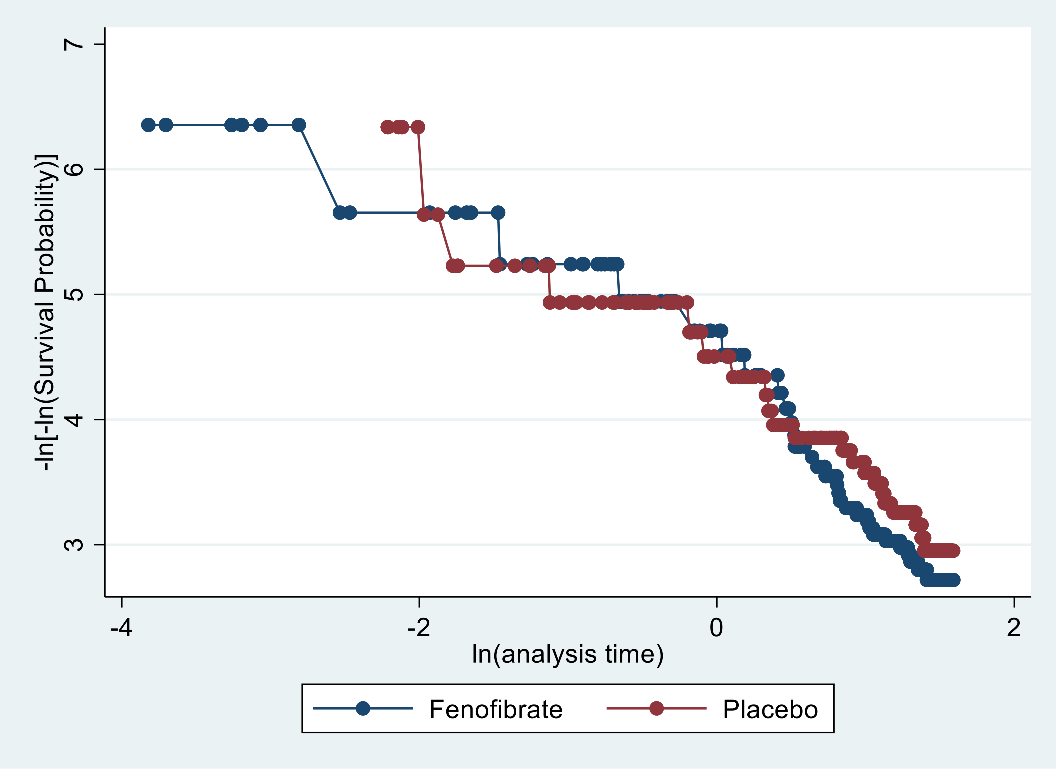

Table 5 Proportional hazards test for time to referable retinopathy (R3/R4) or laser with respect to treatment allocation

	Chi-squared	Degrees freedom	P-value
Global test	0.04	1	0.8375

Table 6 Akaike and Bayesian information criteria of different parametric models for time to referable retinopathy (R3/R4)

Parametric model	AIC	BIC	Average
Exponential	502.77	512.87	507.82
Weibull	504.71	519.85	512.28
Generalized gamma	506.59	526.79	516.69
Gompertz	504.26	519.41	511.84
Lognormal	507.86	523.00	515.43
Log Logistic	504.83	519.98	512.40

AIC, Akaike information criteria; BIC, Bayesian information criteria

Figure 11 Parametric survival curve fits for time to referable diabetic retinopathy in the placebo (standard care) arm

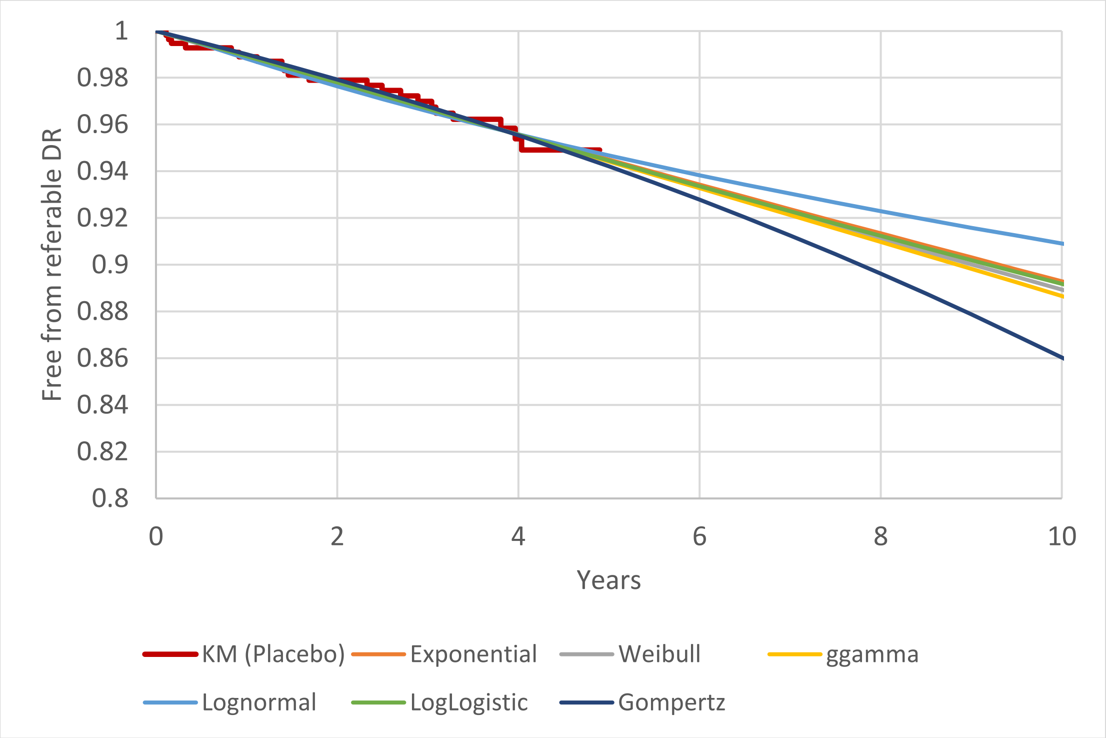

KM, Kaplan Meier; DR, diabetic retinopathy

Figure 12 Parametric survival curve fits for time to referable diabetic retinopathy in the fenofibrate arm

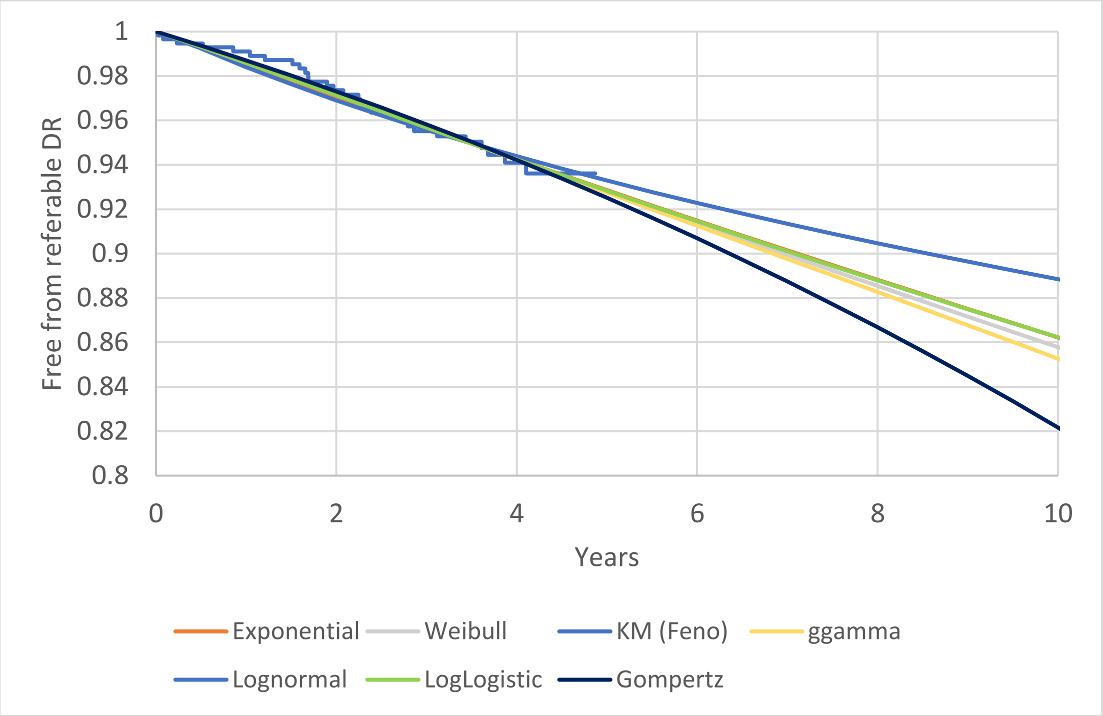

KM, Kaplan Meier; DR, diabetic retinopathy; Feno, fenofibrate

Figure 13 Selected curve fits for time to referable diabetic retinopathy (R3/R4)

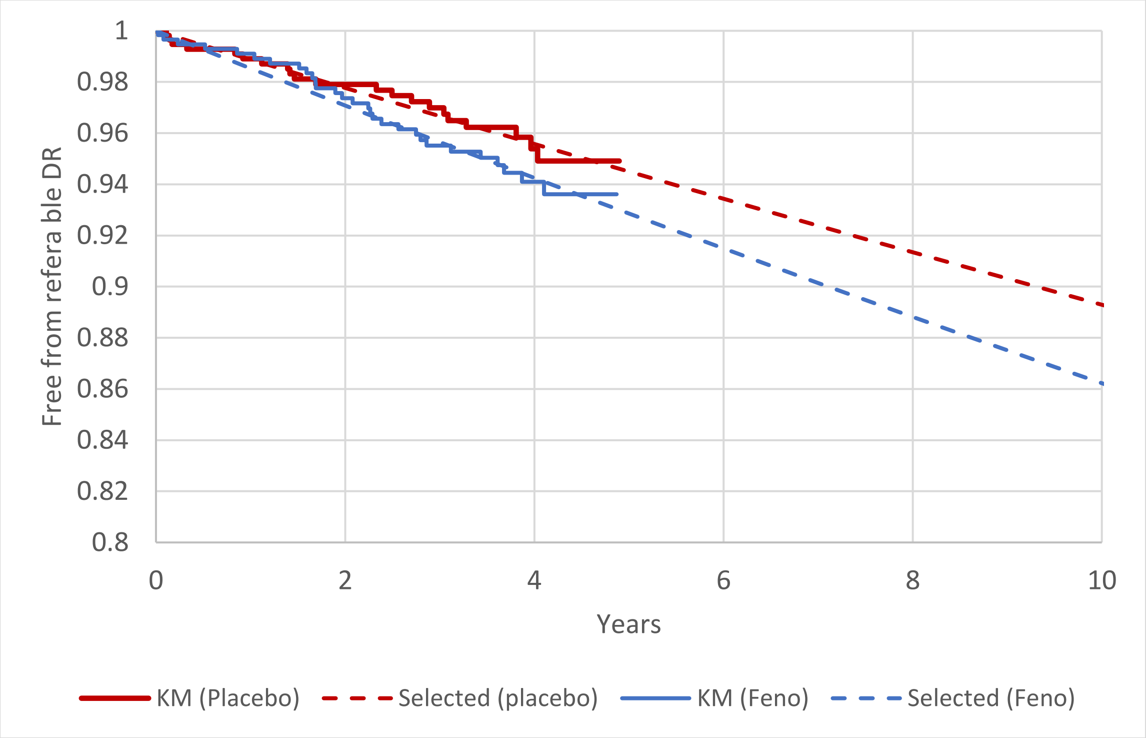

KM, Kaplan Meier; DR, diabetic retinopathy; Feno, fenofibrate

Post progression transition probabilities

Transition probabilities between post-referral states were also informed by LENS trial time to event data, using time of progression to referable retinopathy or referable maculopathy as time zero in the calculation of time at risk. With relatively few events available to inform the distribution of the survivor functions, these event rates were generally assumed to follow exponential distributions, with the effect of fenofibrate applied as a hazard ratio. Nevertheless, the following sections provide the results of proportional hazards assessment and alternative parametric curve fits for each of the required transitions in turn:

- Time from first documented referable maculopathy to treatment for diabetic macular oedema (Figures 14-17 and Tables 7 and 8)
- Time from first documented referable retinopathy (R3/R4) to treatment for referable retinopathy (Figures 18-21 and Tables 9 and 10)
- Time from first document referable maculopathy to referable retinopathy (R3/R4) (Figures 22-25 and Tables 11 and 12)
- Time from first document referable retinopathy (R3/R4) to referable maculopathy. (Figures 26-29 and Tables 13 and 14)

An exception was made for the transition from referable DR (R3/R4) to treatment for referable DR, where independent lognormal distributions were fitted to capture apparent plateauing in the Kaplan Meier data (Figures 18-21 and Tables 9 and 10). Several alternative models were assessed in scenario analysis.

The transitions from referable maculopathy to referable retinopathy, and from referable retinopathy to referable maculopathy, were modelled independently of whether treatment for diabetic macular oedema or treatment for referable retinopathy had occurred, respectively. Similarly, the transitions from referable maculopathy to treatment for DMO, and from referable retinopathy to pan-retinal laser treatment, were modelled independently of each other.

Given the uncertainty surrounding post-progression transition probabilities, further scenario analysis explored the impact of equalising these between the treatment arms, i.e. assuming no effect of fenofibrate on these transition probabilities.

Referable maculopathy to treatment for diabetic macular oedema

Figure 14 Log cumulative hazard plot for referable maculopathy to treatment for diabetic macular oedema

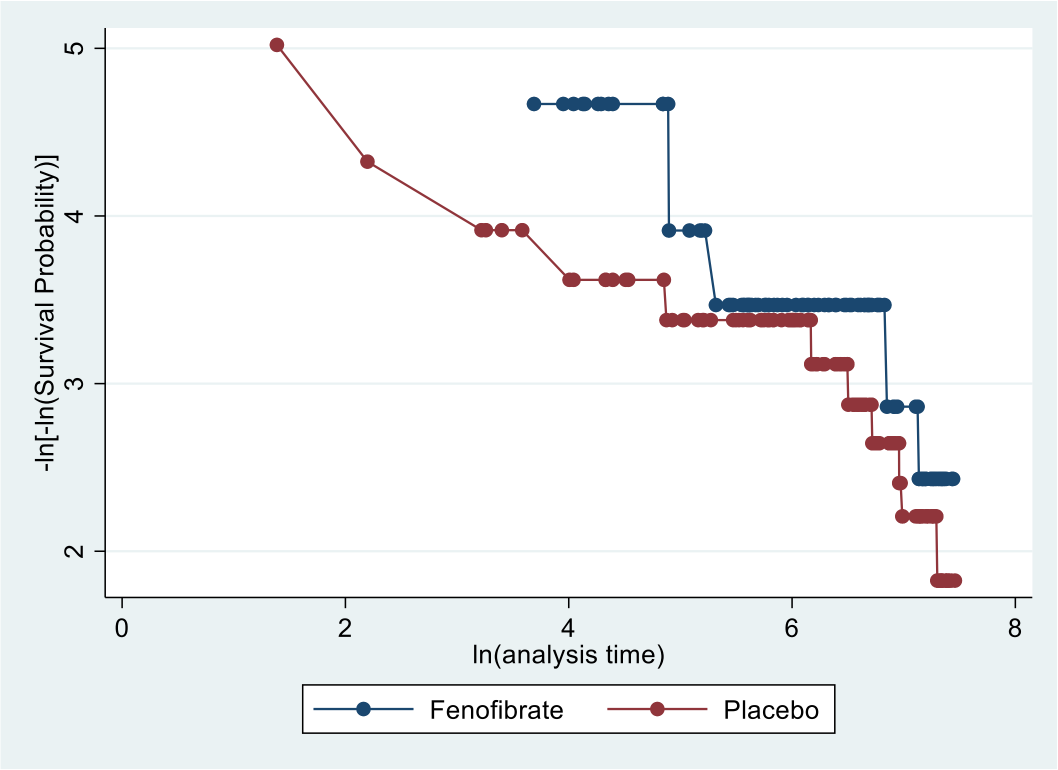

Table 7 Proportional hazards test for referable maculopathy to treatment for diabetic macular oedema with respect to treatment allocation group

	Chi-squared	Degrees of freedom	P-value
Global test	0.00	1	0.9965

Table 8 Akaike and Bayesian information criteria of different parametric models of time from referable maculopathy to treatment for diabetic macular oedema

Parametric model	AIC	BIC	Average
Exponential	169.55	176.68	173.12
Weibull	167.58	178.28	172.93
Generalized gamma	169.41	183.67	176.54
Gompertz	171.41	182.10	176.75
Lognormal	168.42	179.12	173.77
Log Logistic	167.7559	178.4494	173.10

AIC, Akaike information criteria; BIC, Bayesian information criteria

Figure 15 Parametric survival curve fits for referable maculopathy to treatment for diabetic macular oedema in the placebo (standard care) arm

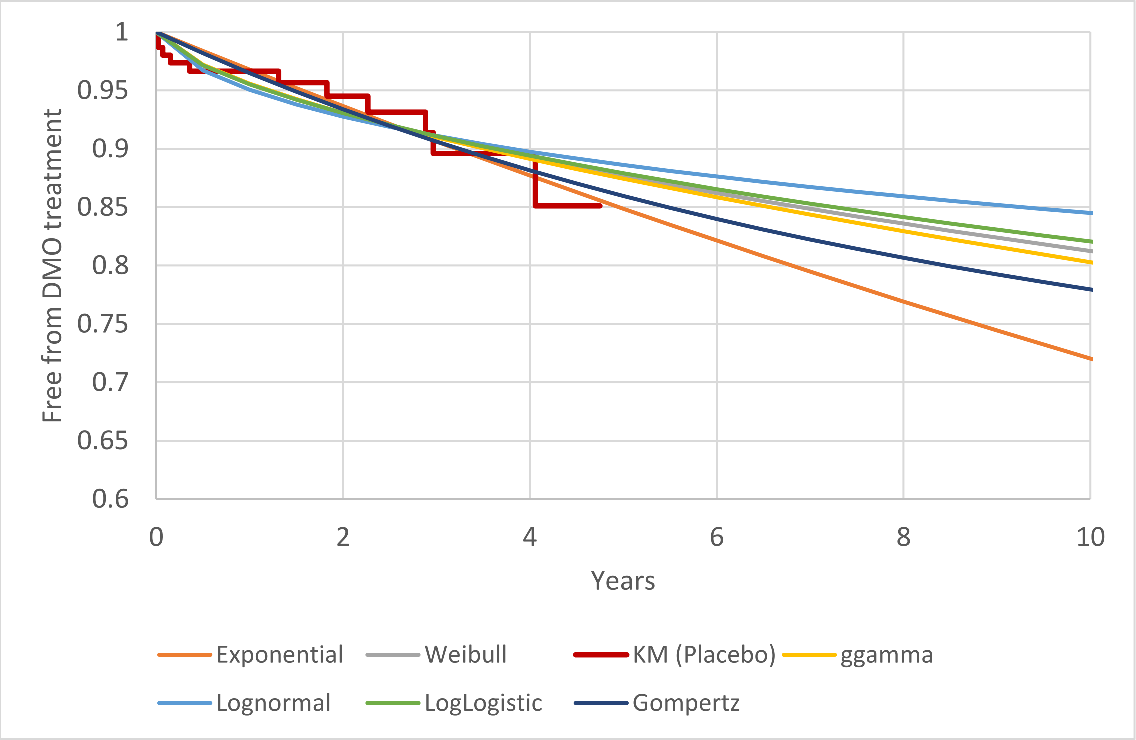

KM, Kaplan Meier; DMO, diabetic macular oedema

Figure 16 Parametric survival curve fits for referable maculopathy to treatment for diabetic macular oedema in the fenofibrate arm

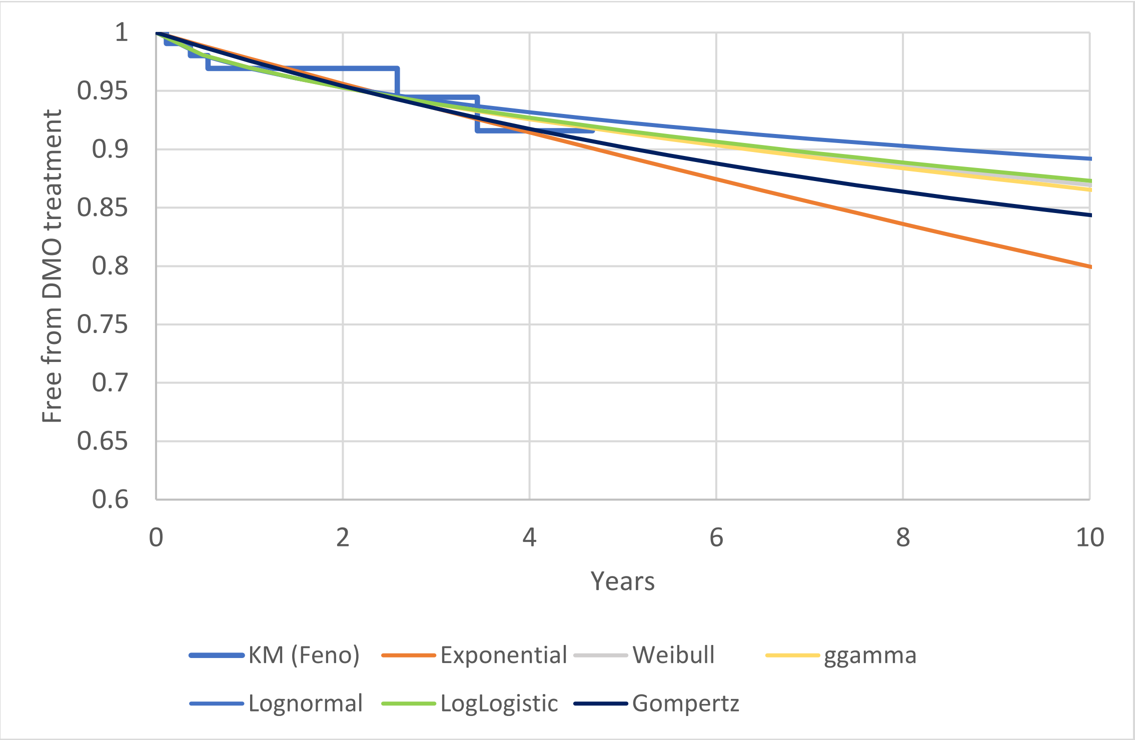

KM, Kaplan Meier; DMO, diabetic macular oedema; Feno, fenofibrate

Figure 17 Selected curve fits for referable maculopathy to treatment for diabetic macular oedema

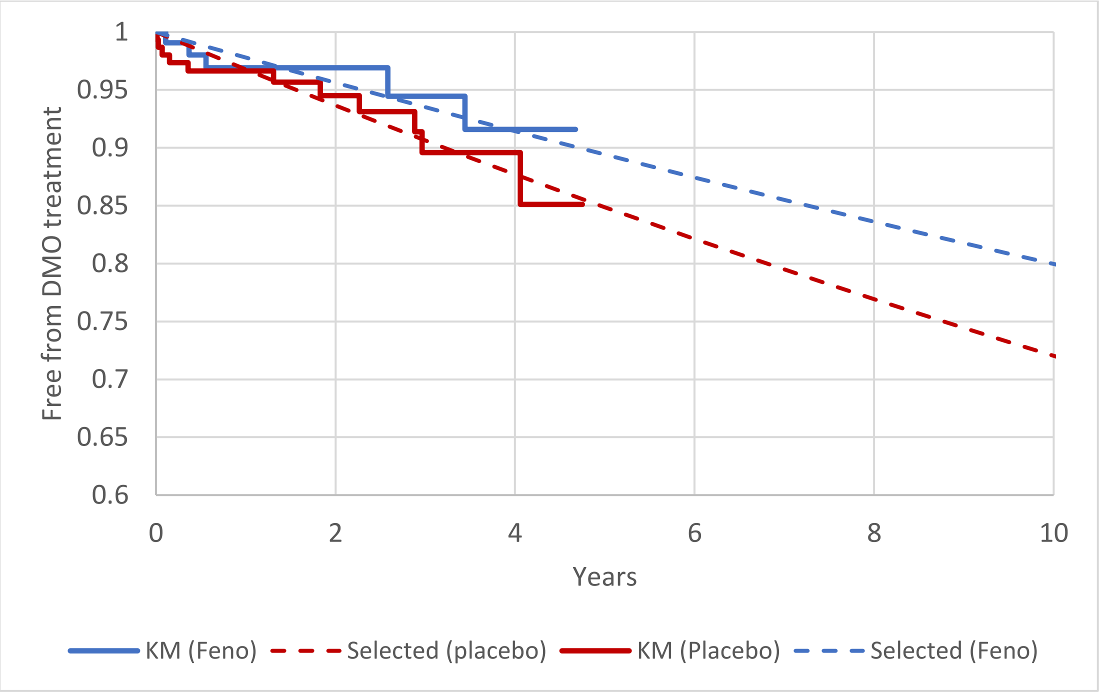

KM, Kaplan Meier; DMO, diabetic macular oedema; Feno, fenofibrate

Referable DR (R3/R4) to treatment for referable DR

Figure 18 Log cumulative hazard plot for referable retinopathy to treatment for referable retinopathy

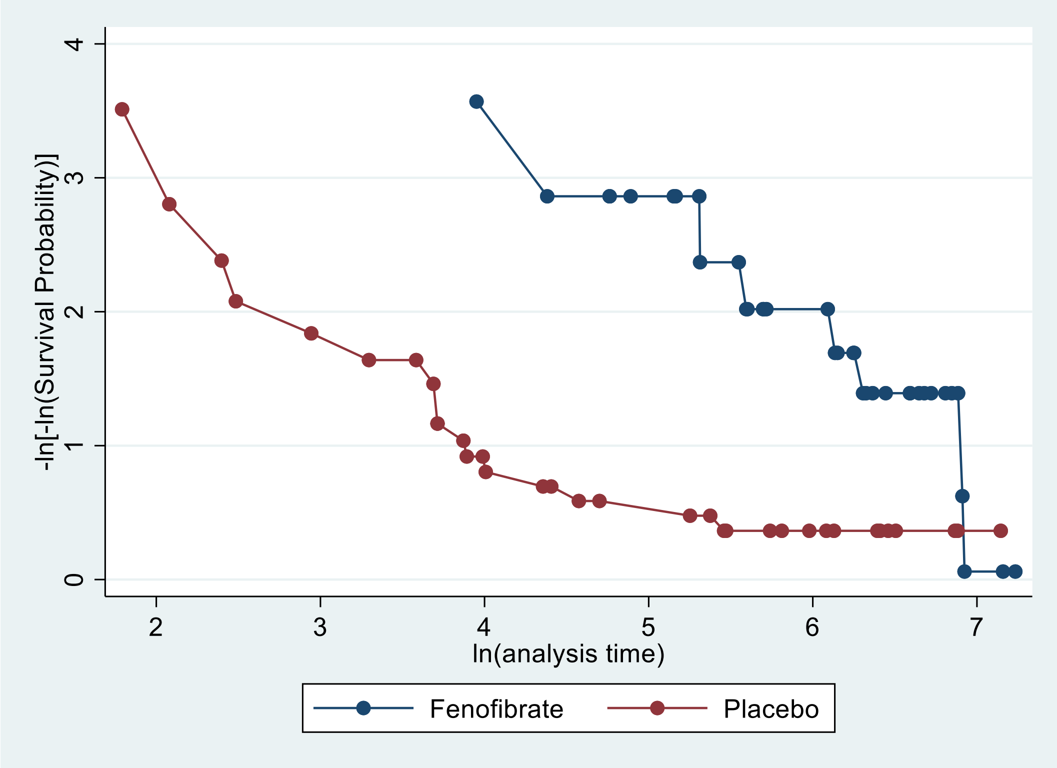

Table 9 Proportional hazards test for referable retinopathy to treatment for referable retinopathy with respect to treatment allocation group

	Chi-squared	Degrees of freedom	P-value
Global test	6.61	1	0.010

Table 10 Akaike and Bayesian information criteria of parametric models of time from referable retinopathy to treatment for referable retinopathy, fitted independently by treatment arm

	Placebo			Fenofibrate		
**Parametric model**	**AIC**	**BIC**	**Average**	**AIC**	**BIC**	**Average**
Exponential	122.80	124.33	123.56	51.41	53.04	52.22
Weibull	108.74	111.79	110.26	53.14	56.41	54.77
Generalized gamma	NC	NC	-	54.91	59.82	57.37
Gompertz	96.44	99.49	97.97	52.91	56.19	54.55
Lognormal	104.20	107.25	105.73	53.56	56.83	55.20
Log Logistic	105.95	109.01	107.48	53.40	56.68	55.04

AIC, Akaike information criteria; BIC, Bayesian information criteria; NC, non-convergence of model

Figure 19 Parametric survival curve fits for time from referable retinopathy to treatment for diabetic retinopathy in the placebo (standard care) arm

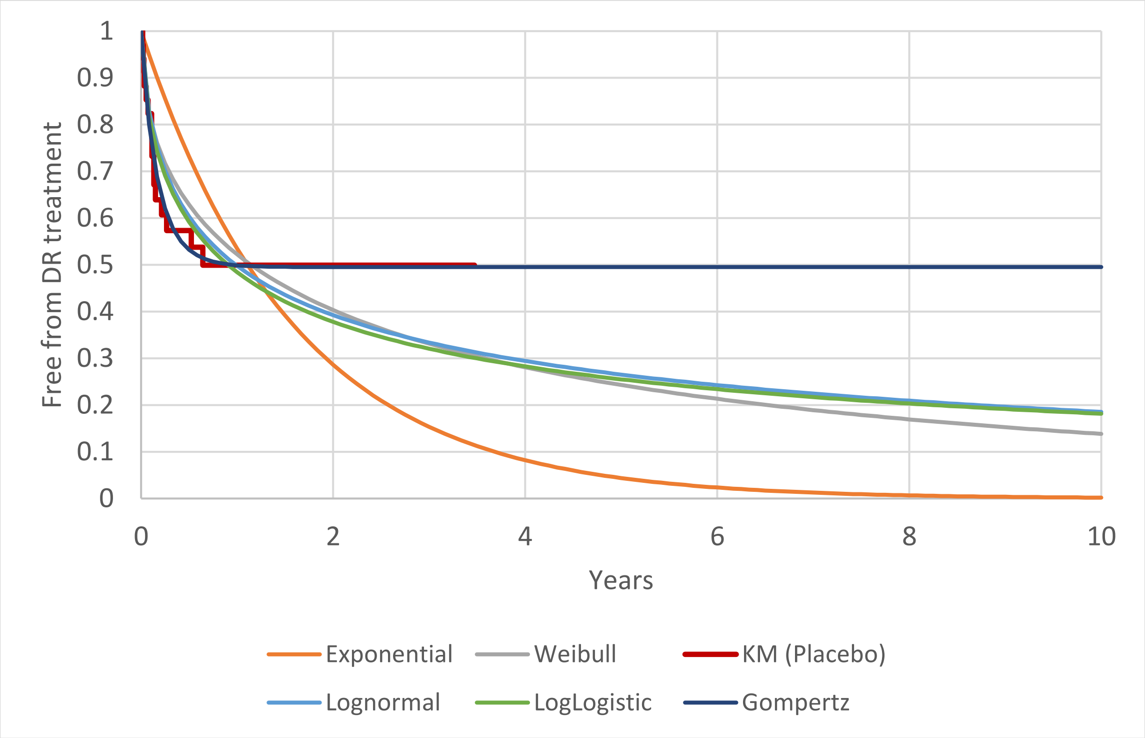

KM, Kaplan Meier; DR, diabetic retinopathy

Figure 20 Parametric survival curve fits for time from referable retinopathy to treatment for diabetic retinopathy in the fenofibrate arm

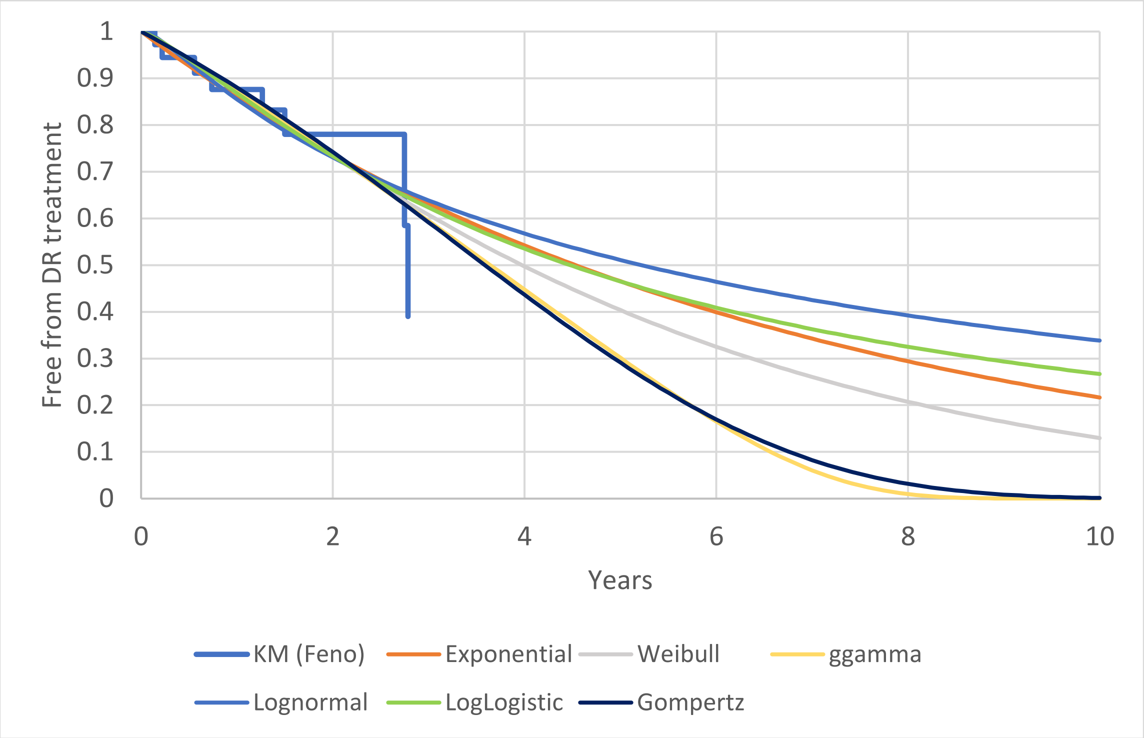

KM, Kaplan Meier; DR, diabetic retinopathy; Feno, fenofibrate

Figure 21 Selected curve fits for time from referable retinopathy (R3/R4) to treatment for diabetic retinopathy

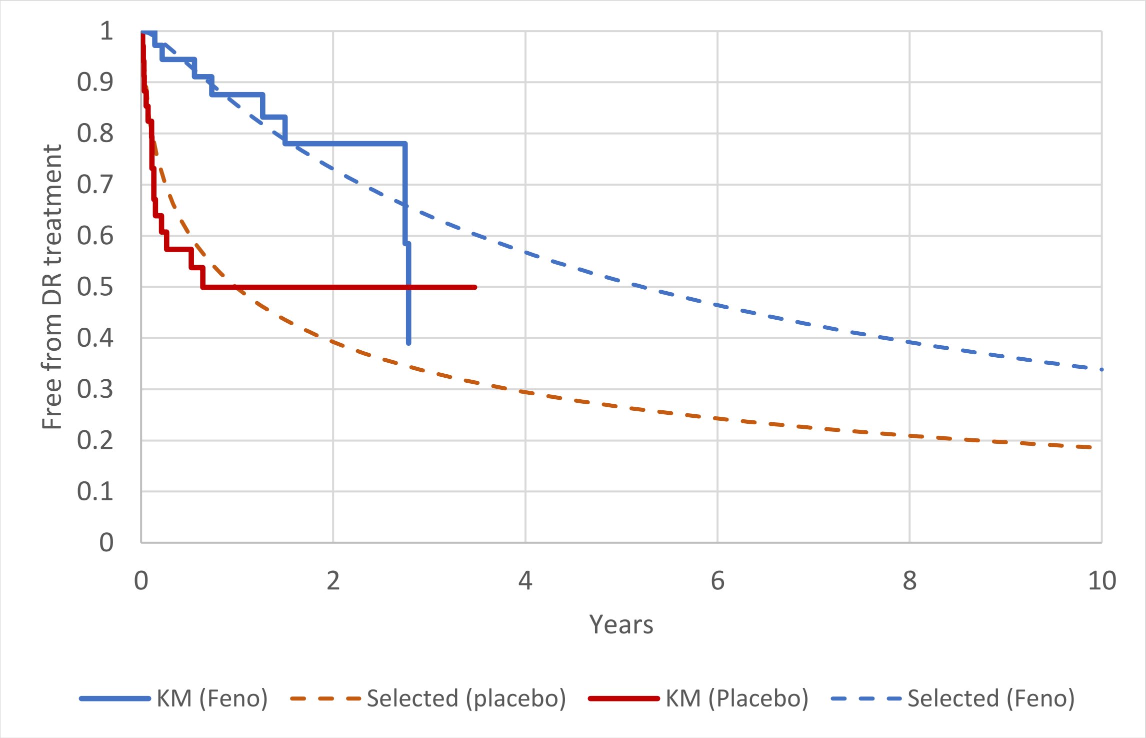

KM, Kaplan Meier; DR, diabetic retinopathy; Feno, fenofibrate

Referable maculopathy to referable diabetic retinopathy (R3/R4)

Figure 22 Log cumulative hazard plot for time from referable maculopathy to referable diabetic retinopathy

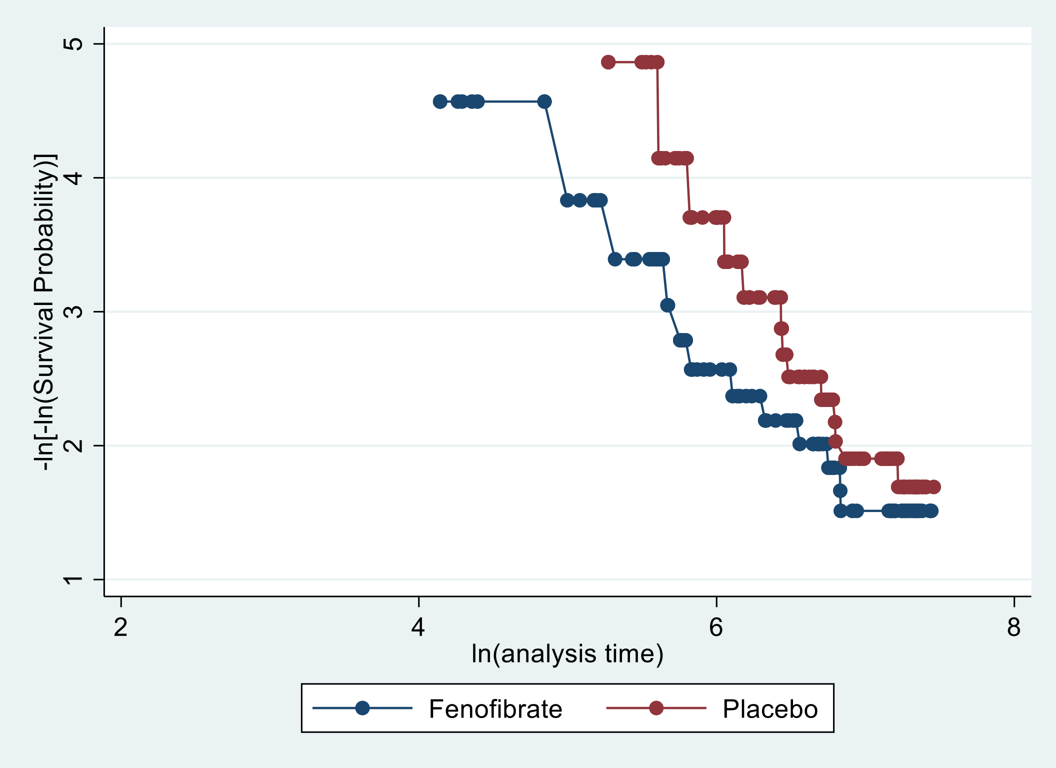

Table 11 Proportional hazards test for referable maculopathy to referable diabetic retinopathy by treatment allocation group

	Chi-squared	Degrees of freedom	P-value
Global test	1.88	1	0.170

Table 12 Akaike and Bayesian information criteria of different parametric models of time from referable maculopathy to referable diabetic retinopathy (R3/R4)

Parametric model	AIC	BIC	Average
Exponential	192.83	199.85	196.34
Weibull	193.31	203.85	198.58
Generalized gamma	190.38	204.44	197.41
Gompertz	194.77	205.31	200.04
Lognormal	190.33	200.87	195.60
Log Logistic	192.60	203.14	197.87

AIC, Akaike information criteria; BIC, Bayesian information criteria

Figure 23 Parametric survival curve fits for time from referable maculopathy to referable diabetic retinopathy in the placebo (standard care) arm

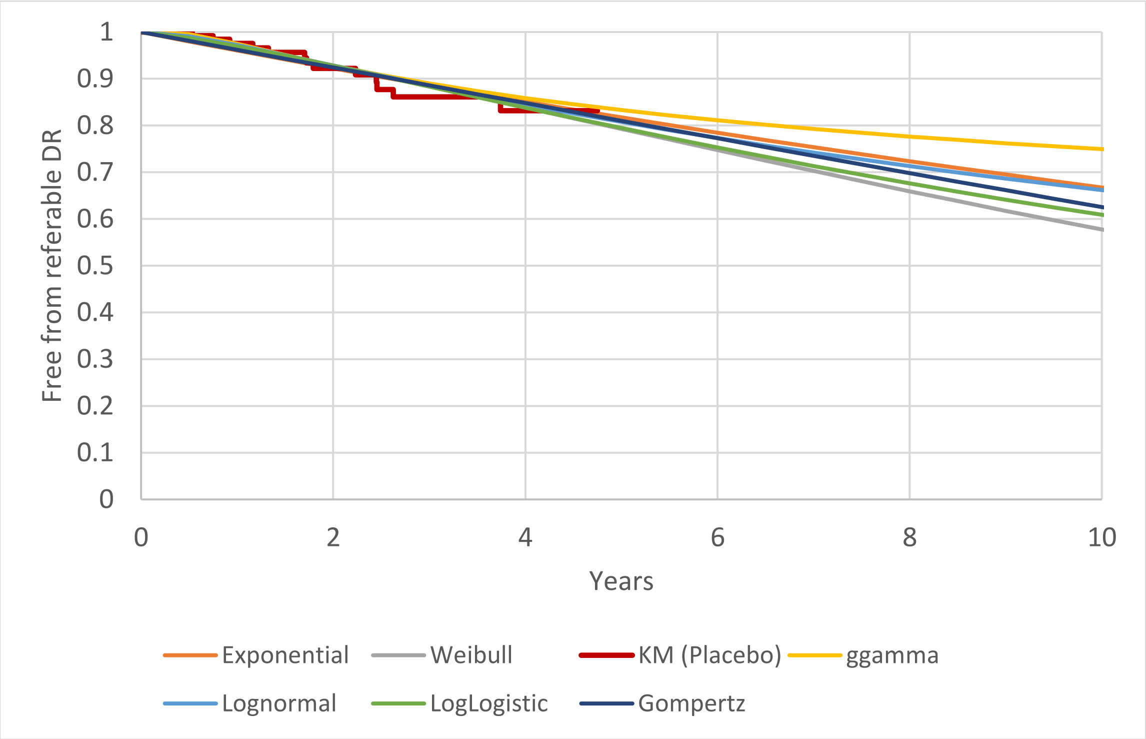

KM, Kaplan Meier; DR, diabetic retinopathy

Figure 24 Parametric survival curve fits for time from referable maculopathy to referable diabetic retinopathy in the fenofibrate arm

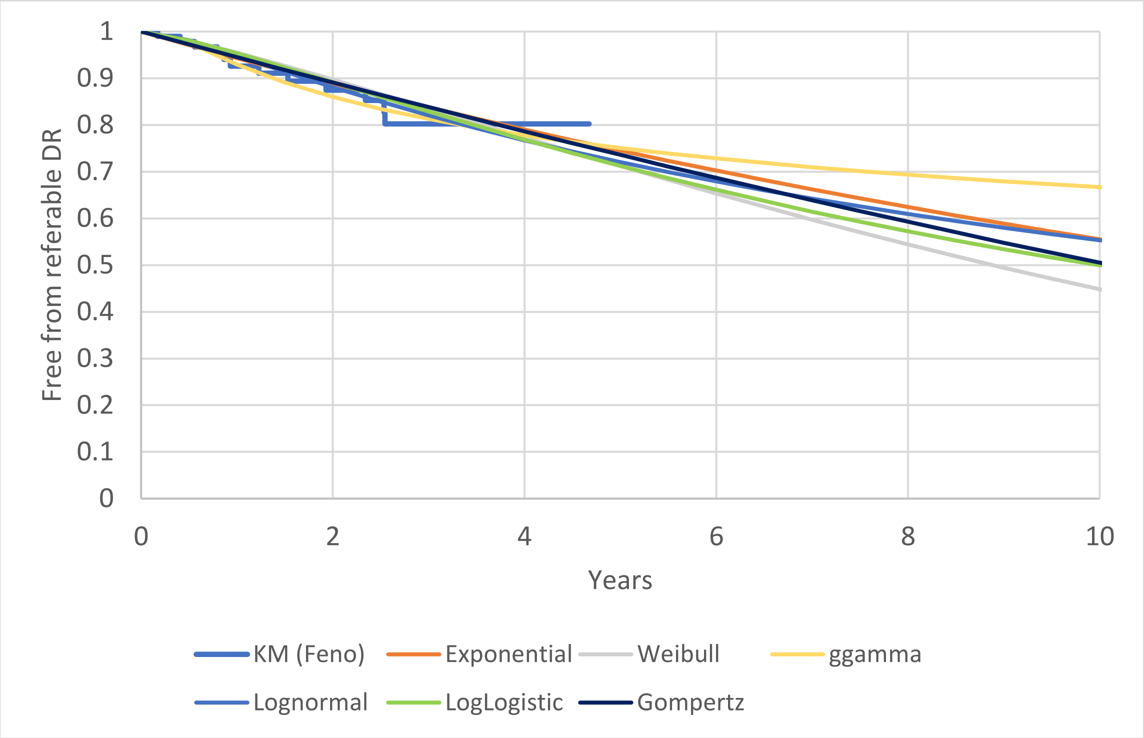

KM, Kaplan Meier; DR, diabetic retinopathy, Feno, fenofibrate

Figure 25 Parametric survival curve fits for time from referable maculopathy to referable diabetic retinopathy

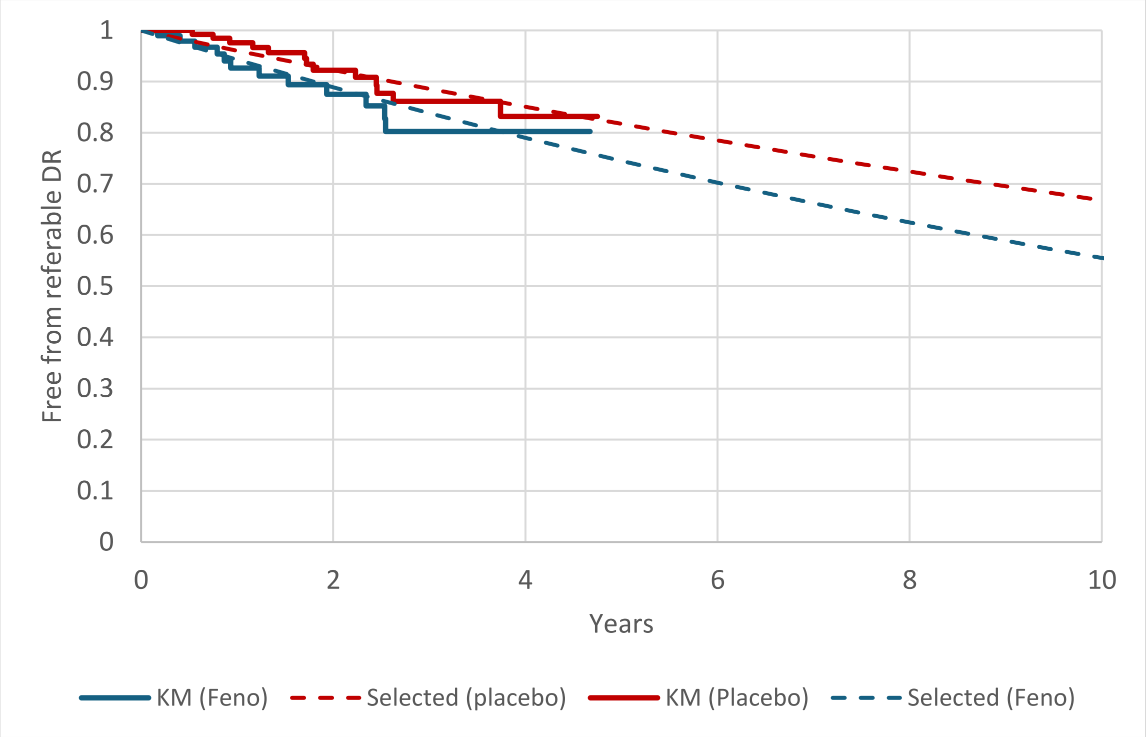

KM, Kaplan Meier; DR, diabetic retinopathy; Feno, fenofibrate

Referable diabetic retinopathy (R3/R4) to referable maculopathy

Figure 26 Log cumulative hazard plot for time from referable retinopathy (R3/R4) to referable diabetic maculopathy

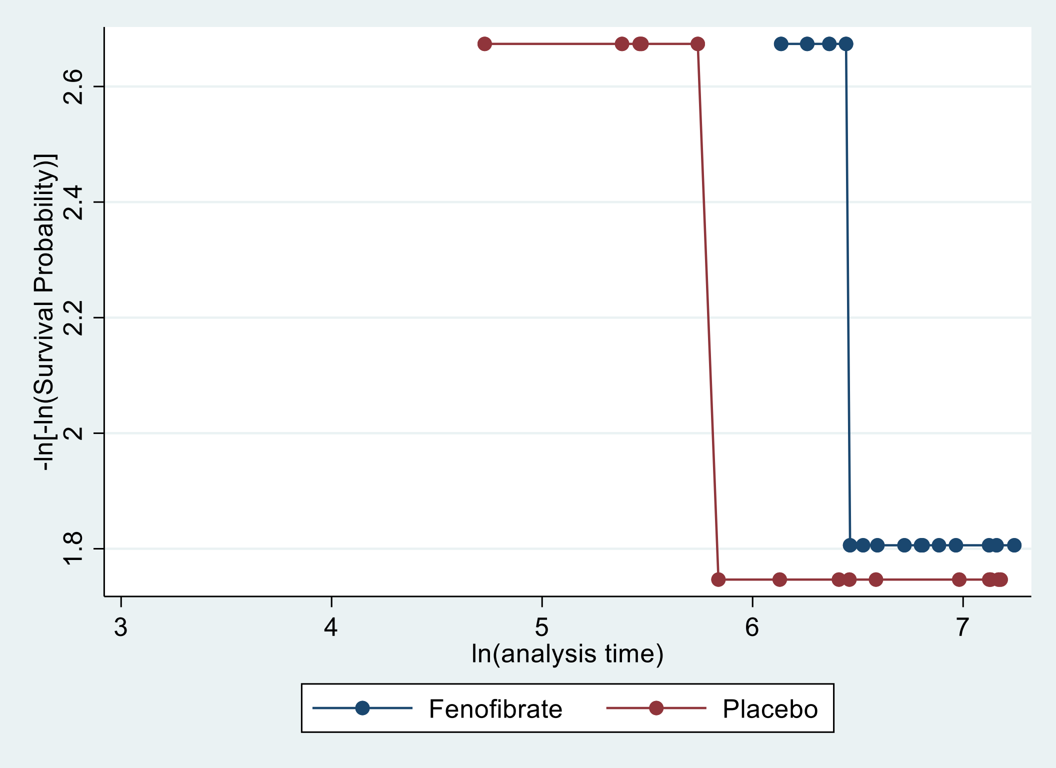

Table 13 Proportional hazards test for time from referable retinopathy (R3/R4) to referable diabetic maculopathy, with respect to treatment allocation group

	Chi-squared	Degrees of freedom	P-value
Global test	2.44	1	0.118

Table 14 Akaike and Bayesian information criteria of different parametric models of time from referable diabetic retinopathy R3/R4) to referable maculopathy

Parametric model	AIC	BIC	Average
Exponential	35.15	38.37	36.76
Weibull	37.12	41.95	39.54
Generalized gamma			
Gompertz	36.97	41.80	39.38
Lognormal	36.31	41.14	38.72
Log Logistic	192.60	203.14	197.87

AIC, Akaike information criteria; BIC, Bayesian information criteria

Figure 27 Parametric survival curve fits for time from referable retinopathy to referable maculopathy in the placebo (standard care) arm

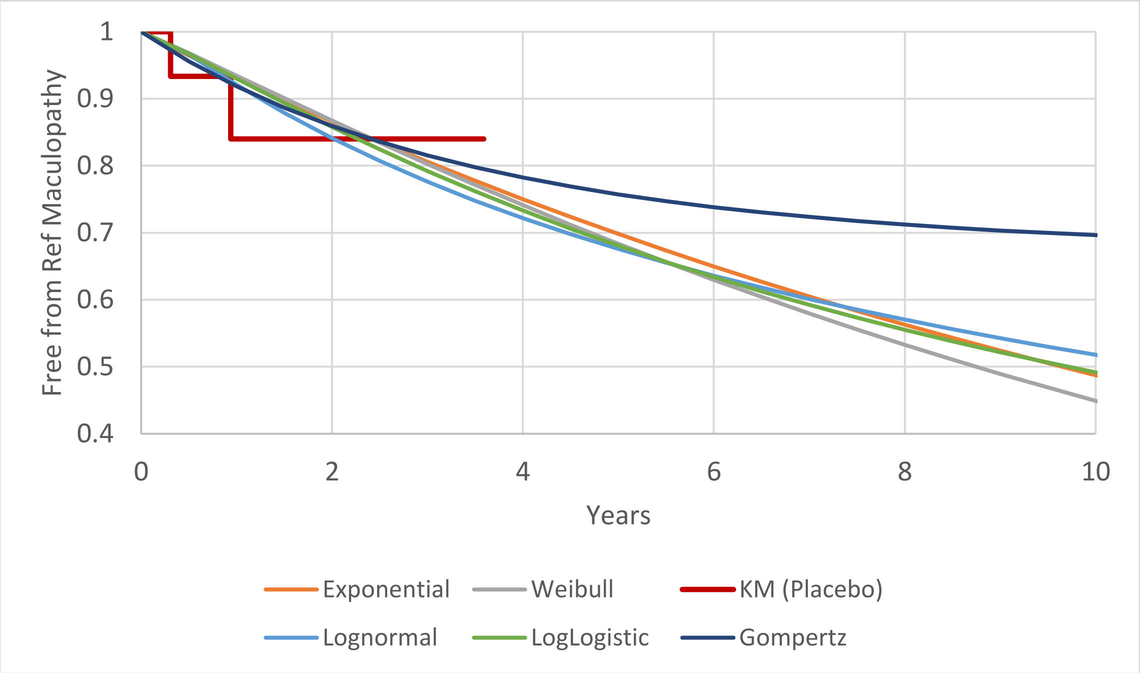

KM, Kaplan Meier; Ref, referable

Figure 28 Parametric survival curve fits for time from referable retinopathy to referable maculopathy in the fenofibrate arm

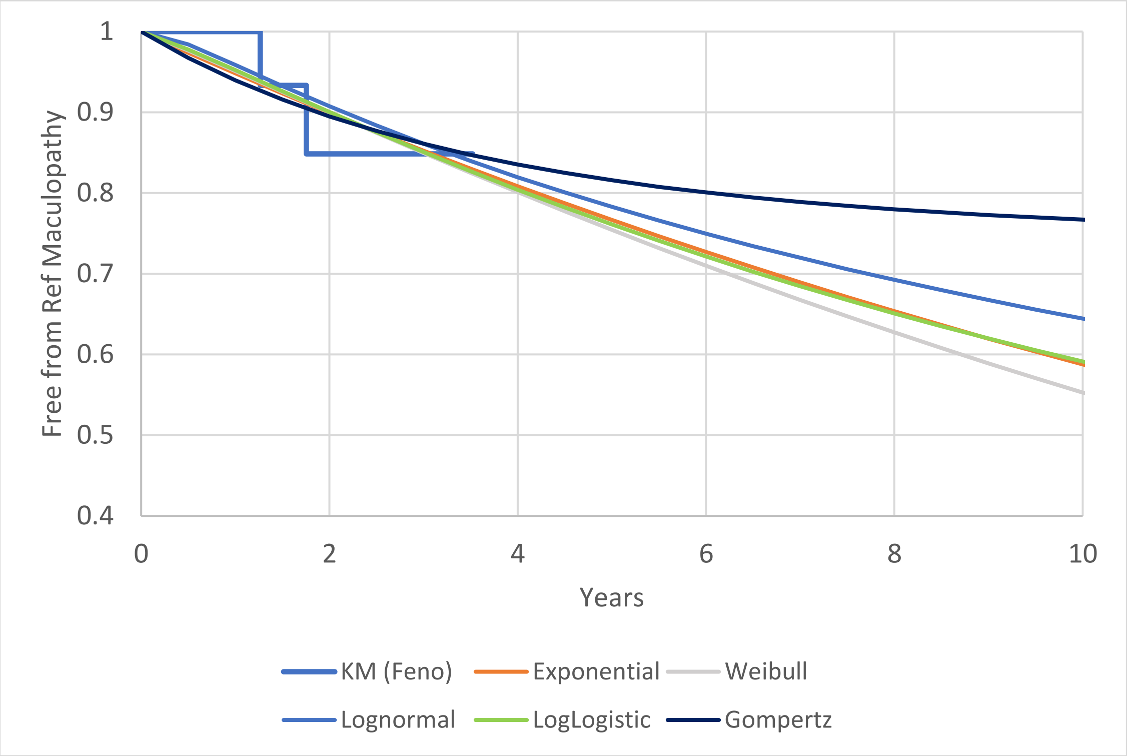

KM, Kaplan Meier; Ref, referable; Feno, fenofibrate

Figure 29 Selected Parametric survival curve fits for time from referable retinopathy to referable maculopathy

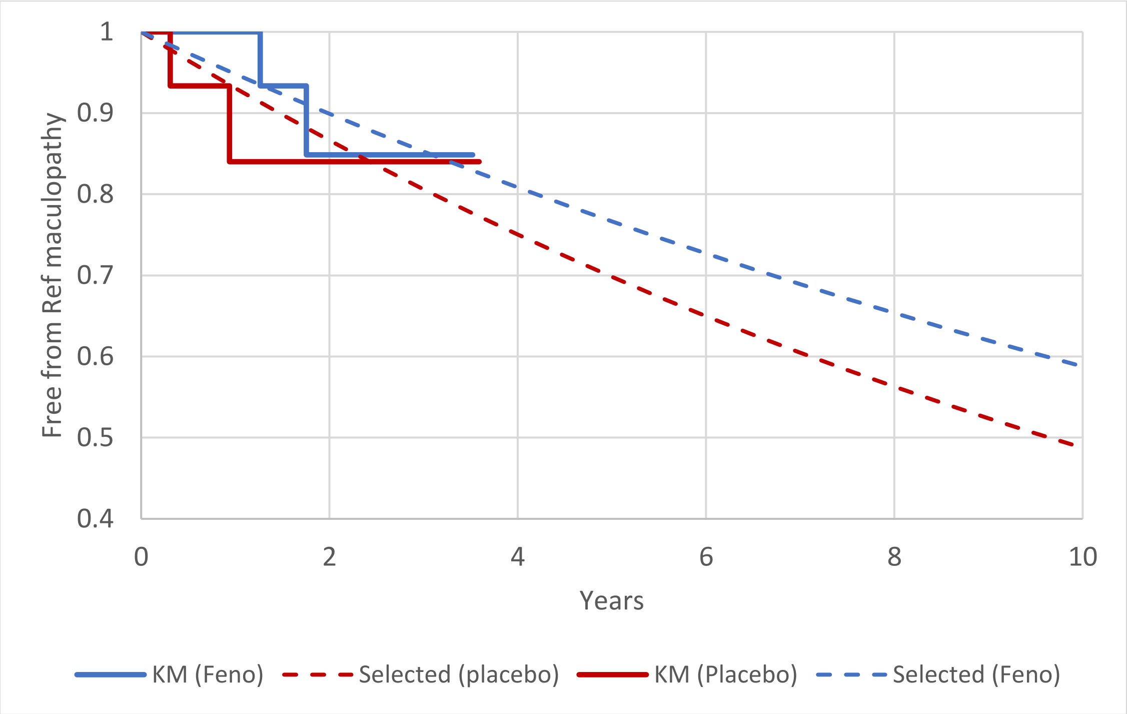

KM, Kaplan Meier; Ref, referable; Feno, fenofibrate

Health state utility impact of progression

Analysis of the EQ-5D data from LENS indicated negligible effects of treatment allocation on HRQoL over the trial follow-up period.[7] This may be due to the relatively small absolute differences in number with progression over follow-up. Nevertheless, it is logical to assume that progression of retinopathy to sight threatening stages has a negative impact on HRQoL due to anxiety and the additional monitoring and treatment burden of developing referable disease. Therefore, the LENS trial data were pooled across the treatment arms, and mixed models for repeated measures were used to assess the effect of progression on health state utility. Alternative models were assessed with a random effect for individual, and fixed effects for the various types of progression event, time from baseline, baseline EQ-5D score, and other selected baseline covariates. Based on Akaike and Bayesian information criteria (AIC and BIC), the preferred model included fixed effects for baseline EQ-5D, baseline age, time from baseline, and any referable diabetic eye disease or any treatment thereof. EQ-5D data were available for n=1,142 (99%) at baseline, n=908 (79%) at two years, and n=854 (74% and study exit). There was n=346 (20%) post-progression observations during follow-up. The mixed model for repeated measures assumes data are missing at random, conditional on the covariates included in the analysis model.

Table 15 Available EQ-5D data by time point

Time point	Number (%)	Mean (SD)
Baseline	1142 (99%)	0.811 (0.226)
Two years	908 (79%)	0.758 (0.261)
Study exit	854 (74%)	0.745 (0.263)

Modelled long-term changes in visual acuity and impact on quality of life

With relatively few patients in the LENS trial reaching the stage where treatment was indicated for PDR or DMO, there was insufficient data to inform expected visual acuity outcomes following treatment. Therefore, external literature was consulted for this purpose, with a focus on pragmatic trials of diabetic retinopathy treatments and NHS cohort studies. Expected changes to VA following treatment initiation, were applied to observed best corrected visual acuities at the latest available assessment prior to treatment with anti-VEGF or panretinal laser

photocoagulation (PRP). Based on findings from a real-world NHS cohort study, ant-VEGF injections were assumed to maintain VA at pre-treatment levels to two years post-treatment.[4] Beyond two years, it was assumed that VA in treated eyes deteriorates slowly at the level observed during the long-term follow-up (under routine management) of patients enrolled in the Protocol T extension study.[8] This reported a mean reduction of 4.7 ETDRS letters (1.6 ETDRS letters per year) between two years and five years of follow-up.

For eyes with proliferative diabetic retinopathy treated with PRP, VA changes were modelled to align with those reported in a retrospective NHS cohort study.[9] Fu et al. studied 2,336 eyes with stable proliferative diabetic retinopathy following PRP and reported a 50% probability of losing ≥5 ETDRS letters or more by 3.32 years. A mean (SD) reduction of 14.2 (13) ETDRS letters was observed at this event. A six-monthly probability of losing ≥5 letters following PRP was estimated from this data and applied in the model, with the number of letters lost drawn from a gamma distribution with mean (SD) matching that reported by Fu et al.

Given uncertainty regarding the linkage of treatment to the individual eyes, it was conservatively assumed that post-treatment VA changes apply only to the WSE at treatment initiation. Sensitivity analysis assessed the impact of applying VA changes to the BSE or both eyes.

With relatively few patients in the LENS trial reaching more advanced stages of referable disease requiring treatment, the estimated utility decrement associated with referable disease (estimated from LENS trial EQ-5D data) was assumed to capture the effect on quality of life of early referable disease (prior to treatment). Longer-term utility impacts following initiation of retinal treatment were therefore modelled through expected impacts on visual acuity linked with external literature on health state utility by visual acuity status. Focused searches were used to identify studies reporting on the relationship between visual acuity and health state utility values in people with diabetes. The focus was on identifying studies of patient reported health status using health related quality of life instruments for which health state utility weights, suitable for the calculation of QALYS, are available. The selected source reported EQ-5D and VFQ-25 derived values based on application of the UK general population valuation tariff to EQ-5D-3L response data, and the VFQ-UI to VFQ-25 response data, collected from 1,320 patients enrolled in four trials of intraretinal aflibercept injections for macular oedema.[10] Brazier et al. reported predicted EQ-5D and VFQ-UI values based on

regression models accounting for BCVA in the best and worst seeing eye over time. The estimated effects from the authors preferred ordinary least squares model of EQ-5D values was chosen for incorporation in the model base case. This can be considered conservative, as there are recognised limitations of the EQ-5D for picking up changes in quality of life due to changes in VA. The VFQ-UI can be considered a more sensitive measure for this purpose, which was tested in sensitivity analysis.

Modelled resource use assumptions

Six-monthly background health care resource use costs of LENS trial participants, inclusive of biochemistry monitoring, hospital activity in specialties of interest (excluding ophthalmology and diabetic retinopathy treatment costs), and relevant community prescribed medicines, were analysed by treatment allocation group and health state occupancy. General estimating equations (GEEs) were used to estimate differences in these costs by health state and/or treatment allocation. Whilst the estimated mean difference in these six-monthly health care costs directionally favoured fenofibrate, the difference was not statistically significant (-113; 95% CI: -275 to 49). Similarly, no statistically significant differences in background health care costs were identified by diabetic retinopathy progression status. Therefore, they were conservatively equalised across treatment arms and health states in the economic model and assumed not to influence modelled cost-effectiveness.

A similar analysis was performed to estimate screening costs by model health state, with the GEE regression including health state indicators for referable maculopathy, treated maculopathy, referable retinopathy, and treated retinopathy. Screening costs were assumed to stop for those developing referable retinopathy (R3/R4) or macular oedema requiring treatment, since care and follow-up would move to hospital eye services for these patients. Cost of prescribing fenofibrate were assumed to be negligible and absorbed in the context of ongoing diabetes management and monitoring. Sensitivity analysis assessed the impact of including an extra GP appointment to initiate treatment and an additional creatinine test at 1-2 months to assess kidney function following initiation of treatment.

Those being referred for treatment of DMO were assumed to require 6.3 injections in year 1 and 2.9 injections in year two, based on data from a contemporary NHS cohort study under routine practive.[9] In years 3-5, they were assumed to continue undergoing six-monthly

outpatient monitoring in the hospital eye service and receive a further 2.45 injections over this time-period. This was based on the ratio of the median number of aflibercept injections in years 3-5 to the median number in years 1-2 of the Protocol T study (=4/15),[118] multiplied by the mean number of injections over two years in the DRAKO cohort study (9.2).[9] No further treatment was assumed after five years, but patients continued to be monitored annually in the hospital outpatient eye service based on clinical expert opinion.

Modelled resource use assumptions for retinal treatment were aligned with the sources of evidence used to inform expected post-treatment changes in visual acuity and were validated by clinical expert opinion. Those referred for severe background retinopathy (R3) were assumed to require six-monthly monitoring via face-to-face ophthalmology outpatient appointments. Patients progressing to PRP for proliferative DR were assumed to require on average of 2.77 treatment sessions (applied in the cycle of treatment initiation),[12] followed by six-monthly outpatient monitoring in hospital eye services. Beyond three years, it was assumed that patients would be discharged from the hospital eye services back to the screening programme.

Microsimulation model analysis

Monte Carlo simulation was used to propagate the passage if simulated patients through the model one at time. Simulated patients were generated by bootstrap resampling of the baseline characteristics of LENS trial participants, retaining correlations between characteristics: Age group (<30; ≥30 <50; ≥50 <70; ≥70 years), sex, type of diabetes (type 1, type 2, other), HbA1c category (<64; ≥64mmol/mol; unknown), estimated glomerular filtration rate category (<60; ≥60 mL/min/1.73m2), statin use, baseline maculopathy grade (none, observable), baseline retinopathy grade (none, mild, observable). These characteristics were combined with the parameter estimates of the fitted survival regressions for the components of the first documented progression (referable maculopathy or treatment for diabetic macular oedema; referable retinopathy (R3/R4) or treatment thereof), to generate individualised transition probabilities for these events. Following initial progression, transitions followed average estimates by treatment allocation group, due to their being too few events to inform transitions by individual characteristics. Assessment of modelled costs and outcomes over the primary ten-year time horizon determined that incremental costs stabilised (+/-£4) and incremental QALYs stabilised within +/- 0.001 across 10 batches of 500,000 simulations. With respect to

probabilistic sensitivity analysis, a pragmatic compromise was made to use 1,000 draws to represent second order uncertainty in the parameter estimates. However, the computational burden was too great to enable 1,000 x 500,000 runs, and so 1,000 x 50,000 was used. Whilst point estimates of the ICER fluctuated somewhat based on these numbers, the decision uncertainty (probabilities of cost-effectiveness at key thresholds) was found to be stable.

Heterogeneity in the cost-effectiveness was explored for key subgroups with enough participants to accurately inform subgroup specific progression risks: type of diabetes, baseline HbA1c status; and baseline maculopathy grade. Whilst these analyses reflect subgroup specific risks of progression, they assume the same overall effects of fenofibrate on progression risks. This is supported by evidence from the clinical effectiveness analysis, which supported a consistent treatment effect across all prespecified subgroups.[7]

Model validation

Consistency and validity were assessed by careful checking of formulas, running scenarios to equate costs and QALYs between treatment arms, checking that changes in model parameter inputs produced results in line with expectation, and by assessing the fit of modelled clinical progression outcomes against the summary Kapan-Meier curves from the LENS trial. These projections (modelled outcomes), provided as supplementary figures 1 and 2, showed a good fit with the observed data and make clear the ten-year extrapolation of outcomes. Recognising the uncertainty inherent in any extrapolation beyond observed follow-up data, we also assessed the impact of more conservative assumptions, including no further relative treatment effects beyond five years. Further, the model inputs and assumptions with respect to post-progression monitoring and treatment resource use, were carefully informed through consultation with clinical experts and review of contemporary NHS based cohort studies.

References

1. Public Health Scotland. Scottish health service costs 22/23. https://publichealthscotland.scot/publications/scottish-health-service-costs/scottish-health-service-costs-summary-for-financial-year-2022-to-2023/. Updated 2023. Accessed 03/03, 2023.

2. Jones, Karen C., Burns, Amanda. Unit costs of health and social care 2022. *Personal Social Services Research Unit*. 2023.

3. NHS England. National cost collection for the NHS 21/22. https://www.england.nhs.uk/publication/2021-22-national-cost-collection-data-publication/. Accessed 03/03, 2023.

4. Sivaprasad S, Ghanchi F, Kelly SP, et al. Evaluation of care with intravitreal aflibercept treatment for UK patients with diabetic macular oedema: DRAKO study 24-month real-world outcomes. *Eye (Lond)*. 2023;37(13):2753–2760. doi: 10.1038/s41433-023-02409-y.

5. NICE. British national formulary (BNF). https://bnf.nice.org.uk/ Web site. https://bnf.nice.org.uk/. Accessed 03/03, 2023.

6. Keech AC, Mitchell P, Summanen PA, et al. Effect of fenofibrate on the need for laser treatment for diabetic retinopathy (FIELD study): A randomised controlled trial. *Lancet*. 2007;370(9600):1687–1697. doi: 10.1016/S0140-6736(07)61607-9.

7. Preiss D, Logue J, Sammons E, et al. Effect of fenofibrate on progression of diabetic retinopathy. *NEJM Evid*. 2024;3(8):EVIDoa2400179. doi: 10.1056/EVIDoa2400179.

8. Glassman AR, Wells JA3, Josic K, et al. Five-year outcomes after initial aflibercept, bevacizumab, or ranibizumab treatment for diabetic macular edema (protocol T extension study). *Ophthalmology*. 2020;127(9):1201–1210. doi: 10.1016/j.ophtha.2020.03.021.

9. Fu DJ, Thottarath S, Faes L, et al. Visual acuity outcome of stable proliferative diabetic retinopathy following initial complete panretinal photocoagulation. *BMJ Open Ophth*. 2022;7(1):e001068. http://bmjophth.bmj.com/content/7/1/e001068.abstract. doi: 10.1136/bmjophth-2022-001068.

10. Brazier J, Muston D, Konwea H, et al. Evaluating the relationship between visual acuity and utilities in patients with diabetic macular edema enrolled in intravitreal aflibercept studies. *Invest Ophthalmol Vis Sci*. 2017;58(11):4818–4825. doi: 10.1167/iovs.17-21945.

11. Wells JA, Glassman AR, Ayala AR, et al. Aflibercept, bevacizumab, or ranibizumab for diabetic macular edema: Two-year results from a comparative effectiveness randomized clinical trial. *Ophthalmology*. 2016;123(6):1351–1359. doi: 10.1016/j.ophtha.2016.02.022.

12. Yusuf IH, Henein C, Sivaprasad S. Infographic: Panretinal photocoagulation vs intravitreous ranibizumab for proliferative diabetic retinopathy (PDR): DRCR.net protocol S. *Eye (Lond)*. 2023. doi: 10.1038/s41433-023-02396-0.
